# Genomic-based taxonomic classification of the order Sphingomonadales

**DOI:** 10.1099/ijsem.0.006769

**Published:** 2025-05-14

**Authors:** Yuan Wang, Hao You, Yan-Hui Kong, Cong Sun, Lin-Huan Wu, Song-Gun Kim, Jung-Sook Lee, Lin Xu, Xue-Wei Xu

**Affiliations:** 1 Key Laboratory of Marine Ecosystem Dynamics, Ministry of Natural Resources & Second Institute of Oceanography, Ministry of Natural Resources, Hangzhou 310012, PR China; 2 School of Oceanography, Zhejiang University, Zhoushan 316021, PR China; 3 School of Oceanography, Shanghai Jiao Tong University, Shanghai 200030, PR China; 4 College of Life Sciences and Medicine, Zhejiang Sci-Tech University, Hangzhou 310018, PR China; 5 Institute of Microbiology Chinese Academy of Sciences, Beijing 100101, PR China; 6 Korea Research Institute of Bioscience and Biotechnology, Korean Collection for Type Cultures, Jeongeup 56212, Republic of Korea; 7 National Deep Sea Center, Ministry of Natural Resources, Qingdao 266237, PR China

**Keywords:** average amino acid identity, consensus tree, evolutionary distance, phylogenomic reconstruction, *Sphingomonadales*

## Abstract

The order *Sphingomonadales* strains are globally distributed in various biomes and are renowned for their biodegradable and biosynthesis capabilities. At present, it consists of 4 families and 49 genera making it the third largest order within the class *Alphaproteobacteria*. However, their taxonomy remains complex, especially due to polyphyly in the family *Sphingomonadaceae*. In this study, we collected 429 *Sphingomonadales* type strain genomes, reconstructed robust phylogenomic relationships, and proposed delineation thresholds at the genus and family levels based on average amino acid identities (AAI) and evolutionary distances (ED). Based on the maximum-likelihood and Bayesian phylogenomic trees reconstructed by two molecular sets determined by orthologous sequence identity and the Genome Taxonomy Database, the consensus degree values were all higher than 90%, revealing that those phylogenomic trees had similar topological structures. By confirming monophyletic taxa and determining stable nodes, we reclassified the order *Sphingomonadales* into thirteen families including nine novel ones. AAI calculations indicated that the average intra-family AAI values ranged from 0.62 to 0.84, while inter-family ones were 0.51 to 0.60. ED summaries demonstrated that the average and median intra-family ED values were 0.16 to 0.57, and inter-family ones ranged from 0.50 to 1.22. Comparisons of AAI and ED values calculated by using genomic and phylogenetic analyses supported that those 13 families were significantly separated with *p* values < 2.2×10^−16^. Thus, it was speculated that the AAI and ED thresholds for distinguishing different families were <0.6 and >0.5, respectively. Additionally, we reclassified 163 species into new genera with their phylogenetic topologies, according to the previous genus AAI and ED boundaries of 0.7 and 0.4. Our study is the first genomic-based study of the order *Sphingomonadales* and will promote further insights into the evolution of this order.

## Data Summary

Five supplementary figures and six supplementary tables are available in the FigShare [1] with Figshare link - https://doi.org/10.6084/m9.figshare.28748756.v1

## Introduction

The order *Sphingomonadales* belonging to the phylum *Pseudomonadota*, and the class *Alphaproteobacteria* was proposed by Yabuuchi and Kosako in 2005 [2]. As of August 2024, the order *Sphingomonadales* consists of 4 families and 49 genera and is the third largest order within the class *Alphaproteobacteria*, according to the List of Prokaryotic names with Standing in Nomenclature (https://lpsn.dsmz.de/order/sphingomonadales) [3]. The *Sphingomonadales* cells are commonly Gram-stain-negative rods, cocci, ovoids or pleomorphisms [2,4]. Chemotaxonomically, the major lipids are diphosphatidylglycerol, phosphatidylethanolamine, phosphatidylglycerol and glycosphingolipid, and the predominant fatty acids are C_16:0_, C_17:1_, C_18:1_, C_18:1_* ω*7*c*, C_17:1_* ω*7*c* and/or C_14:0_ 2-OH [2,5]. The *Sphingomonadales* strains are isolated from various habitats, including aquatic [6,10], terrestrial [11,15], host-associated [16,20] and engineered [21,25] biomes. Members of the order *Sphingomonadales* are renowned for their extensive applications in the environment, agriculture, industry and biomedicine, such as degrading organic pollutants [26,28] and synthesizing exopolysaccharides [29,30] and carotenoids [31,32]. Although the *Sphingomonadales* species are ecologically and functionally significant, their taxonomy remains complex, which is urgently needed to be resolved.

In the wake of high-throughput sequencing and genome-based bioinformatic tool developments, phylogenomic trees and overall genome relatedness indices generated from bacterial genomes appear to be better taxonomic indicators than classical ones including 16S rRNA gene sequence analysis and chemotaxonomic determinations [33,35]. The taxonomic status of the order *Sphingomonadales* is revisited recently, with Hördt *et al*. proposing that several species of the family *Sphingomonadaceae* are reclassified into three other families including *Erythrobacteraceae*, *Sphingosinicellaceae* and *Zymomonadaceae* [36]. However, several issues including polyphyletic relationships within one genus and synonyms for different species still challenge the current taxonomic system of the order *Sphingomonadales*.

In this study, we collected 429 *Sphingomonadales* type strain genomes, reconstructed robust phylogenomic relationships, and proposed delineation thresholds at the genus and family levels, which comprehensively revised the taxonomic status of the order *Sphingomonadales*, for performing an improved classification and further investigating their ecological distributions and biotechnological applications.

## Methods

### Collection of *Sphingomonadales* type strain genomes

Eighteen type strains, including *Altererythrobacter aquiaggeris* KCTC 52471^T^, *Altererythrobacter fulvus* KACC 19119^T^, *Altererythrobacter lauratis* KCTC 52606^T^, *Altererythrobacter palmitatis* KCTC 52607^T^, *Rhizorhabdus argentea* DSM 100912^T^, *Sphingobium aquiterrae* DSM 106441^T^, *Sphingobium aromaticiconvertens* DSM 12677^T^, *Sphingobium mellinum* DSM 7458^T^, *Sphingomonas agri* LMG 29563^T^, *Sphingomonas canadensis* DSM 29393^T^, *Sphingomonas kyungheensis* LMG 26582^T^, *Sphingomonas panni* DSM 15761^T^, *Sphingomicrobium aestuariivivum* KCTC 42286^T^, *Sphingomicrobium arenosum* NBRC 113094^T^, *Sphingomicrobium flavum* JCM 18555^T^, *Sphingomicrobium astaxanthinifaciens* JCM 18551^T^, *Sphingomicrobium lutaoense* DSM 24194^T^ and *Sphingomicrobium marinum* JCM 18554^T^, were obtained from four culture collections including the Deutsche Sammlung von Mikroorganismen und Zellkulturen GmbH (DSMZ), the Korean Agricultural Culture Collection (KACC), the Korean Collection for Type Cultures (KCTC) and the Collection of the Laboratorium voor Microbiologie en Microbiele Genetica (LMG). Genomic DNAs of those type strains were extracted by using AxyPre Bacterial Genomic DNA Miniprep Kit (Axygen Biosciences, Union City, CA, USA) according to its manual, when type strains were cultivated under the appropriate conditions proposed previously [37,48]. Genomic sequencing and assembly were performed as described previously [5]. Genomes were sequenced on the HiSeq 2000 system (Illumina) by Solexa paired-end sequencing technology with a paired-end library with insert length of 500 bp in the Novogene Corporation (Beijing, China). Draft genomes were assembled by using SPAdes version 3.10.1 [49] based on clean reads generated from raw reads by quality trimming.

Four genomes of *Chioneia* type strains including *Chioneia algoris* R-39594^T^ (Ga0427589), *Chioneia brumae* R-39161^T^ (Ga0427977), *Chioneia frigida* R-67880^T^ (Ga0401290) and *Chioneia hiemis* R-36677^T^ (Ga0401292) were obtained from Integrated Microbial Genomes and Microbiomes (IMG) [50], while seven genomes of type strains comprising *Novosphingobium bradum* KCTC 42984^T^ (GCM10019133), *Novosphingobium ipomoeae* KCTC 42656^T^ (GCM10017100), *Sphingoaurantiacus capsulatus* KCTC 42644^T^ (GCM10017054), *Sphingomonas arantia* CGMCC 1.12702^T^ (GCM10020237), *Sphingomonas floccifaciens* CGMCC 1.15797^T^ (GCM10020312), *Sphingomonas hylomeconis* KCTC 42739^T^ (GCM10017173) and *Sphingomonas qilianensis* KCTC 42862^T^ (GCM10017360) were received from the Global Catalogue of Type Strain (gcType) database [51]. Other types strain genomes were obtained from the NCBI GenBank database. Detailed genomic information including assembly numbers, genomic sizes and genomic G+C contents was listed in Table S1.

### Genomic annotation and 16S rRNA gene phylogenetic reconstruction

Genomic qualities including completeness and contamination were assessed using the CheckM v.1.10.1 [52]. Coding sequences (CDs) as well as tRNA and rRNA genes of each genome were predicted by using Rapid Annotation using Subsystem Technology (RAST) web server version 2.0 (https://rast.nmpdr.org/) [53]. Retrieved 16S rRNA gene sequences were used to compare their sequence identities on the EzBioCloud web server (www.ezbiocloud.net/identify) [54] to confirm that the genome represented its corresponding type strain. The 16S rRNA gene sequences of 429 *Sphingomonadales* type strains and an outgroup (*Rhodospirillum rubrum* ATCC 11170^T^) were aligned using Clustal W [55] embedded in the mega11 software [56]. Subsequently, these aligned sequences were subjected to a maximum-likelihood phylogenetic analysis [57] using the mega11 software [56], with the nucleotide substitution model selected as Kimura’s two-parameter model and the bootstrap value set as 1000 replicates.

### Comparative genomic and phylogenomic analysis

Two gene sets including the bac120 gene set generated from the Genome Taxonomy Database (GTDB) [58] by using GTDB-Tk v.2.3.2 [59] with the workflow of classify_wf and the sp22 gene set containing shared single-copy orthologous clusters (OCs) analysed by using OrthoFinder v.2.5.4 [60] with the default settings were obtained for our phylogenomic reconstructions. Those protein sequences were aligned by using MAFFT v.7.471 [61] with the ‘-auto’ parameter, and then were refined to select the most reliable positions through trimAL v.1.4.1 [62] with the parameter ‘-automated1’. Maximum-likelihood and Bayesian phylogenetic reconstructions were both performed in this study. Maximum-likelihood phylogenomic trees were reconstructed by using IQ-TREE v.1.6.2 [63], with the best amino acid substitution model estimated as LG+F+R10 and the bootstrap value set as 1000 replicates. Bayesian phylogenomic trees were generated using the Bayesian tree-building workflow in PhyloSuite v.1.2.3 [64]. The consensus degree (CD) indicating the percentage of identical nodes among those four phylogenomic trees was calculated pairwise as recommended by Zhang *et al*. [65]. All the phylogenetic trees and the cladograms were displayed and edited using the online tool Interactive Tree Of Life (iTOL) v.6 (https://itol.embl.de/).

### Whole-genome relatedness indices

Overall genome relatedness indices including average amino acid identity (AAI), average nucleotide identity (ANI) and digital DNA–DNA hybridization (dDDH) values were used to calculate genomic similarities pairwise. The AAI values were deduced from pairwise conserved comparisons of coding proteins by using the AAI calculator web server (http://enve-omics.ce.gatech.edu/aai/). The ANI values were calculated by the fastANI v.1.33 using alignment-free approximate sequence mapping [66]. The dDDH values were determined by using Genome-to-Genome Distance Calculator v.3.0 web server (https://ggdc.dsmz.de/ggdc.php) [67]. Phylogenetically, the evolutionary distance (ED) values were retrieved from the output ‘mldist’ file generated by using IQ-TREE v.1.6.2 [63]. In addition, the *t*-tests of those values among inter- and intra-group were calculated by using the function ‘t.test’ within R software v.4.2.0, and the boxplot diagrams of ED and AAI values were displayed using the ggplot2 package in the R software v.4.2.0.

## Results and discussion

### Genomics characteristics of *Sphingomonadales* type strains

After genomic sequencing and assembly, 17 type strain genomes obtained in this study were estimated as high-quality ones with the completeness of 97.9–99.8% and the contamination of 0–2.1% (Table 1), which exceeded genome reporting standards for high-quality assembled genomes (the completeness of >90% and the contamination of <5%) [68]. Combined with the rest of *Sphingomonadales* type strain genomes obtained from public databases, we collected a total of 429 genomes. The collection of assembled and obtained genomes covered 91% (170/187), 86% (236/274), 88% (22/25) and 100% (1/1) of the families *Erythrobacteraceae*, *Sphingomonadaceae*, *Sphingosinicellaceae* and *Zymomonadaceae* type strains, respectively. Moreover, this genome collection confirmed at least one type strain genome of each *Sphingomonadales* genus was included. Detailed information is listed in Table S1, available in the online Supplementary Material.

**Table 1.** Genomic qualities and information of assembled *Sphingomonadales* type strains in this study

Type strain	Completeness (%)	Contamination (%)	Contigs	Size (bp)	G+C content (%)
*Altererythrobacter aquiaggeris* KCTC 52471^T^	99.6	0.2	2	2644226	58.8
*Altererythrobacter fulvus* KACC 19119^T^	99.5	0.9	11	3516177	63.8
*Altererythrobacter lauratis* KCTC 52606^T^	99.0	0.1	32	3095759	64.5
*Altererythrobacter palmitatis* KCTC 52607^T^	98.7	0.1	22	3208183	64.4
*Rhizorhabdus argentea* DSM 100912^T^	99.1	0.5	56	4283009	63.6
*Sphingobium aquiterrae* DSM 106441^T^	99.7	1.4	55	4995461	64.9
*Sphingobium aromaticiconvertens* DSM 12677^T^	99.8	2.1	6	5453086	62.2
*Sphingobium mellinum* DSM 7458^T^	99.1	0.9	53	3767061	63.2
*Sphingomonas agri* LMG 29563^T^	99.8	0.7	4	2868910	62.7
*Sphingomonas kyungheensis* LMG 26582^T^	99.3	0.5	28	4055944	68.3
*Sphingomonas panni* DSM 15761^T^	99.6	0.3	22	4187984	66.4
*Sphingomicrobium aestuariivivum* KCTC 42286^T^	98.4	0.2	1	2345960	66.4
*Sphingomicrobium arenosum* NBRC 113094^T^	98.4	0.2	3	2558758	65.4
*Sphingomicrobium astaxanthinifaciens* JCM 18551^T^	97.9	0.2	3	2249836	68.3
*Sphingomicrobium flavum* JCM 18555^T^	98.5	0	1	2339771	63.0
*Sphingomicrobium lutaoense* DSM 24194^T^	98.4	0	2	2079293	63.9
*Sphingomicrobium marinum* JCM 18554^T^	98.0	0	2	2238205	62.3

Genomic sizes, gene counts and G+C contents were 2.07–7.00 Mbp (average: 3.74 ± 0.87 Mbp; median: 3.64 Mbp), 1869.00–7045.00 (average 3567.00 ± 805.99; median 3465.00) and 46.06–71.80% (average 64.00% ± 5.48; median 64.78%), respectively (Table S1). A total of 22 single-copy OCs (Table S2) were included in our phylogenomic analyses. Furthermore, overall genome relatedness index calculations indicated that three pairs of *Sphingomonadales* type strain genomes (*Sphingobium baderi* LL03^T^ and *Sphingobium wenxiniae* CGMCC 1.7748^T^; *Sphingomonas haloaromaticamans* CCUG 53463^T^ and *Sphingomonas fennica* K101^T^; *Qipengyuania aerophila* GH25^T^ and *Qipengyuania pacifica* NZ-96^T^) shared ANI values of >96% and dDDH values of >70% (Tables S3 and S4), which were higher than species thresholds (95–96 and 70% for ANI and dDDH, respectively) [35]. The 16S rRNA gene sequence identities among those three pairs of type strains were 99.2% (*Sphingobium baderi* LL03^T^ and *Sphingobium wenxiniae* JZ-1^T^), 96.0% (*Sphingomonas fennica* K101^T^ and *Sphingomonas haloaromaticamans* A175^T^) and 100.0% (*Qipengyuania aerophila* GH25^T^ and *Qipengyuania pacifica* NZ-96^T^). Moreover, phenotypic comparisons revealed that *Qipengyuania aerophila* GH25^T^ and *Qipengyuania pacifica* NZ-96^T^ shared a wide variety of phenotypic characteristics including positive for catalase, oxidase, acid and alkaline phosphatase, leucine arylamidase, valine arylamidase, cystine arylamidase, trypsin and naphthol-AS-BI-phosphohydrolase activities, hydrolysis of Tween 40, negative for nitrate reduction, indole production, arginine dihydrolase, α- and β-Galactosidase, β-glucuronidase, β-glucosidase, *N*-acetyl-β-glucosaminidase, α-mannosidase and α-fucosidase activities [10,69]. Whereas a wide range of characteristics including carbon substrate utilization and hydrolyses can be applied to distinguish *Sphingobium baderi* LL03^T^ from *Sphingobium wenxiniae* JZ-1^T^, and *Sphingomonas fennica* K101^T^ from *Sphingomonas haloaromaticamans* A175^T^ [70,72]. Consequently, *Qipengyuania aerophila* is recommended to be reclassified as a later heterotypic synonym of *Qipengyuania pacifica*.

### 16S rRNA gene sequence phylogeny of the order *Sphingomonadales*

The two major families *Erythrobacteraceae* and *Sphingomonadaceae* were not separated independently in the phylogenetic tree based on the 16S rRNA gene sequences (Fig. S1). Numerous genera including *Allosphingosinicella*, *Altererythrobacter*, *Alteriqipengyuania*, *Aurantiacibacter*, *Blastomonas*, *Chioneia*, *Croceibacterium*, *Croceicoccus*, *Erythrobacter*, *Hephaestia*, *Novosphingobium*, *Parerythrobacter*, *Pelagerythrobacter*, *Pontixanthobacter*, *Qipengyuania*, *Rhizorhabdus*, *Sandaracinobacteroides*, *Sphingobium*, *Sphingomonas*, *Sphingorhabdus*, *Sphingosinicella*, *Stakelama* and *Tsuneonella* were also polyphyletic groups. Moreover, most of the nodes (75.0%, 319/425) exhibited bootstrap values lower than 70% indicating that this phylogenetic tree was not robust enough, which was similar to the previous study of the family *Erythrobacteraceae* [5]. Therefore, the 16S rRNA gene cannot be an effective gene marker for accurately classifying the order *Sphingomonadales*, and their taxonomic status and phylogenetic relationship should be resolved by using genomic data.

### Phylogenomic consensus of the order *Sphingomonadales*

**Fig. 1.**
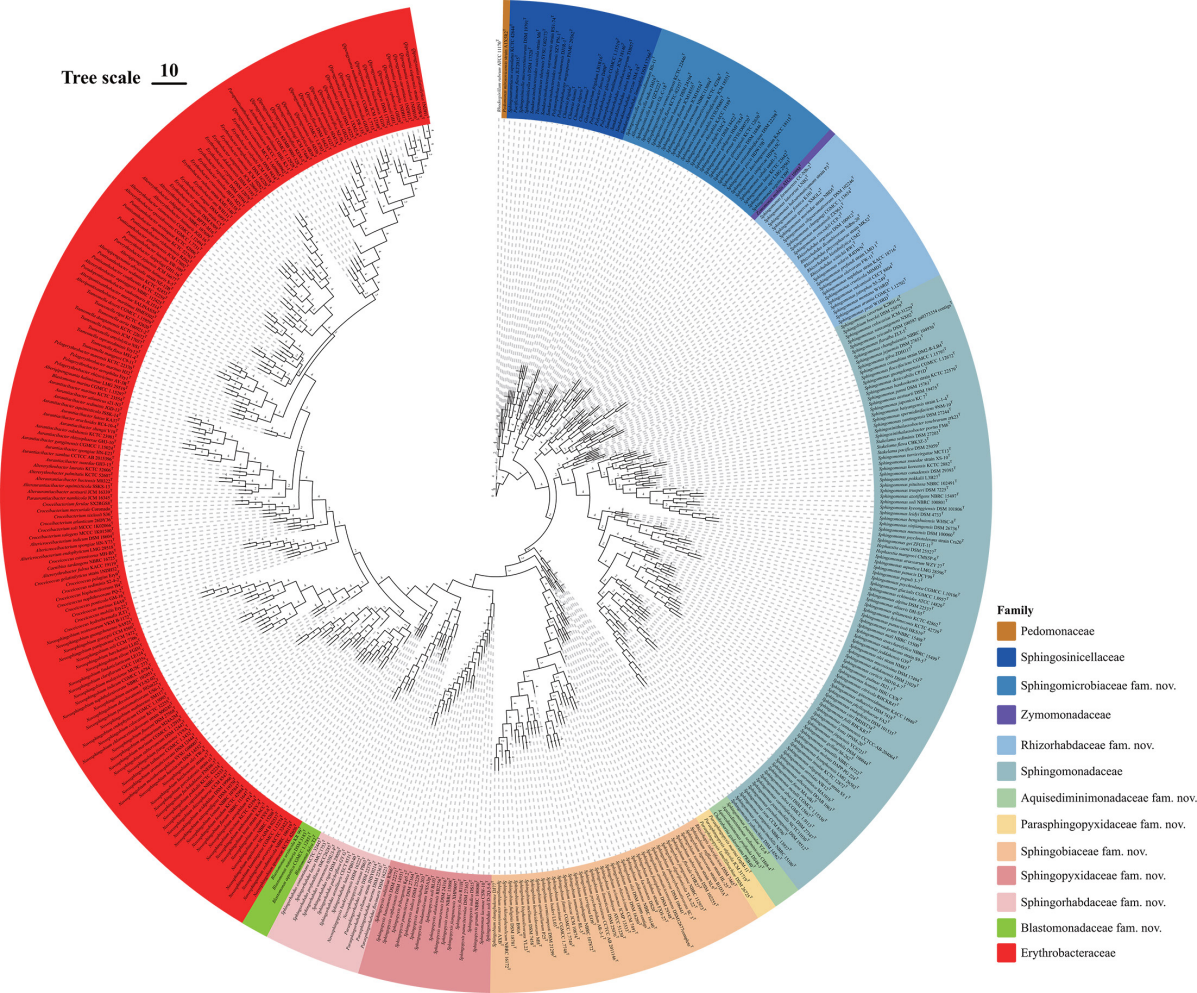
The census tree based on maximum-likelihood and Bayesian phylogenomic trees reconstructed by two gene sets illustrated the phylogenetic relationship of the order *Sphingomonadales*. The node values indicated that the recoveries in the maximum-likelihood and Bayesian phylogenomic tree based on two gene sets, which were shown in Fig. S2** to S5**.

Comparative genomic analysis revealed that the pangenome of the order *Sphingomonadales* harboured 27042 OCs, among which one molecular set (sp22) including 22 single-copy OCs was detected across all *Sphingomonadales* genomes as well as the outgroup *Rhodospirillum rubrum* ATCC 11170^T^ (Table S2). Another molecular set bac120 was generated based on the Genome Taxonomy Database. Based on four phylogenomic trees reconstructed by two molecular sets and two methods, the CD values were all higher than 90% revealing that those phylogenomic trees had similar topological structures (Fig. 1). Furthermore, the bootstrap values of most nodes in those two ML phylogenomic trees exceeded 70%, indicating those phylogenies were robust. Compared with 16S rRNA gene phylogeny, those similar and robust phylogenomic trees could provide a reliable taxonomic status for the order *Sphingomonadales*.

By confirming monophyletic taxa and determining stable nodes, we classified 13 independent clades including clade 1 (1 *Pedomonas* species), clade 2 (4 *Chioneia* species, 1 *Glacieibacterium* species, 1 *Pacificimonas* species, 5 *Polymorphobacter* species, 3 *Sandaracinobacteroides* species, 4 *Sandarakinorhabdus* species, 1 *Sphingoaurantiacus* species, 2 *Sphingosinicella* species, 1 *Thermaurantiacus* species), clade 3 (2 *Allosphingosinicella* species, 6 *Sphingomicrobium* species, 17 *Sphingomonas* species, 3 *Sphingosinicella* species), clade 4 (1 *Zymomonas* species), clade 5 (5 *Rhizorhabdus* species, 20 *Sphingomonas* species), clade 6 (2 *Hephaestia* species, 1 *Sphingobium* species, 89 *Sphingomonas* species, 2 *Sphingosinithalassobacter* species, 3 *Stakelama* species), clade 7 (2 *Aquisediminimonas* species, 1 *Chakrabartia* species, 1 *Sphingomonas* species), clade 8 (3 *Parasphingopyxis* species), clade 9 (1 *Rhizorhapis* species, 39 *Sphingobium* species), clade 10 (1 *Sphingomonas* species, 17 *Sphingopyxis* species, 1 *Sphingorhabdus* species), clade 11 (2 *Novosphingopyxis* species, 4 *Parasphingorhabdus* species, 8 *Sphingorhabdus* species), clade 12 (4 *Blastomonas* species), clade 13 (1 *Actirhodobacter* species, 3 *Alteraurantiacibacter* species, 9 *Altererythrobacter* species, 3 *Altericroceibacterium* species, 2 *Alteripontixanthobacter* species, 3 *Alteriqipengyuania* species, 13 *Aurantiacibacter* species, 1 *Blastomonas* species, 1 *Caenibius* species, 6 *Croceibacterium* species, 10 *Croceicoccus* species, 15 *Erythrobacter* species, 51 *Novosphingobium* species, 1 *Parapontixanthobacter* species, 1 *Paraurantiacibacter* species, 2 *Parerythrobacter* species, 4 *Pelagerythrobacter* species, 7 *Pontixanthobacter* species, 1 *Pseudopontixanthobacter* species, 28 *Qipengyuania* species, 9 *Tsuneonella* species). Except for clades 2, 4 and 13 mainly represented by the families *Sphingosinicellaceae*, *Zymomonadaceae* and *Erythrobacteraceae*, other clades could be considered as respective taxonomic groups at the family level. Therefore, the original family *Sphingomonadaceae* members are suggested to be reclassified into 11 families, among which eight clades could represent new ones. Detailed reclassification is shown in Table 2.

**Table 2.** Resulting classification within the order *Sphingomonadales* following our study. Novel names were coloured in red and original species names for combinations were bracketed. Family names, without the indentation; genus names, with indentation of four spaces; species names, with indentation of eight spaces

Clade	Family/Genus/Species
Clade 1	*Pedomonaceae* fam. nov. *Pedomonas* *Pedomonas mirosovicensis*
Clade 2	*Sphingosinicellaceae Glacieibacterium* *Glacieibacterium algoris* comb. nov. *(Chioneia algoris)* *Glacieibacterium arshaanense* comb. nov. *(Polymorphobacter arshaanensis)* *Glacieibacterium brumae* comb. nov. *(Chioneia brumae) Glacieibacterium frigidum* *Glacieibacterium frigoris* nom. nov. *(Chioneia frigida)* *Glacieibacterium hiemis* comb. nov. *(Chioneia hiemis)* *Glacieibacterium megasporae* comb. nov. *(Polymorphobacter megasporae)Neosandaracinobacteroides* gen. nov. *Neosandaracinobacteroides saxicola* comb. nov. *(Sandaracinobacteroides saxicola)* *Pacificimonas Pacificimonas flava Sandaracinobacteroides Sandaracinobacteroides hominis Sandaracinobacteroides sayramensisSandarakinorhabdus Sandarakinorhabdus cyanobacteriorum* *Sandarakinorhabdus fusca* comb. nov. *(Polymorphobacter fuscus)* *Sandarakinorhabdus glacialis* comb. nov. *(Polymorphobacter glacialis)* *Sandarakinorhabdus limnophila* *Sandarakinorhabdus multimanifer* comb. nov. (*Polymorphobacter multimanifer*) *Sandarakinorhabdus oryzae Sandarakinorhabdus rubra Sphingoaurantiacus Sphingoaurantiacus capsulatus Sphingosinicella Sphingosinicella microcystinivorans Sphingosinicella soli Thermaurantiacus Thermaurantiacus tibetensis*
Clade 3	*Sphingomicrobiaceae* fam. nov.*AllosphingosinicellaAllosphingosinicella deserti* comb. nov. *(Sphingomonas deserti)Allosphingosinicella flava* comb. nov. *(Sphingosinicella flava)Allosphingosinicella ginsenosidimutans* comb. nov. *(Sphingosinicella ginsenosidimutans) Allosphingosinicella humi* comb. nov. *(Sphingosinicella humi) Allosphingosinicella indica Allosphingosinicella vermicomposti Pseudosphingomonas* gen. nov.* Pseudosphingomonas agri* comb. nov. *(Sphingomonas agri) Pseudosphingomonas arenae* comb. nov. *(Sphingomonas arenae) Pseudosphingomonas astaxanthinifaciens* comb. nov. *(Sphingomonas astaxanthinifaciens) Pseudosphingomonas daechungensis* comb. nov. *(Sphingomonas daechungensis) Pseudosphingomonas edaphi* comb. nov. *(Sphingomonas edaphi) Pseudosphingomonas ginkgonis* comb. nov. *(Sphingomonas ginkgonis) Pseudosphingomonas ginsengisoli* comb. nov. *(Sphingomonas ginsengisoli) Pseudosphingomonas jaspsi* comb. nov. *(Sphingomonas jaspsi) Pseudosphingomonas kaistensis* comb. nov. *(Sphingomonas kaistensis) Pseudosphingomonas lutea* comb. nov. *(Sphingomonas lutea) Pseudosphingomonas mesophila* comb. nov. *(Sphingomonas mesophila) Pseudosphingomonas piscis* comb. nov. *(Sphingomonas piscis) Pseudosphingomonas rhizophila* comb. nov. *(Sphingomonas rhizophila) Pseudosphingomonas sabuli* comb. nov. *(Sphingomonas sabuli) Pseudosphingomonas segetis* comb. nov. *(Sphingomonas segetis) Pseudosphingomonas sinipercae* comb. nov. *(Sphingomonas sinipercae) Sphingomicrobium Sphingomicrobium aestuariivivum Sphingomicrobium arenosum Sphingomicrobium astaxanthinifaciens Sphingomicrobium flavum Sphingomicrobium lutaoense Sphingomicrobium marinum*
Clade 4	*Zymomonadaceae Zymomonas Zymomonas mobilis*
Clade 5	*Rhizorhabdaceae* fam. nov.* Alterirhizorhabdus* gen. nov.* Alterirhizorhabdus profundi* comb. nov. *(Sphingomonas profundi) Alterirhizorhabdus solaris* comb. nov. *(Sphingomonas solaris) Edaphosphingomonas* gen. nov.* Edaphosphingomonas fennica* comb. nov. *(Sphingomonas fennica) Edaphosphingomonas haloaromaticamans* comb. nov. *(Sphingomonas haloaromaticamans) Edaphosphingomonas laterariae* comb. nov. *(Sphingomonas laterariae) Flavisphingomonas*gen. nov.* Flavisphingomonas formosensis* comb. nov. *(Sphingomonas formosensis)Neorhizorhabdus gen. nov. Neorhizorhabdus crusticola* comb. nov. *(Sphingomonas crusticola) Neorhizorhabdus naphthae* comb. nov. *(Sphingomonas naphthae) Neorhizorhabdus oleivorans* comb. nov. *(Sphingomonas oleivorans) Neorhizorhabdus vulcanisoli* comb. nov. *(Sphingomonas vulcanisoli) Pararhizorhabdusgen. nov. Pararhizorhabdus arantia* comb. nov. *(Sphingomonas arantia)Pararhizorhabdus jatrophae* comb. nov. *(Sphingomonas jatrophae)Pararhizorhabdus montana* comb. nov. *(Sphingomonas montana)Pararhizorhabdus prati* comb. nov. *(Sphingomonas prati) Rhizorhabdus Rhizorhabdus argentea Rhizorhabdus crocodyli* comb. nov. *(Sphingomonas crocodyli) Rhizorhabdus dicambivorans Rhizorhabdus histidinilytica Rhizorhabdus montanisoli* comb. nov. *(Sphingomonas montanisoli) Rhizorhabdus phycosphaerae Rhizorhabdus wittichii Solisphingomonas* gen. nov.* Solisphingomonas chungangi* comb. nov. *(Sphingomonas chungangi) Solisphingomonas morindae* comb. nov. *(Sphingomonas morindae) Solisphingomonas oligoaromativorans* gen. nov, comb. nov. *(Sphingomonas oligoaromativorans) Solisphingomonas quercus* comb. nov. *(Sphingomonas quercus)*
Clade 6	*Sphingomonadaceae Alterisphingomonas* gen. nov.* Alterisphingomonas asaccharolytica* comb. nov. *(Sphingomonas asaccharolytica) Alterisphingomonas mali* comb. nov. *(Sphingomonas mali) Alterisphingomonas panacisoli* comb. nov. *(Sphingomonas panacisoli) Alterisphingomonas pruni* comb. nov. *(Sphingomonas pruni) Alterisphingomonas radiodurans* comb. nov. *(Sphingomonas radiodurans) Alteristakelama* gen. nov.* Alteristakelama azotifigens* comb. nov. *(Sphingomonas azotifigens) Alteristakelama canadensis* comb. nov. *(Sphingomonas canadensis) Alteristakelama gei* comb. nov. *(Sphingomonas gei) Alteristakelama hengshuiensis* comb. nov. *(Sphingomonas hengshuiensis) Alteristakelama koreensis* comb. nov. *(Sphingomonas koreensis) Alteristakelama kyeonggiensis* comb. nov. *(Sphingomonas kyeonggiensis) Alteristakelama leidyi* comb. nov. *(Sphingomonas leidyi) Alteristakelama naasensis* comb. nov. *(Sphingomonas naasensis) Alteristakelama pituitosa* comb. nov. *(Sphingomonas pituitosa) Alteristakelama pokkalii* comb. nov. *(Sphingomonas pokkalii) Alteristakelama psychrotolerans* comb. nov. *(Sphingomonas psychrotolerans) Alteristakelama soli* comb. nov. *(Sphingomonas soli) Alteristakelama suaedae* comb. nov. *(Sphingomonas suaedae) Alteristakelama trueperi* comb. nov. *(Sphingomonas trueperi) Alteristakelama turrisvirgatae* comb. nov. *(Sphingomonas turrisvirgatae) Alteristakelama xinjiangensis* comb. nov. *(Sphingomonas xinjiangensis) Alteriyabuuchia* gen. nov.* Alteriyabuuchia sanxanigenens* comb. nov. *(Sphingomonas sanxanigenens) Alteriyabuuchia zeicaulis* comb. nov. *(Sphingomonas zeicaulis) Hephaestia Hephaestia caeni Hephaestia mangrovi Humisphingomonas* gen. nov.* Humisphingomonas gilva* comb. nov. *(Sphingomonas gilva) Novistakelama* gen. nov.* Novistakelama desiccabilis* comb. nov. *(Sphingomonas desiccabilis) Novistakelama hankookensis* comb. nov. *(Sphingomonas hankookensis) Novistakelama panni* comb. nov. *(Sphingomonas panni) Parasphingomonas* gen. nov.* Parasphingomonas aliaeris* comb. nov. *(Sphingomonas aliaeris) Parasphingomonas alpina* comb. nov. *(Sphingomonas alpina) Parasphingomonas aquatica* comb. nov. *(Sphingomonas aquatica) Parasphingomonas aracearum* comb. nov. *(Sphingomonas aracearum) Parasphingomonas echinoides* comb. nov. *(Sphingomonas echinoides) Parasphingomonas glacialis* comb. nov. *(Sphingomonas glacialis) Parasphingomonas hylomeconis* comb. nov. *(Sphingomonas hylomeconis) Parasphingomonas panacis* comb. nov. *(Sphingomonas panacis) Parasphingomonas populi* comb. nov. *(Sphingomonas populi) Parasphingomonas psychrolutea* comb. nov. *(Sphingomonas psychrolutea) Parasphingomonas qilianensis* comb. nov. *(Sphingomonas qilianensis) Parastakelama* gen. nov.* Parastakelama aestuarii* comb. nov. *(Sphingomonas aestuarii) Parastakelama baiyangensis* comb. nov. *(Sphingomonas baiyangensis) Parastakelama japonica* comb. nov. *(Sphingomonas japonica) Parastakelama spermidinifaciens* comb. nov. *(Sphingomonas spermidinifaciens) Parastakelama yantingensis* comb. nov. *(Sphingomonas yantingensis) Parayabuuchia* gen. nov.* Parayabuuchia changbaiensis* comb. nov. *(Sphingomonas changbaiensis) Parayabuuchia flavalba* comb. nov. *(Sphingomonas flavalba) Parayabuuchia jejuensis* comb. nov. *(Sphingomonas jejuensis) Pseudostakelama* gen. nov.* Pseudostakelama cannabina* comb. nov. *(Sphingomonas cannabina) Pseudostakelama floccifaciens* comb. nov. *(Sphingomonas floccifaciens) Pseudostakelama guangdongensis* comb. nov. *(Sphingomonas guangdongensis) Sphingomonas Sphingomonas abaci Sphingomonas adhaesiva Sphingomonas aerolata Sphingomonas aerophila Sphingomonas albertensis Sphingomonas aquatilis Sphingomonas aurantiaca Sphingomonas carotinifaciens Sphingomonas citri Sphingomonas citricola Sphingomonas corticis*
	* Sphingomonas dokdonensis Sphingomonas endophytica Sphingomonas faeni Sphingomonas folli Sphingomonas gellani Sphingomonas ginsenosidimutans Sphingomonas hominis Sphingomonas insulae Sphingomonas jeddahensis Sphingomonas jinjuensis Sphingomonas kyungheensis Sphingomonas lenta Sphingomonas melonis Sphingomonas metalli Sphingomonas mucosissima Sphingomonas olei Sphingomonas oligophenolica Sphingomonas palmae Sphingomonas parapaucimobilis Sphingomonas paucimobilis Sphingomonas phyllosphaerae Sphingomonas pseudosanguinis Sphingomonas rubra Sphingomonas sanguinis Sphingomonas yabuuchiae Sphingomonas yunnanensis Sphingomonas zeae Stakelama Stakelama flava Stakelama pacifica Stakelama portus* comb. nov. *(Sphingosinithalassobacter portus) Stakelama sediminis Stakelama tenebrarum* comb. nov. *(Sphingosinithalassobacter tenebrarum) Yabuuchia* gen. nov.* Yabuuchia boeckii* comb. nov. *(Sphingobium boeckii) Yabuuchia cavernae* comb. nov. *(Sphingomonas cavernae) Yabuuchia colocasiae* comb. nov. *(Sphingomonas colocasiae)*
Clade 7	*Aquisediminimonadaceae* fam. nov.* Alteraquisediminimonas* gen. nov.*Alteraquisediminimonas sediminicola* comb. nov. *(Aquisediminimonas sediminicola) Aquisediminimonas Aquisediminimonas godavariana* comb. nov. *(Chakrabartia godavariana) Aquisediminimonas profunda Paraquisediminimonas* gen. nov.* Paraquisediminimonas paeninsulae* comb. nov. *(Sphingomonas paeninsulae)*
Clade 8	*Parasphingopyxidaceae* fam. nov. * Parasphingopyxis Parasphingopyxis algicola Parasphingopyxis lamellibrachiae Parasphingopyxis marina*
Clade 9	*Sphingobiaceae* fam. nov.* Alterisphingobium* gen. nov.* Alterisphingobium fluviale* comb. nov. *(Sphingobium fluviale) Alterisphingobium jiangsuense* comb. nov. *(Sphingobium jiangsuense) Alterisphingobium subterraneum* comb. nov. *(Sphingobium subterraneum) Parasphingobium* gen. nov.* Parasphingobium lignivorans* comb. nov. *(Sphingobium lignivorans) Parasphingobium sufflavum* comb. nov. *(Sphingobium sufflavum) Parasphingobium xanthum* comb. nov. *(Sphingobium xanthum) Rhizorhapis Rhizorhapis suberifaciens Sphingobium Sphingobium abikonense Sphingobium algorifonticola Sphingobium amiense Sphingobium aquiterrae Sphingobium aromaticiconvertens Sphingobium baderi Sphingobium bisphenolivorans Sphingobium chlorophenolicum Sphingobium chungbukense Sphingobium cloacae Sphingobium cupriresistens Sphingobium estronivorans Sphingobium faniae Sphingobium fontiphilum Sphingobium fuliginis Sphingobium herbicidovorans Sphingobium indicum Sphingobium lactosutens Sphingobium limneticum Sphingobium mellinum Sphingobium naphthae Sphingobium olei Sphingobium phenoxybenzoativorans Sphingobium psychrophilum Sphingobium quisquiliarum Sphingobium rhizovicinum Sphingobium scionense Sphingobium terrigena Sphingobium ummariense Sphingobium vermicomposti Sphingobium wenxiniae Sphingobium xenophagum Sphingobium yanoikuyae*
Clade 10	*Sphingopyxidaceae* fam. nov.* Alterisphingopyxis* gen. nov.* Alterisphingopyxis lacunae* comb. nov. *(Sphingomonas lacunae) Flavisphingopyxis* gen. nov.* Flavisphingopyxis soli* comb. nov. *(Sphingorhabdus soli) Sphingopyxis Sphingopyxis alaskensis Sphingopyxis bauzanensis Sphingopyxis flava Sphingopyxis fribergensis Sphingopyxis granuli Sphingopyxis indica Sphingopyxis italica Sphingopyxis jiangsuensis Sphingopyxis lindanitolerans Sphingopyxis macrogoltabida Sphingopyxis panaciterrae Sphingopyxis panaciterrulae Sphingopyxis soli Sphingopyxis solisilvae Sphingopyxis terrae Sphingopyxis ummariensis Sphingopyxis witflariensis*
Clade 11	*Sphingorhabdaceae* fam. nov.* Alterisphingorhabdus* gen. nov.* Alterisphingorhabdus lutea* comb. nov. *(Sphingorhabdus lutea) Novosphingopyxis Novosphingopyxis baekryungensis Novosphingopyxis iocasae Parasphingorhabdus Parasphingorhabdus cellanae Parasphingorhabdus flavimaris Parasphingorhabdus litoris Parasphingorhabdus marina Sphingorhabdus Sphingorhabdus arenilitoris Sphingorhabdus contaminans Sphingorhabdus lacus Sphingorhabdus profundilacus Sphingorhabdus pulchriflava Sphingorhabdus rigui Sphingorhabdus wooponensis*
Clade 12	*Blastomonadaceae fam. nov. Blastomonas Blastomonas aquatica Blastomonas fulva Blastomonas natatoria Blastomonas ursincola*
Clade 13	*Erythrobacteraceae* *Aestuarierythrobacter* gen. nov*.* * Aestuarierythrobacter aquiaggeris* comb. nov. *(Altererythrobacter aquiaggeris)* * Aestuarierythrobacter confluentis* comb. nov. *(Pontixanthobacter confluentis)* * Aestuarierythrobacter sediminis* comb. nov. *(Pontixanthobacter sediminis)* *Agrinovosphingobium* gen. nov. * Agrinovosphingobium ipomoeae* comb. nov. *(Novosphingobium ipomoeae)* * Alteraurantiacibacter* * Alteraurantiacibacter aestuarii* * Alteraurantiacibacter aquimixticola* * Alteraurantiacibacter buctensis* * Alteraurantiacibacter lauratis* comb. nov. *(Altererythrobacter lauratis)* * Alteraurantiacibacter palmitatis* comb. nov. *(Altererythrobacter palmitatis)* * Altererythrobacter* * Altererythrobacter epoxidivorans* * Altererythrobacter insulae* * Altererythrobacter ishigakiensis* * Altererythrobacter lutimaris* * Altererythrobacter xiamenensis* * Altericroceibacterium* * Altericroceibacterium endophyticum* * Altericroceibacterium indicum* * Altericroceibacterium spongiae* * Alterinovosphingobium* gen. nov. *Alterinovosphingobium aerophilum* cAlterisphingorhabdusomb. nov. *(Novosphingobium aerophilum)* * Alterinovosphingobium bradum* comb. nov. *(Novosphingobium bradum)* * Alterinovosphingobium flavum* comb. nov. *(Novosphingobium flavum)* * Alterinovosphingobium humi* comb. nov. *(Novosphingobium humi)* * Alterinovosphingobium ovatum* comb. nov. *(Novosphingobium ovatum)* * Alterinovosphingobium piscinae* comb. nov. *(Novosphingobium piscinae)* * Alterinovosphingobium rosa* comb. nov. *(Novosphingobium rosa)* * Alterinovosphingobium umbonatum* comb. nov. *(Novosphingobium umbonatum)* * Alteripontixanthobacter* * Alteripontixanthobacter maritimus* * Alteriqipengyuania* * Alteriqipengyuania abyssalis* * Alteriqipengyuania lutimaris* * Aurantiacibacter* * Aurantiacibacter aquimixticola* * Aurantiacibacter arachoides* * Aurantiacibacter atlanticus* * Aurantiacibacter gangjinensis* * Aurantiacibacter luteus* * Aurantiacibacter marinus* * Aurantiacibacter odishensis* * Aurantiacibacter rhizosphaerae* * Aurantiacibacter sediminis* * Aurantiacibacter spongiae* * Aurantiacibacter suaedae* * Aurantiacibacter xanthus* * Aurantiacibacter zhengii* * Caenibius* * Caenibius estronivorus* comb. nov. *(Croceicoccus estronivorus)* * Caenibius fulvus* comb. nov. *(Altererythrobacter fulvus)* * Caenibius tardaugens* * Croceibacterium* * Croceibacterium atlanticum* * Croceibacterium ferulae* * Croceibacterium mercuriale* * Croceibacterium salegens* * Croceibacterium soli* * Croceibacterium xixiisoli* * Croceicoccus* * Croceicoccus bisphenolivorans* * Croceicoccus gelatinilyticus* * Croceicoccus hydrothermalis* * Croceicoccus marinus* * Croceicoccus mobilis* * Croceicoccus naphthovorans* * Croceicoccus pelagius* * Croceicoccus ponticola* * Croceicoccus sediminis*
	* Erythrobacter Erythrobacter ani Erythrobacter aurantius Erythrobacter colymbi Erythrobacter crassostreae Erythrobacter cryptus Erythrobacter dokdonensis Erythrobacter donghaensis Erythrobacter insulae Erythrobacter litoralis Erythrobacter longus Erythrobacter neustonensis Erythrobacter ramosus Erythrobacter rubeus Erythrobacter sanguineus Erythrobacter tepidarius Flavinovosphingobium* gen. nov.* Flavinovosphingobium sediminicola* comb. nov. *(Novosphingobium sediminicola) Novosphingobium Novosphingobium acidiphilum Novosphingobium aromaticivorans Novosphingobium capsulatum Novosphingobium fuchskuhlense Novosphingobium hassiacum Novosphingobium huizhouense Novosphingobium jiangmenense Novosphingobium lentum Novosphingobium meiothermophilum Novosphingobium nitrogenifigens Novosphingobium olei Novosphingobium percolationis Novosphingobium pokkalii Novosphingobium rhizosphaerae Novosphingobium subterraneum Novosphingobium taihuense*
	* Paranovosphingobium* gen. nov.* Paranovosphingobium aquiterrae* comb. nov. *(Novosphingobium aquiterrae) Paranovosphingobium arvoryzae* comb. nov. *(Novosphingobium arvoryzae) Paranovosphingobium kunmingense* comb. nov. *(Novosphingobium kunmingense) Paraurantiacibacter Paraurantiacibacter namhicola Parerythrobacter Parerythrobacter jejuensis Parerythrobacter lutipelagi Pelagerythrobacter Pelagerythrobacter aerophilus Pelagerythrobacter marensis Pelagerythrobacter marinus Pelagerythrobacter rhizovicinus Pontixanthobacter Pontixanthobacter aestiaquae Pontixanthobacter aquaemixtae Pontixanthobacter gangjinensis Pontixanthobacter luteolus Pontixanthobacter rizhaonensis Pseudoblastomonas* gen. nov.* Pseudoblastomonas halimionae* comb. nov. *(Alteriqipengyuania halimionae) Pseudoblastomonas marina* comb. nov. *(Blastomonas marina) Pseudonovosphingobium* gen. nov.* Pseudonovosphingobium aquimarinum* comb. nov. *(Novosphingobium aquimarinum) Pseudonovosphingobium aureum* comb. nov. *(Novosphingobium aureum) Pseudonovosphingobium barchaimii* comb. nov. *(Novosphingobium barchaimii) Pseudonovosphingobium chloroacetimidivorans* comb. nov. *(Novosphingobium chloroacetimidivorans) Pseudonovosphingobium clariflavum* comb. nov. *(Novosphingobium clariflavum) Pseudonovosphingobium colocasiae* comb. nov. *(Novosphingobium colocasiae) Pseudonovosphingobium decolorationis* comb. nov. *(Novosphingobium decolorationis) Pseudonovosphingobium endophyticum* comb. nov. *(Novosphingobium endophyticum) Pseudonovosphingobium fluoreni* comb. nov. *(Novosphingobium fluoreni) Pseudonovosphingobium gossypii* comb. nov. *(Novosphingobium gossypii) Pseudonovosphingobium guangzhouense* comb. nov. *(Novosphingobium guangzhouense) Pseudonovosphingobium indicum* comb. nov. *(Novosphingobium indicum) Pseudonovosphingobium lindaniclasticum* comb. nov. *(Novosphingobium lindaniclasticum) Pseudonovosphingobium malaysiense* comb. nov. *(Novosphingobium malaysiense) Pseudonovosphingobium marinum* comb. nov. *(Novosphingobium marinum) Pseudonovosphingobium mathurense* comb. nov. *(Novosphingobium mathurense) Pseudonovosphingobium naphthalenivorans* comb. nov. *(Novosphingobium naphthalenivorans) Pseudonovosphingobium panipatense* comb. nov. *(Novosphingobium panipatense) Pseudonovosphingobium pentaromativorans* comb. nov. *(Novosphingobium pentaromativorans) Pseudonovosphingobium resinovorum* comb. nov. *(Novosphingobium resinovorum) Pseudonovosphingobium silvae* comb. nov. *(Novosphingobium silvae) Pseudonovosphingobium soli* comb. nov. *(Novosphingobium soli) Pseudopontixanthobacter Pseudopontixanthobacter muriae* comb. nov. *(Alteripontixanthobacter muriae) Pseudopontixanthobacter vadosimaris*
	* Qipengyuania Qipengyuania aerophila Qipengyuania aestuarii Qipengyuania algicida Qipengyuania aquimaris Qipengyuania atrilutea* comb. nov. *(Actirhodobacter atriluteus) Qipengyuania aurantiaca Qipengyuania citrea Qipengyuania flava Qipengyuania gaetbuli Qipengyuania gelatinilytica Qipengyuania huizhouensis Qipengyuania intermedia Qipengyuania lutea* nom. nov. *(Parapontixanthobacter aurantiacus) Qipengyuania marisflavi Qipengyuania mesophila Qipengyuania nanhaisediminis Qipengyuania oceanensis Qipengyuania pacifica Qipengyuania pelagi Qipengyuania polymorpha Qipengyuania proteolytica Qipengyuania psychrotolerans Qipengyuania qiaonensis Qipengyuania sediminis Qipengyuania seohaensis Qipengyuania soli Qipengyuania sphaerica Qipengyuania vesicularis Qipengyuania vulgaris Qipengyuania xiapuensis Tsuneonella Tsuneonella aeria Tsuneonella amylolytica Tsuneonella deserti Tsuneonella dongtanensis Tsuneonella flava Tsuneonella mangrovi Tsuneonella rigui Tsuneonella suprasediminis Tsuneonella troitsensis*

### AAI and ED values distinguishing families and genera

**Fig. 2.**
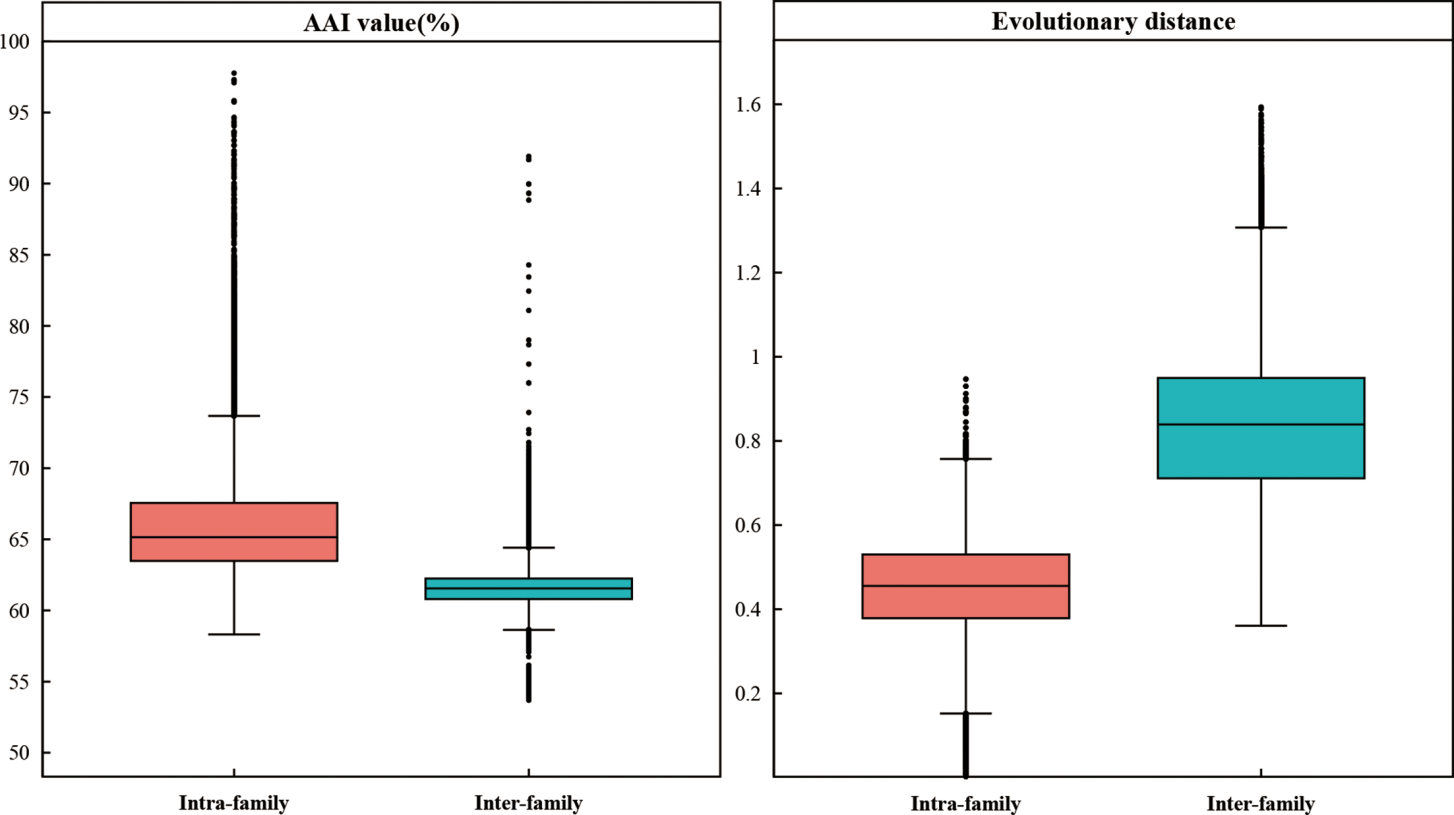
Boxplots of AAI values and EDs between intra- and inter-family proposed in this study. Red and blue indicated intra- and inter-family, respectively.

Based on recent studies, it was proposed that the taxonomic boundaries could be distinguished by using AAI and ED values [5,65]. In addition to clades 1 and 4 containing one species, the average and median intra-family AAI values of the other 11 clades ranged from 0.62 to 0.84 and 0.59 to 0.82, respectively (Table S5), while inter-family ones were 0.51 to 0.60. Inferred from the maximum-likelihood phylogenomic tree based on the gene set bac120, the average and median intra-family ED values were 0.16 to 0.57 and 0.10 to 0.60, respectively (Table S6) and inter-family ones ranged from 0.50 to 1.22. Comparisons of AAI and ED values calculated by using genomic and phylogenetic analyses supported that those 13 families were significantly separated with *p* values **<** 2.2×10^−16^ (Fig. 2). Thus, it was speculated that the AAI and ED thresholds for distinguishing different families were <0.6 and >0.5, respectively.

According to the previous genus AAI and ED boundaries of 0.7 and 0.4 applied in the family *Erythrobacteraceae* [5] and monophyletic relationship, we, therefore, reclassified 163 species into new genera with their phylogenetic topologies. Detailed reclassifications were described as follows: (1) *Sandaracinobacteroides saxicola* was reclassified into a novel genus with it as the type species; (2) three *Polymorphobacter* species were reclassified into the genus *Sandarakinorhabdus*; (3) four *Chioneia* species and two *Polymorphobacter* species were reclassified into the genus *Glacieibacterium*; (4) one *Sphingomonas* species and three *Sphingosinicella* species were reclassified into the genus *Allosphingosinicella*; (5) 16 *Sphingomonas* species were reclassified into a novel genus with *Sphingomonas jaspsi* as the type species; (6) *Sphingomonas formosensis* was reclassified into a novel genus with it as the type species; (7) two *Sphingomonas* species were reclassified into a novel genus with *Sphingomonas haloaromaticamans* as the type species; (8) four *Sphingomonas* species were reclassified into a novel genus with *Sphingomonas oligoaromativorans* as the type species; (9) two *Sphingomonas* species were classified into the genus *Rhizorhabdus*; (10) two *Sphingomonas* species were reclassified into a novel genus with *Sphingomonas solaris* as the type species; (11) four *Sphingomonas* species were reclassified into a novel genus with *Sphingomonas arantia* as the type species; (12) four *Sphingomonas* species were reclassified into a novel genus with *Sphingomonas vulcanisoli* as the type species; (13) one *Sphingobium* and two *Sphingomonas* species were reclassified into a novel genus with *Sphingobium boeckii* as the type species; (14) two *Sphingomonas* species were reclassified into a novel genus with *Sphingomonas sanxanigenens* as the type species; (15) three *Sphingomonas* species were reclassified into a novel genus with *Sphingomonas changbaiensis* as the type species; (16) *Sphingomonas gilva* was reclassified into a novel genus with it as the type species; (17) three *Sphingomonas* species were reclassified into a novel genus with *Sphingomonas guangdongensis* as the type species; (18) three *Sphingomonas* species were reclassified into a novel genus with *Sphingomonas panni* as the type species; (19) five *Sphingomonas* species were reclassified into a novel genus with *Sphingomonas japonica* as the type species; (20) two *Sphingosinithalassobacter* species were reclassified into the genus *Stakelama*; (21) 16 *Sphingomonas* species were reclassified into a novel genus with *Sphingomonas trueperi* as the type species; (22) 11 *Sphingomonas* species were reclassified into a novel genus with *Sphingomonas echinoides* as the type species; (23) five *Sphingomonas* species were reclassified into a novel genus with *Sphingomonas pruni* as the type species; (24) *Sphingomonas paeninsulae* was reclassified into a novel genus with it as the type species; (25) *Aquisediminimonas sediminicola* was reclassified into a novel genus with it as the type species; (26) *Chakrabartia godavariana* was reclassified into the genus *Aquisediminimonas*; (27) three *Sphingobium* species were reclassified into a novel genus with *Sphingobium sufflavum* as the type species; (28) three *Sphingobium* species were reclassified into a novel genus with *Sphingobium jiangsuense* as the type species; (29) *Sphingorhabdus soli* was reclassified into a novel genus with it as the type species; (30) *Sphingomonas lacunae* was reclassified into a novel genus with it as the type species; (31) *Sphingorhabdus lutea* was reclassified into a novel genus with it as the type species; (32) *Novosphingobium ipomoeae* was reclassified into a novel genus with it as the type species; (33) *Novosphingobium sediminicola* was reclassified into a novel genus with it as the type species; (34) three *Novosphingobium* species were reclassified into a novel genus with *Novosphingobium kunmingense* as the type species; (35) eight *Novosphingobium* species were reclassified into a novel genus with *Novosphingobium rosa* as the type species; (37) twenty-two *Novosphingobium* species were reclassified into a novel genus with *Novosphingobium pentaromativorans* as the type species; (38) one *Altererythrobacter* species and one *Croceicoccus* species were reclassified into the genus *Caenibius*; (39) two *Altererythrobacter* species were reclassified into the genus *Alteraurantiacibacter*; (40) one *Alteriqipengyuania* species and one *Blastomonas* species were reclassified into a novel genus with *Blastomonas marina* as the type species; (41) *Alteripontixanthobacter muriae* was reclassified into the genus *Pseudopontixanthobacter*; (42) one *Altererythrobacter* species and two *Pontixanthobacter* species were reclassified into a novel genus; (43) *Actirhodobacter atriluteus* and *Parapontixanthobacter aurantiacus* were reclassified into the genus *Qipengyuania*. In addition, based on the phylogenomic analysis and overall genome relatedness index calculations, *Chioneia frigida* and *Parapontixanthobacter aurantiacus* were reclassified into the genera *Glacieibacterium* and *Qipengyuania*, respectively. Because the names *Glacieibacterium frigidum* and *Qipengyuania aurantiaca* had been validly published [10,73], two novel names including *Glacieibacterium frigoris* nom. nov. and *Qipengyuania lutea* nom. nov. are proposed for *Chioneia frigida* and *Parapontixanthobacter aurantiacus*.

## TAXONOMIC CONCLUSIONS

### Reclassification of *Qipengyuania aerophila* as a later heterotypic synonym of *Qipengyuania pacifica*

Overall genome relatedness index calculations indicated that *Qipengyuania aerophila* and *Qipengyuania pacifica* type strain genomes shared ANI values of >96% and dDDH values of >70%. These results suggest that *Qipengyuania aerophila* and *Qipengyuania pacifica* belong to the same species of the genus *Qipengyuania*, and thus, in this study, it is proposed that *Qipengyuania aerophila* Liu *et al*. 2022 is reclassified as a later heterotypic synonym of *Qipengyuania pacifica* Tareen *et al*. 2022.

## Description of *Aquisediminimonadaceae* fam. nov

*Aquisediminimonadaceae* (A.qui.se.di.mi.ni.mo.na.da.ce’ae. N.L. fem. n. *Aquisediminimonas*, type genus of the family; L. fem. pl. n. suff. -*aceae*, ending to denote a family; N.L. fem. pl. n. *Aquisediminimonadaceae*, the *Aquisediminimonas* family).

The family *Aquisediminimonadaceae* contains the type genus *Aquisediminimonas* as well as other genera *Alteraquisediminimonas* and *Paraquisediminimonas*. Members are Gram-stain-negative, rod-shaped bacteria. Aerobic and chemoorganoheterotrophic. Do not require NaCl for growth. The predominant ubiquinone is Q-10. The major fatty acids are unsaturated hexadecenoic acid (C_16:1_) and octadecenoic acid (C_18:1_). The major polar lipids are diphosphatidylglycerol, phosphatidylcholine, phosphatidylethanolamine, phosphatidylglycerol and sphingoglycolipid. The member of this family forms a distinct clade in phylogenomic trees. The genomic size and DNA G+C content are 3.0–4.0 Mbp and 58–64%, respectively.

## Description of *Blastomonadaceae* fam. nov

*Blastomonadaceae* (Bla.sto.mo.na.da.ce’ae. N.L. fem. n. *Blastomonas*, type genus of the family; L. fem. pl. n. suff. -*aceae*, ending to denote a family; N.L. fem. pl. n. *Blastomonadaceae*, the *Blastomonas* family).

The family *Blastomonadaceae* contains a sole type genus *Blastomonas*. Members are Gram-stain-negative, rod- or ovoid-shaped bacteria. Aerobic and chemoorganoheterotrophic. Contain bacteriochlorophyll-*a*. The member of this family forms a distinct clade in phylogenomic trees. The genomic size and DNA G+C content are 3.7–4.2 Mbp and 62–65%, respectively.

## Description of *Parasphingopyxidaceae* fam. nov

*Parasphingopyxidaceae* (Pa.ra.sphin.go.py.xi.da.ce’ae. N.L. fem. n. *Parasphingopyxis*, type genus of the family; L. fem. pl. n. suff. -*aceae*, ending to denote a family; N.L. fem. pl. n. *Parasphingopyxidaceae*, the *Parasphingopyxis* family).

The family *Parasphingopyxidaceae* contains a sole type genus *Parasphingopyxis*. Members are Gram-stain-negative, rod-shaped bacteria. Aerobic and chemoorganoheterotrophic. The member of this family forms a distinct clade in phylogenomic trees. The genomic size and DNA G+C content are 2.9–3.7 Mbp and 60–64%, respectively.

## Description of *Pedomonadaceae* fam. nov

*Pedomonadaceae* (Pe.do.mo.na.da.ce’ae. N.L. fem. n. *Pedomonas*, type genus of the family; L. fem. pl. n. suff. -*aceae*, ending to denote a family; N.L. fem. pl. n. *Pedomonadaceae*, the *Pedomonas* family).

The family *Pedomonadaceae* contains a sole type genus *Pedomonas*. Members are Gram-stain-negative, rod-shaped bacteria. Aerobic and chemoorganoheterotrophic. The member of this family forms a distinct clade in phylogenomic trees. The genomic size and DNA G+C content are 3.6–3.7 Mbp and 64–65%, respectively.

## Description of *Rhizorhabdaceae* fam. nov

*Rhizorhabdaceae* (Rhi.zo.rhab.da.ce’ae. N.L. fem. n. *Rhizorhabdus*, type genus of the family; L. fem. pl. n. suff. -*aceae*, ending to denote a family; N.L. fem. pl. n. *Rhizorhabdaceae*, the *Rhizorhabdus* family).

The family *Rhizorhabdaceae* contains the type genus *Rhizorhabdus* as well as other genera *Alterirhizorhabdus*, *Edaphosphingomonas*, *Flavisphingomonas*, *Neorhizorhabdus*, *Pararhizorhabdus* and *Solisphingomonas*. Members are Gram-stain-negative, rod-shaped bacteria. Aerobic and chemoorganoheterotrophic. Do not require NaCl for growth. The predominant ubiquinone is Q-10. The major fatty acid is unsaturated octadecenoic acid (C_18:1_). The major polar lipids are phosphatidylethanolamine, phosphatidylglycerol and sphingoglycolipid. The member of this family forms a distinct clade in phylogenomic trees. The genomic size and DNA G+C content are 3.2–6.9 Mbp and 63–70%, respectively.

## Description of *Sphingobiaceae* fam. nov

*Sphingobiaceae* (Sphin.go.bi.a.ce’ae. N.L. neut. n. *Sphingobium*, type genus of the family; L. fem. pl. n. suff. -*aceae*, ending to denote a family; N.L. fem. pl. n. *Sphingobiaceae*, the *Sphingobium* family).

The family *Sphingobiaceae* contains the type genus *Sphingobium* as well as other genera *Alterisphingobium*, *Parasphingobium* and *Rhizorhapis*. Members are Gram-stain-negative, curved-rod- or rod-shaped bacteria. Aerobic or facultatively anaerobic and chemoorganoheterotrophic. The predominant ubiquinone is Q-10. The major fatty acid is unsaturated octadecenoic acid (C_18:1_). The major polar lipids are diphosphatidylglycerol, phosphatidylethanolamine and sphingoglycolipid. The member of this family forms a distinct clade in phylogenomic trees. The genomic size and DNA G+C content are 3.0–6.0 Mbp and 59–66%, respectively.

## Description of *Sphingomicrobiaceae* fam. nov

*Sphingomicrobiaceae* (Sphin.go.mi.cro.bi.a.ce’ae. N.L. neut. n. *Sphingomicrobium*, type genus of the family; L. fem. pl. n. suff. -*aceae*, ending to denote a family; N.L. fem. pl. n. *Sphingomicrobiaceae*, the *Sphingomicrobium* family).

The family *Sphingomicrobiaceae* contains the type genus *Sphingomicrobium* as well as other genera *Allosphingosinicella* and *Pseudosphingomonas*. Members are Gram-stain-negative, rod-, coccoid- or ovoid-shaped bacteria. Aerobic and chemoorganoheterotrophic. The predominant ubiquinone is Q-10. The major fatty acid is unsaturated octadecenoic acid (C_18:1_). The major polar lipid is diphosphatidylglycerol. The member of this family forms a distinct clade in phylogenomic trees. The genomic size and DNA G+C content are 2.0–6.1 Mbp and 62–69%, respectively.

## Description of *Sphingopyxidaceae* fam. nov

*Sphingopyxidaceae* (Sphin.go.py.xi.da.ce’ae. N.L. fem. n. *Sphingopyxis*, type genus of the family; L. fem. pl. n. suff. -*aceae*, ending to denote a family; N.L. fem. pl. n. *Sphingopyxidaceae*, the *Sphingopyxis* family)

The family *Sphingopyxidaceae* contains the type genus *Sphingopyxis* as well as other genera *Alterisphingopyxis* and *Flavisphingopyxis*. Members are Gram-stain-negative, rod-shaped bacteria. Aerobic and chemoorganoheterotrophic. The predominant ubiquinone is Q-10. The major fatty acid is unsaturated octadecenoic acid (C_18:1_). The major polar lipids are diphosphatidylglycerol, phosphatidylcholine, phosphatidylethanolamine, phosphatidylglycerol and sphingoglycolipid. The member of this family forms a distinct clade in phylogenomic trees. The genomic size and DNA G+C content are 2.5–5.8 Mbp and 62–67%, respectively.

## Description of *Sphingorhabdaceae* fam. nov

*Sphingorhabdaceae* (Sphin.go.rhab.da.ce’ae. N.L. fem. n. *Sphingorhabdus*, type genus of the family; L. fem. pl. n. suff. -*aceae*, ending to denote a family; N.L. fem. pl. n. *Sphingorhabdaceae*, the *Sphingorhabdus* family).

The family *Sphingorhabdaceae* contains the type genus *Sphingorhabdus* as well as other genera *Alterisphingorhabdus*, *Novosphingopyxis* and *Parasphingorhabdus*. Members are Gram-stain-negative, rod-, coccoid- or ovoid-shaped bacteria. Aerobic or facultatively anaerobic and chemoorganoheterotrophic. The predominant ubiquinone is Q-10. The major fatty acid is unsaturated octadecenoic acid (C_18:1_). The major polar lipids are phosphatidylethanolamine and phosphatidylglycerol. The member of this family forms a distinct clade in phylogenomic trees. The genomic size and DNA G+C content are 2.1–3.8 Mbp and 46–64%, respectively.

## Description of *Aestuarierythrobacter* gen. nov

*Aestuarierythrobacter* (Aes.tua.ri.e.ry.thro.bac’ter. L. neut. n. *aestuarium*, tidal flat; N.L. masc. n. *Erythrobacter*, a genus name; N.L. masc. n. *Aestuarierythrobacter*, *Erythrobacter* from a tidal flat).

Cells are Gram-stain-negative, oval- or short-rod-shaped, non-spore-forming and aerobic. Non-motile. Positive and negative for oxidase. Catalase-positive. Contains carotenoid pigments but not bacteriochlorophyll *a*. Do not require NaCl for growth. The predominant ubiquinone is Q-10. The major fatty acids (>10%) are summed feature 8 (C_18:1_* ω*7*c* and/or C_18:1_* ω*6*c*), C_17:1_* ω*6*c* and summed feature 3 (C_16:1_* ω*6*c* and/or C_16:1_* ω*7*c*). The major polar lipids are phosphatidylcholine, phosphatidylethanolamine, phosphatidylglycerol and sphingoglycolipid. The genus represents a distinct branch in the family *Erythrobacteraceae* of the order *Sphingomonadales* based on the core-genomic phylogeny. The DNA G+C content is 58–62% (by genome). The type species is *Aestuarierythrobacter aquiaggeris*.

## Description of *Aestuarierythrobacter aquiaggeris* comb. nov

*Aestuarierythrobacter aquiaggeris* (a.qui.ag’ge.ris. L. fem. n. *aqua*, water; L. masc. n. *agger*, embankment, mound; N.L. gen. n. *aquiaggeris*, of water of an embankment, the place from which the type strain was isolated).

Basonym: *Altererythrobacter aquiaggeris* Jung *et al*. 2017

The description is the same as for *Altererythrobacter aquiaggeris* [37]. The type strain, KEM-3^T^ (= KCTC 52471^T^ = NBRC 112425^T^), was isolated from water of Geumgang Estuary Bank, South Korea. The DNA G+C content of the type strain is 58.8% (by genome). The GenBank accession numbers for the 16S rRNA gene and genome sequences of the type strain are KX812543 and GCA_037154015.1, respectively.

## Description of *Aestuarierythrobacter confluentis* comb. nov

*Aestuarierythrobacter confluentis* (con.flu.en’tis. L. gen. n. *confluentis*, of a meeting place of waters).

Basonym: *Altererythrobacter confluentis* Park *et al*. 2016

Homotypic synonym: *Pontixanthobacter confluentis* (Park *et al*. 2016) Xu *et al*. 2020

The description is the same as for *Pontixanthobacter confluentis* [5,74]. The type strain, KEM-4^T^ (= KCTC 52259^T^ = NBRC 112305^T^), was isolated from water collected from an estuary environment where the ocean and a river meet at Seocheon, South Korea. The DNA G+C content of the type strain is 59.1% (by genome). The GenBank accession numbers for the 16S rRNA gene and genome sequences of the type strain are KX129915 and GCA_009827615.1, respectively.

## Description of *Aestuarierythrobacter sediminis* comb. nov

*Aestuarierythrobacter sediminis* (se.di’mi.nis. L. gen. n. *sediminis*, of sediment).

Basonym: *Altererythrobacter sediminis* Kim *et al*. 2016

Homotypic synonym: *Pontixanthobacter sediminis* (Kim *et al*. 2016) Xu *et al*. 2020

The description is the same as for *Pontixanthobacter sediminis* [5,75]. The type strain, CAU1172^T^ (= KCTC 42453^T^ = NBRC 110917^T^), was isolated from a sample of lagoon sediment from along the east coast of Korea. The DNA G+C content of the type strain is 61.5% (by genome). The GenBank accession numbers for the 16S rRNA gene and genome sequences of the type strain are KP779619 and GCA_009828115.1, respectively.

## Description of *Agrinovosphingobium* gen. nov

*Agrinovosphingobium* (A.gri.no.vo.sphin.go’bi.um. L. masc. n. *ager*, a field; N.L. neut. n. *Novosphingobium*, a genus name; N.L. neut. n. *Agrinovosphingobium*, *Novosphingobium* from the field).

Cells are Gram-stain-negative, rod-shaped, non-spore-forming and aerobic. Non-motile. Catalase- and oxidase-positive. Contains carotenoid pigments but not bacteriochlorophyll *a*. Do not require NaCl for growth. The predominant ubiquinone is Q-10. The major fatty acids (>10%) are C_18:1_* ω*7*c*, C_16:0_ and summed feature 3 (C_16:1_* ω*6*c* and/or C_16:1_* ω*7*c*). The major polar lipids are phosphatidylglycerol, phosphatidylethanolamine, diphosphatidylglycerol, phosphatidylcholine, phosphatidylmonomethylethanolamine, phosphatidyldimethylethanolamine and sphingoglycolipid. The genus represents a distinct branch in the family *Erythrobacteraceae* of the order *Sphingomonadales* based on the core-genomic phylogeny. The DNA G+C content is 62–63% (by genome). The type species is *Agrinovosphingobium ipomoeae*.

## Description of *Agrinovosphingobium ipomoeae* comb. nov

*Agrinovosphingobium ipomoeae* (i.po.moe’ae. N.L. gen. n. *ipomoeae*, of *Ipomoea*, pertaining to the isolation of the type strain from water convolvulus field).

Basonym: *Novosphingobium ipomoeae* Chen *et al*. 2017

The description is the same as for *Novosphingobium ipomoeae* [76]. The type strain, Tese-5^T^ (= BCRC 80904^T^ = KCTC 42656^T^ = LMG 28838^T^), was isolated from a water sample taken from a water convolvulus (*Ipomoea aquatica*) field in the vicinity of Taitung, Eastern Taiwan, China. The DNA G+C content of the type strain is 62.1% (by genome). The GenBank accession numbers for the 16S rRNA gene sequences of the type strain are LN811085. The type strain genome accession number deposited into the Global Catalogue of Type Strain database is GCM10017100.

## Description of *Allosphingosinicella deserti* comb. nov

*Allosphingosinicella deserti* (de.ser’ti. L. gen. n. *deserti*, of a desert, the source of the type strain)

Basonym: *Sphingomonas deserti* Liu *et al*. 2019

The description is the same as for *Sphingomonas deserti* [77]. The type strain, GL-C-18^T^ (= ACCC 60076^T^ = KCTC 62411^T^), was isolated from soil sample collected at Mu Us Sandy Land, China. The DNA G+C content of the type strain is 66.0% (by genome). The GenBank accession numbers for the 16S rRNA gene and genome sequences of the type strain are MG706144 and GCA_003012735.1, respectively.

## Description of *Allosphingosinicella flava* comb. nov

*Allosphingosinicella flava* (fla’va. L. fem. adj. *flava*, yellow, referring to the colony colour).

Basonym: *Sphingosinicella flava* Chhetri *et al*. 2021

The description is the same as for *Sphingosinicella flava* [78]. The type strain, UDD2^T^ (= KCTC 82357^T^ = NBRC 114507^T^), was isolated from maize field soil. The DNA G+C content of the type strain is 63.7% (by genome). The GenBank accession numbers for the 16S rRNA gene and genome sequences of the type strain are MN493722 and GCA_016025255.1, respectively.

## Description of *Allosphingosinicella ginsenosidimutans* comb. Nov

*Allosphingosinicella ginsenosidimutans* (gin.se.no.si.di.mu’tans. N.L. neut. n. *ginsenosidum*, ginsenoside; L. pres. part. *mutans*, transforming, converting; N.L. fem. part. adj. *ginsenosidimutans*, ginsenoside-converting).

Basonym: *Sphingosinicella ginsenosidimutans* Kim *et al*. 2019

The description is the same as for *Sphingosinicella ginsenosidimutans* [79]. The type strain, BS11^T^ (= JCM 18201^T^ = KACC 16619^T^), was isolated from the compost of Yesan province, South Korea. The DNA G+C content of the type strain is 68.0% (by genome). The GenBank accession numbers for the 16S rRNA gene and genome sequences of the type strain are JQ349043 and GCA_007995055.1, respectively.

## Description of *Allosphingosinicella humi* comb. nov

*Allosphingosinicella humi* (hu’mi. L. gen. n. *humi*, of soil).

Basonym: *Sphingosinicella humi* Qiao *et al*. 2019

The description is the same as for *Sphingosinicella humi* [80]. The type strain, QZX222^T^ (= CCTCC AB 2018030^T^ = KCTC 62519^T^), was isolated from arsenic-contained farmland soil sampled at Jiaotian village, Daye city, Hubei province, China. The DNA G+C content of the type strain is 65.9% (by genome).

## Description of *Alteraquisediminimonas* gen. nov

*Alteraquisediminimonas* (Al.ter.a.qui.se.di.mi.ni.mo’nas. L. masc. adj. *alter* another, other, different; N.L. fem. n. *Aquisediminimonas*, a genus name; N.L. fem. n. *Alteraquisediminimonas*, another or different *Aquisediminimonas*).

Cells are Gram-stain-negative, rod-shaped, non-spore-forming and aerobic. Non-motile. Negative for catalase. Oxidase-positive. Contains carotenoid pigments but not bacteriochlorophyll *a*. Do not require NaCl for growth. The predominant ubiquinone is Q-10. The major fatty acids (>10%) are summed feature 3 (C_16:1_*ω*7*c* and/or C_16:1_* ω*6*c*), summed feature 8 (C_18:1_*ω*7*c* and/or C_18:1_* ω*6*c*), C_14:0_ 2-OH and C_16:0_. The major polar lipids are diphosphatidylglycerol, phosphatidylethanolamine, phosphatidylglycerol, sphingoglycolipid and phosphatidylcholine. The genus represents a distinct branch in the family *Aquisediminimonadaceae* of the order *Sphingomonadales* based on the core-genomic phylogeny. The DNA G+C content is 58–59% (by genome). The type species is *Alteraquisediminimonas sediminicola*.

## Description of *Alteraquisediminimonas sediminicola* comb. nov

*Alteraquisediminimonas sediminicola* (se.di.mi.ni’co.la. L. neut. n. *sedimen*, sediment; L. masc./fem. n. suff. -*cola*, inhabitant, dweller; N.L. fem. n. *sediminicola*, sediment dweller).

Basonym: *Aquisediminimonas sediminicola* Jin *et al*. 2019

The description is the same as for *Aquisediminimonas sediminicola* [81]. The type strain, CH68-4^T^ (= CCTCC AB 2018062^T^ = KCTC 62205^T^), was isolated from approximately 70 cm deep within sediment collected from Daechung Reservoir, Republic of Korea. The DNA G+C content of the type strain is 58.8% (by genome). The GenBank accession numbers for the 16S rRNA gene and genome sequences of the type strain are MF782365 and GCA_019740335.1, respectively.

## Description of *Alteraurantiacibacter lauratis* comb. nov

*Alteraurantiacibacter lauratis* (lau.ra’tis. N.L. masc. n. *lauras*, laurate; N.L. gen. n. *lauratis*, of laurate, because it oxidizes laurate).

Basonym: *Altererythrobacter lauratis* Yuan *et al*. 2017

The description is the same as for *Altererythrobacter lauratis* [39]. The type strain, YIM 75003^T^ (= CCTCC AB 2016268^T^ = DSM 104093^T^ = KCTC 52606^T^), was isolated from the hot spring of Tagejia geothermal field in Tibet, western China. The DNA G+C content of the type strain is 64.5% (by genome). The GenBank accession numbers for the 16S rRNA gene and genome sequences of the type strain are KX808673 and GCA_037154155.1, respectively.

## Description of *Alteraurantiacibacter palmitatis* comb. nov

*Alteraurantiacibacter palmitatis* (pal.mi.ta’tis. N.L. masc. n. *palmitas*, palmitate; N.L. gen. n. *palmitatis*, of palmitate, because it oxidises palmitate).

Basonym: *Altererythrobacter palmitatis* Yuan *et al*. 2017

The description is the same as for *Altererythrobacter palmitatis* [39]. The type strain, YIM 75004^T^ (= CCTCC AB 2016270^T^ = DSM 104092^T^ = KCTC 52607^T^), was isolated from the hot spring of Tagejia geothermal field in Tibet, western China. The DNA G+C content of the type strain is 64.4% (by genome). The GenBank accession numbers for the 16S rRNA gene and genome sequences of the type strain are KX808674 and GCA_037154115.1, respectively.

## Description of *Alterinovosphingobium* gen. nov

*Alterinovosphingobium* (Al.te.ri.no.vo.sphin.go’bi.um. L. masc. adj. *alter*, another, other, different; N.L. neut. n. *Novosphingobium*, a genus name; N.L. neut. n. *Alterinovosphingobium*, another or different *Novosphingobium*).

Cells are Gram-stain-negative, rod-shaped, non-spore-forming and aerobic or facultatively anaerobic. Several species are motile. Positive or negative for catalase. Positive or negative for oxidase. Contains carotenoid pigments but not bacteriochlorophyll *a*. Do not require NaCl for growth. The predominant ubiquinone is Q-10. The major fatty acids (>10%) are summed feature 8 (C_18:1_*ω*7*c* and/or C_18:1_*ω*6*c*) and summed feature 3 (C_16:1_*ω*7*c* and/or C_16:1_*ω*6*c*). The major polar lipids are phosphatidylethanolamine, phosphatidylglycerol and sphingoglycolipid. The genus represents a distinct branch in the family *Erythrobacteraceae* of the order *Sphingomonadales* based on the core-genomic phylogeny. The DNA G+C content is 61–69% (by genome). The type species is *Alterinovosphingobium rosa*.

## Description of *Alterinovosphingobium aerophilum* comb. nov

*Alterinovosphingobium aerophilum* (ae.ro’phi.lum. Gr. masc. n. *aêr*, air; N.L. masc. adj. suff.-*philus*, loving; from Gr. masc. adj. *philos*, loving; N.L. neut. adj. *aerophilum*, air-loving).

Basonym: *Novosphingobium aerophilum* Liu *et al*. 2021

The description is the same as for *Novosphingobium aerophilum* [82]. The type strain, 4Y4^T^ (= GDMCC 1.1828^T^ = KACC 21946^T^), was isolated from an aquaculture farm for fish (*Ophiocephalus argus* and *Ictalurus punctatus*) culture in Jiangmen city, Guangdong province, China. The DNA G+C content of the type strain is 67.6% (by genome).

## Description of *Alterinovosphingobium bradum* comb. nov

*Alterinovosphingobium bradum* (bra’dum. N.L. neut. adj. *bradum*, slow, referring to the slow growth of this bacterium; from Gr. masc. adj. *bradys*, slow).

Basonym: *Novosphingobium bradum* Sheu *et al*. 2016

The description is the same as for *Novosphingobium bradum* [83]. The type strain, STM-24^T^ (= BCRC 80925^T^ = KCTC 42984^T^ = LMG 29291^T^), was isolated from a spring in the vicinity of Miaoli County, Taiwan, China. The DNA G+C content of the type strain is 68.7% (by genome). The GenBank accession numbers for the 16S rRNA gene sequences of the type strain are LN890294. The type strain genome accession number deposited into the Global Catalogue of Type Strain database is GCM10019133.

## Description of *Alterinovosphingobium flavum* comb. nov

*Alterinovosphingobium flavum* (fla’vum. L. neut. adj. *flavum*, yellow, referring to the colour of the colonies).

Basonym: *Novosphingobium flavum* Nguyen *et al*. 2016

The description is the same as for *Novosphingobium flavum* [84]. The type strain, UCM 28^T^ (= KACC 18571^T^ = NBRC 111647^T^), was isolated from forest soil in Gyeonggi-Do, South Korea. The DNA G+C content of the type strain is 66.7% (by genome). The GenBank accession numbers for the 16S rRNA gene and genome sequences of the type strain are KT750339 and GCA_014230315.1, respectively.

## Description of *Alterinovosphingobium humi* comb. nov

*Alterinovosphingobium humi* (hu’mi. L. gen. n. *humi*, of earth, soil, from where the type strain was isolated).

Basonym: *Novosphingobium humi* Hyeon *et al*. 2017

The description is the same as for *Novosphingobium humi* [85]. The type strain, R1-4^T^ (= JCM 31879^T^ = KACC 19094^T^), was isolated from soil from a military shooting range in the Republic of Korea. The DNA G+C content of the type strain is 63.6% (by genome). The GenBank accession numbers for the 16S rRNA gene and genome sequences of the type strain are KY658458 and GCA_028607105.1, respectively.

## Description of *Alterinovosphingobium ovatum* comb. nov

*Alterinovosphingobium ovatum* (o.va’tum. L. part. adj. *ovatum*, egg shaped, ovate, referring to the shape of this bacterium as oval-shaped).

Basonym: *Novosphingobium ovatum* Chen *et al*. 2020

The description is the same as for *Novosphingobium ovatum* [86]. The type strain, FSY-8^T^ (= BCRC 81051^T^ = KCTC 52812^T^ = LMG 30053^T^), was isolated from a freshwater mesocosm, Taichung County, Taiwan, China. The DNA G+C content of the type strain is 64.8% (by genome).

## Description of *Alterinovosphingobium piscinae* comb. nov

*Alterinovosphingobium piscinae* (pis.ci’nae. L. gen. n. *piscinae*, of a fish-pond).

Basonym: *Novosphingobium piscinae* Sheu *et al*. 2016

The description is the same as for *Novosphingobium piscinae* [87]. The type strain, SLH-16^T^ (= BCRC 80888^T^ = KCTC 42194^T^ = LMG 28418^T^), was isolated from a fish culture pond in the Jiali District, Tainan County, Taiwan, China. The DNA G+C content of the type strain is 68.8% (by genome). The GenBank accession numbers for the 16S rRNA gene and genome sequences of the type strain are LK056647 and GCA_014230355.1, respectively.

## Description of *Alterinovosphingobium rosa* comb. Nov

*Alterinovosphingobium rosa* (ro’sa. L. fem. n. *rosa*, the rose, the source of the organism).

Basonym: *Sphingomonas rosa* Takeuchi *et al*. 1995

Homotypic synonym: *Novosphingobium rosa* (Takeuchi *et al*. 1995) Takeuchi *et al*. 2001

The description is the same as for *Novosphingobium rosa* [88,89]. The type strain, NCPPB 2661^T^ (= ATCC 51837^T^ = DSM 7285^T^ = HAMBI 2068^T^ = IAM 14222^T^ = JCM 31909^T^ = LMG 17328^T^ = NBRC 15208^T^), was isolated from the hairy roots of *Rosa* sp. (rose). The DNA G+C content of the type strain is 64.5% (by genome). The GenBank accession numbers for the 16S rRNA gene and genome sequences of the type strain are AB680810 and GCA_001598555.1, respectively.

## Description of *Alterinovosphingobium umbonatum* comb. nov

*Alterinovosphingobium umbonatum* (um.bo.na’tum. N.L. neut. adj. *umbonatum*, bossed, umbonate, referring to the umbonate elevations of outgrown colonies on solid culture media; from L. masc. n. *umbo* (gen. *umbonis*), a shield boss).

Basonym: *Novosphingobium umbonatum* Sheu *et al*. 2020

The description is the same as for *Novosphingobium umbonatum* [90]. The type strain, FSY-9^T^ (= BCRC 81052^T^ = KCTC 52813^T^ = LMG 30054^T^), was isolated from a freshwater mesocosm, Taichung County, Taiwan, China. The DNA G+C content of the type strain is 61.5% (by genome).

## Description of *Alterirhizorhabdus* gen. nov

*Alterirhizorhabdus* (Al.te.ri.rhi.zo.rhab’dus. L. masc. adj. *alter*, another, other, different; N.L. fem. n. *Rhizorhabdus*, a genus name; N.L. fem. n. *Alterirhizorhabdus*, another or different *Rhizorhabdus*)

Cells are Gram-stain-negative, rod-shaped, non-spore-forming and aerobic. Motile. Positive for catalase. Positive for oxidase. Contains carotenoid pigments but not bacteriochlorophyll *a*. Do not require NaCl for growth. The predominant ubiquinone is Q-10. The major fatty acids (>10%) are summed feature 8 (C_18:1_ *ω*7*c* and/or C_18:1_ *ω*6*c*) and C_14:0_ 2-OH. The major polar lipids are phosphatidylethanolamine, sphingoglycolipid, phosphatidylglycerol, phosphatidylcholine and diphosphatidylglycerol. The genus represents a distinct branch in the family *Rhizorhabdaceae* of the order *Sphingomonadales* based on the core-genomic phylogeny. The DNA G+C content is 67–70% (by genome). The type species is *Alterirhizorhabdus solaris*.

## Description of *Alterirhizorhabdus profundi* comb. nov

*Alterirhizorhabdus profundi* (pro.fun’di. L. gen. n. *profundi*, of the depth of the sea).

Basonym: *Sphingomonas profundi* Yang *et al*. 2020

The description is the same as for *Sphingomonas profundi* [91]. The type strain, LMO-1^T^ (= JCM 33666^T^ = MCCC 1K04066^T^), was isolated from marine sediment collected from the Mariana Trench in the western Pacific Ocean. The DNA G+C content of the type strain is 69.2% (by genome).

## Description of *Alterirhizorhabdus solaris* comb. nov

*Alterirhizorhabdus solaris* (so.la’ris. L. fem. adj. *solaris*, pertaining to the sun, referring to the origin of the type strain, isolated from the surface of solar panels)

Basonym: *Sphingomonas solaris* Tanner *et al*. 2020

The description is the same as for *Sphingomonas solaris* [92]. The type strain, R4DWN^T^ (= CECT 9811^T^ = LMG 31344^T^), was isolated from the surface of a solar panel in Boston, MA, USA. The DNA G+C content of the type strain is 67.9% (by genome). The GenBank accession numbers for the 16S rRNA gene and genome sequences of the type strain are MK569518 and GCA_007785815.1, respectively.

## Description of *Alterisphingobium* gen. nov

*Alterisphingobium* (Al.te.ri.sphin.go’bi.um. L. masc. adj. *alter*, another, other, different; N.L. neut. n. *Sphingobium*, a genus name; N.L. neut. n. *Alterisphingobium*, another or different *Sphingobium*)

Cells are Gram-stain-negative, rod-shaped, non-spore-forming and aerobic. Non-motile. Positive for catalase. Positive or negative for oxidase. Several members contain carotenoid pigments but not bacteriochlorophyll *a*. Several members require NaCl for growth. The predominant ubiquinone is Q-10. The major fatty acids (>10%) are summed feature 8 (C_18:1_*ω*6*c* and/or C_18:1_*ω*7*c*). The major polar lipids are phosphatidylethanolamine, phosphatidylglycerol, diphosphatidylglycerol and sphingoglycolipid. The genus represents a distinct branch in the family *Sphingobiaceae* of the order *Sphingomonadales* based on the core-genomic phylogeny. The DNA G+C content is 61–66% (by genome). The type species is *Alterisphingobium jiangsuense*.

## Description of *Alterisphingobium fluviale* comb. nov

*Alterisphingobium fluviale* (flu.vi.a’le. L. neut. adj. *fluviale*, belonging to a river, the source of the type strain)

Basonym: *Sphingobium fluviale* Sheu *et al*. 2020

The description is the same as for *Sphingobium fluviale* [93]. The type strain, CHR27^T^ (= BCRC 81121^T^ = KCTC 62510 ^T^ = LMG 30596^T^), was isolated from the Dahu River in Dahu Township of Miaoli County, Taiwan, China. The DNA G+C content of the type strain is 61.9% (by genome).

## Description of *Alterisphingobium jiangsuense* comb. nov

*Alterisphingobium jiangsuense* (jiang.su.en’se. N.L. neut. adj. *jiangsuense*, of or pertaining to Jiangsu, the province where the type strain was isolated).

Basonym: *Sphingobium jiangsuense* Zhang *et al*. 2012

The description is the same as for *Sphingobium jiangsuense* [94]. The type strain, BA-3^T^ (= CCTCC AB 2010217^T^ = DSM 26189 ^T^ = KACC 16433^T^ = KCTC 23196^T^), was isolated from a wastewater treatment system in a pesticide manufacturing company in Jiangsu province, China. The DNA G+C content of the type strain is 66.0% (by genome). The GenBank accession numbers for the 16S rRNA gene and genome sequences of the type strain are HM748834 and GCA_030161155.1, respectively.

## Description of *Alterisphingobium subterraneum* comb. nov

*Alterisphingobium subterraneum* (sub.ter.ra’ne.um. L. neut. adj. *subterraneum*, underground).

Basonym: *Sphingobium subterraneum* Lee *et al*. 2015

The description is the same as for *Sphingobium subterraneum* [95]. The type strain, S-II-13^T^ (= DSM 102255^T^ = KACC 17606^T^ = NBRC 109814^T^), was isolated from ground water at Daejeon in Korea. The DNA G+C content of the type strain is 62.2% (by genome). The GenBank accession numbers for the 16S rRNA gene and genome sequences of the type strain are FJ796422 and GCA_014199435.1, respectively.

## Description of *Alterisphingomonas* gen. nov

*Alterisphingomonas* (Al.te.ri.sphin.go.mo’nas. L. masc. adj. *alter*, another, other, different; N.L. fem. n. *Sphingomonas*, a genus name; N.L. fem. n. *Alterisphingomonas*, another or different *Sphingomonas*).

Cells are Gram-stain-negative, rod-shaped, non-spore-forming and aerobic. Non-motile. Positive for catalase. Positive for oxidase. Contain carotenoid pigments but not bacteriochlorophyll *a*. Do not require NaCl for growth. The predominant ubiquinone is Q-10. The major fatty acids (>10%) are summed feature 8 (C_18:1_* ω*6*c* and/or C_18:1 _*ω*7*c*) and C_16:0_. The major polar lipids are phosphatidylethanolamine, phosphatidylglycerol, diphosphatidylglycerol and sphingoglycolipid. The genus represents a distinct branch in the family *Sphingomonadaceae* of the order *Sphingomonadales* based on the core-genomic phylogeny. The DNA G+C content is 64–66% (by genome). The type species is *Alterisphingomonas pruni*.

## Description Of *Alterisphingomonas asaccharolytica* comb. nov.

*Alterisphingomonas asaccharolytica* (a.sac.cha.ro.ly’ti.ca. Gr. pref. *a*-, not; Gr. neut. n. *sakchar*, sugar; N.L. masc. adj. *lyticus*, able to lose, able to dissolve; from Gr. masc. adj. *lytikos*, able to loosen; N.L. fem. adj. *asaccharolytica*, not digesting sugar).

Basonym: *Sphingomonas asaccharolytica* Takeuchi *et al*. 1995

The description is the same as for *Sphingomonas asaccharolytica* [89]. The type strain, Y-345^T^ (= ATCC 51839^T^ = DSM 10564^T^ = HAMBI 2139^T^ = JCM 21229^T^ = KCTC 2825^T^ = LMG 17539^T^ = NBRC 15499^T^), was isolated from the roots of *Malus* spp. (apple) in Tsukuba City, Japan. The DNA G+C content of the type strain is 64.6% (by genome). The GenBank accession numbers for the 16S rRNA gene and genome sequences of the type strain are Y09639 and GCA_001598355.1, respectively.

## Description of *Alterisphingomonas mali* comb. nov

*Alterisphingomonas mali* (ma’li. N.L. gen. n. *mali*, of *Malus*, the apple genus, the source of the organism; from L. fem. n. *malus*, an apple tree).

Basonym: *Sphingomonas mali* Takeuchi *et al*. 1995

The description is the same as for *Sphingomonas mali* [36,89]. The type strain, Y-347^T^ (= ATCC 51840^T^ = CGMCC 1.7044^T^ = DSM 10565^T^ = HAMBI 2070^T^ = JCM 10193^T^ = LMG 17331^T^ = NBRC 15500^T^), was isolated from the roots of *Malus* spp. (apple) in Tsukuba City, Japan. The DNA G+C content of the type strain is 64.9% (by genome). The GenBank accession numbers for the 16S rRNA gene and genome sequences of the type strain are AB680884 and GCA_001598415.1, respectively.

## Description of *Alterisphingomonas panacisoli* comb. nov.

*Alterisphingomonas panacisoli* (pa.na.ci.so’li. L. masc. n. *Panax*, the genus name of the ginseng plant; L. neut. n. *solum*, soil; N.L. gen. n. *panacisoli*, of ginseng soil).

Basonym: *Sphingomonas panacisoli* Maeng *et al*. 2021

The description is the same as for *Sphingomonas panacisoli* [96]. The type strain, HKS19^T^ (= KACC 18881^T^ = LMG 29564^T^), was isolated from a ginseng cultivating soil sample collected in South Korea. The DNA G+C content of the type strain is 65.1% (by genome).

## Description of *Alterisphingomonas pruni* comb. nov

*Alterisphingomonas pruni* (pru’ni. L. fem. n. *prunus*, a plum-tree, and also the genus of peach (*Prunus*); N.L. gen. n. *pruni*, of *Prunus*, intended to mean of peach (*Prunus persica*), the source of the organism).

Basonym: *Sphingomonas pruni* Takeuchi *et al*. 1995

The description is the same as for *Sphingomonas pruni* [89]. The type strain, Y-250^T^ (= ATCC 51838^T^ = DSM 10566^T^ = HAMBI 2069^T^ = KCTC 2824^T^ = LMG 18380^T^ = NBRC 15498^T^), was isolated from the roots of *Prunus persica* (peach) in Tsukuba City, Japan. The DNA G+C content of the type strain is 64.8% (by genome). The GenBank accession numbers for the 16S rRNA gene and genome sequences of the type strain are Y09637 and GCA_001598455.1, respectively.

## Description of *Alterisphingomonas radiodurans* comb. nov

*Alterisphingomonas radiodurans* (ra.di.o.du’rans. L. masc. n. *radius*, a beam or ray; L. v. *radio*, to radiate, to emit rays; L. pres. part. *durans*, enduring; N.L. part. adj. *radiodurans*, resisting radiation).

Basonym: *Sphingomonas radiodurans* Liu *et al*. 2022

The description is the same as for *Sphingomonas radiodurans* [13]. The type strain, S9-5^T^ (= GDMCC 1.2714^T^ = JCM 34750^T^), was isolated from moraine from the north slope of Mount Everest, China. The DNA G+C content of the type strain is 65.7% (by genome). The GenBank accession numbers for the 16S rRNA gene and genome sequences of the type strain are MZ314856 and GCA_020866845.1, respectively.

## Description of *Alterisphingopyxis* gen. nov

*Alterisphingopyxis* (Al.te.ri.sphin.go.py’xis. L. masc. adj. *alter*, another, other, different; N.L. fem. n. *Sphingopyxis*, a genus name; N.L. fem. n. *Alterisphingopyxis*, another or different *Sphingopyxis*).

Cells are Gram-stain-negative, rod-shaped, non-spore-forming and aerobic. Non-motile. Positive for catalase. Negative for oxidase. Contain carotenoid pigments but not bacteriochlorophyll *a*. Do not require NaCl for growth. The predominant ubiquinone is Q-10. The major fatty acids (>10%) are summed feature 8 (C_18:1_* ω*6*c* and/or C_18:1_* ω*7*c*) and C_17:1_* ω*6*c*. The major polar lipids are phosphatidylethanolamine, phosphatidylglycerol, diphosphatidylglycerol, phosphatidyldimethylethanolamine, phosphatidylcholine and sphingoglycolipid. The genus represents a distinct branch in the family *Sphingopyxidaceae* of the order *Sphingomonadales* based on the core-genomic phylogeny. The DNA G+C content is 62% (by genome). The type species is *Alterisphingopyxis lacunae*.

## Description of *Alterisphingopyxis lacunae* comb. nov

*Alterisphingopyxis lacunae* (la.cu’nae. L. gen. n. *lacunae*, of a pond, from where the type strain was isolated).

Basonym: *Sphingomonas lacunae* Sheu *et al*. 2020

The description is the same as for *Sphingomonas lacunae* [97]. The type strain, CSW-10^T^ (= BCRC 81190^T^ = LMG 31340^T^), was isolated from a freshwater pond in the Xingang National Primary School, Xingang Township in Chiayi County, Taiwan, China. The DNA G+C content of the type strain is 62.0% (by genome).

## Description of *Alterisphingorhabdus* gen. nov

*Alterisphingorhabdus* (Al.te.ri.sphin.go.rhab’dus. L. masc. adj. *alter*, another, other, different; N.L. fem. n. *Sphingorhabdus*, a genus name; N.L. fem. n. *Alterisphingorhabdus*, another or different *Sphingorhabdus*).

Cells are Gram-stain-negative, rod-shaped, non-spore-forming and aerobic. Non-motile. Positive for catalase. Positive for oxidase. Contain carotenoid pigments but not bacteriochlorophyll *a*. Do not require NaCl for growth. The predominant ubiquinone is Q-10. The major fatty acids (>10%) are summed feature 3 (C_16:1_*ω*7*c* and/or C_16:1_*ω*6*c*), summed feature 8 (C_18:1_ *ω*7*c* and/or C_18:1_*ω*6*c*) and C_14:0_ 2-OH. The major polar lipids are phosphatidylethanolamine, phosphatidylglycerol, diphosphatidylglycerol, phosphatidylcholine and sphingoglycolipid. The genus represents a distinct branch in the family *Sphingorhabdaceae* of the order *Sphingomonadales* based on the core-genomic phylogeny. The DNA G+C content is 46–47% (by genome). The type species is *Alterisphingorhabdus lutea*.

## Description of *Alterisphingorhabdus lutea* comb. nov

*Alterisphingorhabdus lutea* (lu’te.a. L. fem. adj. *lutea*, golden-yellow).

Basonym: *Sphingorhabdus lutea* Baek *et al*. 2019

The description is the same as for *Sphingorhabdus lutea* [98]. The type strain, LPB0140^T^ (= JCM 31568^T^ = KACC 18891^T^), was isolated from sea water. The DNA G+C content of the type strain is 46.1% (by genome).

## Description of *Alteristakelama* gen. nov

*Alteristakelama* (Al.te.ri.sta.ke.la’ma. L. masc. adj. *alter*, another, other, different; N.L. fem. n. *Stakelama*; N.L. fem. n. *Alteristakelama*, another or different *Stakelama*).

Cells are Gram-stain-negative, rod- or ovoid-shaped, non-spore-forming and aerobic. Several members are motile. Positive or negative for catalase. Positive or negative for oxidase. Most members contain carotenoid pigments but not bacteriochlorophyll *a*. Do not require NaCl for growth. The predominant ubiquinone is Q-10. The major fatty acids (>10%) are summed feature 8 (C_18:1_ *ω*7*c* and/or C_18:1_*ω*6*c*). The major polar lipids are phosphatidylethanolamine, phosphatidylglycerol and sphingoglycolipid. The genus represents a distinct branch in the family *Sphingomonadaceae* of the order *Sphingomonadales* based on the core-genomic phylogeny. The DNA G+C content is 64–69% (by genome). The type species is *Alteristakelama trueperi*.

## Description of *Alteristakelama azotifigens* comb. nov

*Alteristakelama azotifigens* (a.zo.ti.fi’gens. N.L. neut. n. *azotum*, nitrogen (from French noun azote); L. pres. part. *figens*, fixing, attaching; N.L. part. adj. *azotifigens*, nitrogen-fixing).

Basonym: *Sphingomonas azotifigens* Xie and Yokota 2006

The description is the same as for *Sphingomonas azotifigens* [36,99]. The type strain, Y39^T^ (= CCTCC AB 205007^T^ = CIP 109209^T^ = DSM 18530^T^ = IAM 15283^T^ = JCM 21734^T^ = NBRC 15497^T^), was isolated from paddy soil and the roots of *Oryza sativa* in Japan. The DNA G+C content of the type strain is 67.3% (by genome). The GenBank accession numbers for the 16S rRNA gene and genome sequences of the type strain are AB033947 and GCA_002091475.1, respectively.

## Description of *Alteristakelama canadensis* comb. nov

*Alteristakelama canadensis* (ca.na.den’sis. N.L. fem. adj. *canadensis*, of or pertaining to Canada).

Basonym: *Sphingomonas canadensis* Abraham *et al*. 2013

The description is the same as for *Sphingomonas canadensis* [46]. The type strain, FWC47^T^ (= CCUG 62982^T^ = DSM 29393^T^ = LMG 27141^T^), was isolated from a pulp mill sludge pond, Howe Sound, British Columbia, Canada. The DNA G+C content of the type strain is 68.3% (by genome). The GenBank accession numbers for the 16S rRNA gene and genome sequences of the type strain are HE974351 and GCA_026013525.1, respectively.

## Description of *Alteristakelama gei* comb. nov

*Alteristakelama gei* (ge’i. N.L. gen. n. *gei*, of the plant genus *Geum*)

Basonym: *Sphingomonas gei* Zhu *et al*. 2015

The description is the same as for *Sphingomonas gei* [100]. The type strain, ZFGT-11^T^ (= CCTCC AB 2013306^T^ = KCTC 32449^T^ = LMG 27608^T^), was isolated from surface-sterilized roots of *Geum aleppicum* collected from Taibai Mountain in Shaanxi Province, north-west China. The DNA G+C content of the type strain is 65.9% (by genome). The GenBank accession numbers for the 16S rRNA gene and genome sequences of the type strain are KF551181 and GCA_004792685.1, respectively.

## Description of *Alteristakelama hengshuiensis* comb. nov

*Alteristakelama hengshuiensis* (heng.shui.en’sis. N.L. fem. adj. *hengshuiensis*, of or belonging to Hengshui University in China).

Basonym: *Sphingomonas hengshuiensis* Wei *et al*. 2015

The description is the same as for *Sphingomonas hengshuiensis* [36,101]. The type strain, WHSC-8^T^ (= CCTCC AB 2015265^T^ = KCTC 42455^T^), was isolated from the soil of Hengshui Lake Wetland Reserve, Hebei province, China. The DNA G+C content of the type strain is 66.7% (by genome). The GenBank accession numbers for the 16S rRNA gene and genome sequences of the type strain are KC118516 and GCA_000935025.1, respectively.

## Description of *Alteristakelama koreensis* comb. nov

*Alteristakelama koreensis* (ko.re.en’sis. N.L. fem. adj. *koreensis*, of Korea, from where the new organisms were isolated).

Basonym: *Sphingomonas koreensis* Lee *et al*. 2001

The description is the same as for *Sphingomonas koreensis* [102]. The type strain, JSS26^T^ (= DSM 15582^T^ = JCM 11456^T^ = KCTC 2882^T^ = NBRC 16723^T^), was isolated from natural mineral water from Taejon City, Korea. The DNA G+C content of the type strain is 66.2% (by genome). The GenBank accession numbers for the 16S rRNA gene and genome sequences of the type strain are AF131296 and GCA_003953035.1, respectively.

## Description of *Alteristakelama kyeonggiensis* comb. nov

*Alteristakelama kyeonggiensis* (kye.ong.gi.en’sis. N.L. fem. adj. *kyeonggiensis*, from Kyeongg).

Basonym: *Sphingomonas kyeonggiensis* corrig. Son *et al*. 2014

The description is the same as for *Sphingomonas kyeonggiensis* [103]. The type strain, THG-DT81^T^ (= DSM 101806^T^ = JCM 18825^T^ = KACC 17173^T^), was isolated from soil of ginseng field of Pocheon province in the Republic of Korea. The DNA G+C content of the type strain is 66.8% (by genome). The GenBank accession number for the genome sequences of the type strain is GCA_014196745.1.

## Description of *Alteristakelama leidyi* comb. nov

*Alteristakelama leidyi* (lei’dy.i. N.L. gen. masc. n. *leidyi*, of Leidy, named for J. Leidy, who observed tufts of (bacterial) growth of fungi in insect guts in 1853).

Basonym: *Caulobacter leidyi* corrig. Poindexter 1964 (Approved Lists 1980)

Homotypic synonym: *Sphingomonas leidyi* (Poindexter 1964) Chen *et al*. 2012

The description is the same as for *Sphingomonas leidyi* [104,105]. The type strain, DSM 4733^T^ (= ATCC 15260^T^ = CIP 106443^T^ = VKM B-1368^T^), was isolated from a millipede hind gut. The DNA G+C content of the type strain is 67.6% (by genome). The GenBank accession numbers for the 16S rRNA gene and genome sequences of the type strain are AJ227812 and GCA_011761945.1, respectively.

## Description of *Alteristakelama naasensis* comb. nov

*Alteristakelama naasensis* (na.as.en’sis. N.L. fem. adj. *naasensis*, pertaining to NAAS, the acronym for the National Academy of Agricultural Science, where the taxonomic studies on the type strain were first performed)

Basonym: *Sphingomonas naasensis* Kim *et al*. 2014

The description is the same as for *Sphingomonas naasensis* [106]. The type strain, KIS18-15^T^ (=DSM 100060^T^ = KACC 16534^T^ = NBRC 108943^T^), was isolated from forest soil from Baengnyeong Island, South Korea. The DNA G+C content of the type strain is 67.7% (by genome). The GenBank accession numbers for the 16S rRNA gene and genome sequences of the type strain are KC735149 and GCA_011762145.1, respectively.

## Description of *Alteristakelama pituitosa* comb. nov

*Alteristakelama pituitosa* (pi.tu.i.to’sa. L. fem. adj. *pituitosa*, slimy).

Basonym: *Sphingomonas pituitosa* Denner *et al*. 2001

The description is the same as for *Sphingomonas pituitosa* [36,107]. The type strain, EDIV^T^ (= CIP 106154^T^ = DSM 13101^T^ =JCM 21727^T^ = NBRC 102491^T^), was isolated from the water of a eutrophic fountain in Vienna, Austria, in which an algal bloom was observed. The DNA G+C content of the type strain is 67.1% (by genome). The GenBank accession numbers for the 16S rRNA gene and genome sequences of the type strain are AJ243751 and GCA_001598435.1, respectively.

## Description of *Alteristakelama pokkalii* comb. nov

*Alteristakelama pokkalii* (pok.ka’li.i. N.L. gen. n. *pokkalii*, of pokkali (a variety of rice)).

Basonym: *Sphingomonas pokkalii* Menon *et al*. 2021

The description is the same as for *Sphingomonas pokkalii* [108]. The type strain, L3B27^T^ (= KCTC 42098^T^ = LMG 28266^T^ = MCC 3001^T^), was isolated from the rhizosphere of a saline-tolerant pokkali rice variety (chettivirippu) grown in Chellanam, Kerala, India. The DNA G+C content of the type strain is 66.2% (by genome). The GenBank accession numbers for the 16S rRNA gene and genome sequences of the type strain are MH251639 and GCA_003096275.1, respectively.

## Description of *Alteristakelama psychrotolerans* comb. nov

*Alteristakelama psychrotolerans* (psy.chro.to’le.rans. Gr. masc. adj. *psychros*, cold; L. pres. part. *tolerans*, tolerating; N.L. part. adj. *psychrotolerans*, tolerating cold temperature).

Basonym: *Sphingomonas psychrotolerans* Luo *et al*. 2022

The description is the same as for *Sphingomonas psychrotolerans* [109]. The type strain, Cra20^T^ (= CGMCC 1.15510^T^ = NBRC 112697^T^), was isolated from the soil sampled in the Tianshan Mountains, China. The DNA G+C content of the type strain is 65.6% (by genome). The GenBank accession numbers for the 16S rRNA gene and genome sequences of the type strain are JQ977105 and GCA_002796605.1, respectively.

## Description of *Alteristakelama soli* comb. nov

*Alteristakelama soli* (so’li. L. gen. n. *soli*, of soil, the source of the type strain).

Basonym: *Sphingomonas soli* Yang *et al*. 2006

The description is the same as for *Sphingomonas soli* [36,110]. The type strain, T5-04^T^ (= CIP 109210^T^ = DSM 18313^T^ = JCM 21668^T^ = KCTC 12210 ^T^ =NBRC 100801^T^), was isolated from soil of a ginseng field in Daejeon, South Korea. The DNA G+C content of the type strain is 65.1% (by genome). The GenBank accession numbers for the 16S rRNA gene and genome sequences of the type strain are AB166883 and GCA_001591025.1, respectively.

## Description of *Alteristakelama suaedae* comb. nov

*Alteristakelama suaedae* (su.ae’dae. N.L. gen. n. *suaedae*, of the plant *Suaeda salsa*).

Basonym: *Sphingomonas suaedae* Zhang *et al*. 2020

The description is the same as for *Sphingomonas suaedae* [111]. The type strain, XS-10^T^ (= CGMCC 1.17078^T^ = JCM 33850^T^), was isolated from rhizosphere soil of *Suaeda salsa*, in Inner Mongolia, China. The DNA G+C content of the type strain is 65.5% (by genome).

## Description of *Alteristakelama trueperi* comb. nov

*Alteristakelama trueperi* (true’per.i. N.L. gen. n. *trueperi*, of Trüper, in honour of Hans G. Trüper, a German microbiologist, in recognition of his numerous contributions to the taxonomy of the *Pseudomonadota*).

Basonym: *Sphingomonas trueperi* Kämpfer *et al*. 1997

The description is the same as for *Sphingomonas trueperi* [112]. The type strain, DSM 7225^T^ (= ATCC 12417^T^ = CIP 105252^T^ = JCM 10278^T^ = LMG 2142^T^ = NBRC 100456^T^ = NCIMB 9391^T^), was isolated from soil. The DNA G+C content of the type strain is 67.4% (by genome). The GenBank accession numbers for the 16S rRNA gene and genome sequences of the type strain are X97776 and GCA_011927635.1, respectively.

## Description of *Alteristakelama turrisvirgatae* comb. nov

*Alteristakelama turrisvirgatae* (tur.ris.vir.ga’tae. L. gen. n. *turris*, tower; L. masc. adj. *virgatus*, striped; N.L. gen. n. *turrisvirgatae*, from the name of the site where the organism was first isolated, namely Tor Vergata, Rome, Italy).

Basonym: *Sphingomonas turrisvirgatae* Thaller *et al*. 2018

The description is the same as for *Sphingomonas turrisvirgatae* [113]. The type strain, MCT13^T^ (= BAC RE RSCIC 7^T^ = DSM 105457^T^), was isolated from the water of a drainage ditch, within a disused system of constructed wetlands of the Tor Vergata University in Rome (Italy). The DNA G+C content of the type strain is 65.3% (by genome). The GenBank accession numbers for the 16S rRNA gene and genome sequences of the type strain are MG077083 and GCA_001721295.1, respectively.

## Description of *Alteristakelama xinjiangensis* comb. nov

*Alteristakelama xinjiangensis* (xin.jiang.en’sis. N.L. fem. adj. *xinjiangensis*, pertaining to Xinjiang, an autonomous region in north-west China, where the type strain was isolated)

Basonym: *Sphingomonas xinjiangensis* An *et al*. 2011

The description is the same as for *Sphingomonas xinjiangensis* [114]. The type strain, 10-1-84^T^ (= CCTCC AB 208035^T^ = CIP 110458^T^ = DSM 26736^T^ = NRRL B-51332^T^), was isolated from the surface layer of a desert soil from Xinjiang, China. The DNA G+C content of the type strain is 64.2% (by genome). The GenBank accession numbers for the 16S rRNA gene and genome sequences of the type strain are FJ754464 and GCA_014199255.1, respectively.

## Description of *Aquisediminimonas godavariana* comb. nov

*Aquisediminimonas godavariana* (go.da.va.ri.a’na. N.L. fem. adj. *godavariana*, referring to the river Godavari from which the strain was isolated).

Basonym: *Chakrabartia godavariana* Jani *et al*. 2019

The description is the same as for *Chakrabartia godavariana* [115,116]. The type strain, PRB40^T^ (= GDMCC 1.1197^T^ = KCTC 52678^T^ = LMG 29985^T^ = MCC 3406^T^), was isolated from water samples collected from Godavari river, India during the Kumbh Mela. The DNA G+C content of the type strain is 63.8% (by genome).

## Description of *Caenibius estronivorus* comb. nov

*Caenibius estronivorus* (es.tro.ni.vo’rus. N.L. neut. n. *estronum*, estrone; L. v. *voro*, to eat, to devour; N.L. masc. adj. *estronivorus*, estrone eating).

Basonym: *Altererythrobacter estronivorus* Qin *et al*. 2021

Homotypic synonym: *Croceicoccus estronivorus* (Qin *et al*. 2021) Kim *et al.* 2021

The description is the same as for *Croceicoccus estronivorus* [117,118]. The type strain, MH-B5^T^ (= CCTCC AB 2012025^T^ = DSM 25986^T^), was isolated from Yundang Lagoon in Xiamen city, Fujian, China. The DNA G+C content of the type strain is 60.0% (by genome). The GenBank accession numbers for the 16S rRNA gene and genome sequences of the type strain are JQ723700 and GCA_001625325.1, respectively.

## Description of *Caenibius fulvus* comb. nov

*Caenibius fulvus* (ful’vus. L. masc. adj. *fulvus*, deep yellow, referring to the colour of the colonies)

Basonym: *Altererythrobacter fulvus* Dahal and Kim 2018

The description is the same as for *Altererythrobacter fulvus* [38]. The type strain, S-54^T^ (= KACC 19119^T^ = KEMB 9005-542^T^ = NBRC 112676^T^), was isolated from forest soil sampled at Kyonggi University, Suwon, Republic of Korea. The DNA G+C content of the type strain is 63.8% (by genome). The GenBank accession numbers for the 16S rRNA gene and genome sequences of the type strain are KY117470 and GCA_037154085.1, respectively.

## Description of *Edaphosphingomonas* gen. nov

*Edaphosphingomonas* (E.da.pho.sphin.go.mo’nas. Gr. neut. n. *edaphos*, soil; N.L. fem. n. *Sphingomonas*, a genus name; N.L. fem. n. *Edaphosphingomonas*, *Sphingomonas* from soil).

Cells are Gram-stain-negative, rod-shaped, non-spore-forming and aerobic. Non-motile. Positive for catalase. Positive for oxidase. Several members contain carotenoid pigments but not bacteriochlorophyll *a*. Do not require NaCl for growth. The predominant ubiquinone is Q-10. The major fatty acids (>10%) are summed feature 8 (C_18:1_*ω*7*c* and/or C_18:1_*ω*6*c*), C_16:0_, summed feature 3 (C_16:1_*ω*7*c* and/or C_16:1_*ω*6*c*). The major polar lipids are phosphatidylethanolamine, phosphatidylglycerol, diphosphatidylglycerol and sphingoglycolipid. The genus represents a distinct branch in the family *Rhizorhabdaceae* of the order *Sphingomonadales* based on the core-genomic phylogeny. The DNA G+C content is 65–67% (by genome). The type species is *Edaphosphingomonas haloaromaticamans*.

## Description of *Edaphosphingomonas fennica* comb. nov

*Edaphosphingomonas fennica* (fen’ni.ca. N.L. fem. adj. *fennica*, pertaining to Finland, from where the type strain was isolated).

Basonym: *Sphingomonas fennica* Wittich *et al*. 2007

The description is the same as for *Sphingomonas fennica* [72]. The type strain, K101^T^ (= CCUG 53462^T^ = CIP 109722^T^= DSM 13665^T^), was isolated from polychlorophenol-contaminated groundwater adjacent to a sawmill in Southern Finland. The DNA G+C content of the type strain is 66.8% (by genome). The GenBank accession numbers for the 16S rRNA gene and genome sequences of the type strain are AJ009706 and GCA_003034225.1, respectively.

## Description of *Edaphosphingomonas haloaromaticamans* comb. nov

*Edaphosphingomonas haloaromaticamans* (ha.lo.a.ro.ma.tic.a’mans. N.L. neut. n. *haloaromaticum*, haloaromatic, class of chemical compound; L. pres. part. *amans*, loving; N.L. part. adj. *haloaromaticamans*, loving haloaromatics).

Basonym: *Sphingomonas haloaromaticamans* Wittich *et al*. 2007

The description is the same as for *Sphingomonas haloaromaticamans* [72]. The type strain, A175^T^ (= CCUG 53463^T^ = CIP 109723^T^ = DSM 13477^T^), was isolated from water and soil samples from the Netherlands. The DNA G+C content of the type strain is 66.6% (by genome). The GenBank accession number for the 16S rRNA gene sequences of the type strain is X94101. The type strain genome accession number deposited into the Global Catalogue of Type Strain database is GCM10027149.

## Description of *Edaphosphingomonas laterariae* comb. nov

*Edaphosphingomonas laterariae* (la.te.ra’ri.ae. L. gen. n. *laterariae*, of a brick kiln).

Basonym: *Sphingomonas laterariae* Kaur *et al*. 2012

The description is the same as for *Sphingomonas laterariae* [24,36]. The type strain, LNB2^T^ (= CCM 7880^T^ = DSM 25432^T^ = MTCC 10873^T^), was isolated from Ummari village, near Lucknow, India. The DNA G+C content of the type strain is 65.5% (by genome). The GenBank accession numbers for the 16S rRNA gene and genome sequences of the type strain are HM159118 and GCA_900188165.1, respectively.

## Description of *Flavinovosphingobium* gen. nov

*Flavinovosphingobium* (Fla.vi.no.vo.sphin.go’bi.um. L. masc. adj. *flavus*, yellow; N.L. neut. n. *Novosphingobium*, a genus name; N.L. neut. n. *Flavinovosphingobium*, yellow *Sphingobium*).

Cells are Gram-stain-negative, rod-shaped, non-spore-forming and aerobic. Non-motile. Positive for catalase. Positive for oxidase. Contain carotenoid pigments but not bacteriochlorophyll *a*. Do not require NaCl for growth. The predominant ubiquinone is Q-10. The major fatty acids (>10%) are summed feature 4 (C_16:1_*ω*7*c* and/or iso-C_15:0_ 2-OH), summed feature 7 (C_18:1_*ω*7*c*, C_18:1_*ω*9*c*, C_18:1_*ω*9*t* and/or C_18:1_*ω*12*t*) and C_16:0_. The major polar lipids are phosphatidylethanolamine, phosphatidyldimethylethanolamine, phosphatidylglycerol, sphingoglycolipid and phosphatidylcholine. The genus represents a distinct branch in the family *Erythrobacteraceae* of the order *Sphingomonadales* based on the core-genomic phylogeny. The DNA G+C content is 65–67% (by genome). The type species is *Flavinovosphingobium sediminicola*.

## Description of *Flavinovosphingobium sediminicola* comb. nov

*Flavinovosphingobium sediminicola* (se.di.mi.ni’co.la. L. neut. n. *sedimen*, sediment; L. masc./fem. n. suff. -*cola*, inhabitant, dweller; from L. masc./fem. n. *incola*, dweller; N.L. n. *sediminicola*, sediment-dweller, referring to the source of the type strain)

Basonym: *Novosphingobium sediminicola* Baek *et al*. 2011

The description is the same as for *Novosphingobium sediminicola* [119]. The type strain, HU1-AH51^T^ (= DSM 27057^T^ = KCTC 22311^T^ = LMG 24320^T^), was isolated from freshwater sediment of Lake Hakha, South Korea. The DNA G+C content of the type strain is 62.9% (by genome). The GenBank accession numbers for the 16S rRNA gene and genome sequences of the type strain are FJ177534 and GCA_014196525.1, respectively.

## Description of *Flavisphingomonas* gen. nov

*Flavisphingomonas* (Fla.vi.sphin.go.mo’nas. L. masc. adj. *flavus*, yellow; N.L. fem. n. *Sphingomonas*, a genus name; N.L. fem. n. *Flavisphingomonas*, yellow *Sphingomonas*).

Cells are Gram-stain-negative, rod-shaped, non-spore-forming and aerobic. Motile. Positive for catalase. Positive for oxidase. Contain carotenoid pigments but not bacteriochlorophyll *a*. Do not require NaCl for growth. The predominant ubiquinone is Q-10. The major fatty acids (>10%) are summed feature 8 (C_18:1_*ω*7*c* and/or C_18:1_*ω*6*c*). The major polar lipids are phosphatidylethanolamine, phosphatidyldimethylethanolamine, phosphatidylglycerol, sphingoglycolipid and phosphatidylcholine. The genus represents a distinct branch in the family *Rhizorhabdaceae* of the order *Sphingomonadales* based on the core-genomic phylogeny. The DNA G+C content is 65–66% (by genome). The type species is *Flavisphingomonas formosensis*.

## Description of *Flavisphingomonas formosensis* comb. nov

*Flavisphingomonas formosensis* (for.mo.sen’sis. N.L. fem. adj. *formosensis*, of or pertaining to Formosa (Taiwan), the beautiful island)

Basonym: *Sphingomonas formosensis* Lin *et al*. 2012

The description is the same as for *Sphingomonas formosensis* [120]. The type strain, CC-Nfb-2^T^ (= BCRC 80272^T^ = DSM 24164^T^), was isolated from agricultural soil. The DNA G+C content of the type strain is 65.2% (by genome). The GenBank accession numbers for the 16S rRNA gene and genome sequences of the type strain are HM193517 and GCA_009755815.1, respectively.

## Description of *Flavisphingopyxis* gen. nov

*Flavisphingopyxis* (Fla.vi.sphin.go.py’xis. L. masc. adj. *flavus*, yellow; N.L. fem. n. *Sphingopyxis*; N.L. fem. n. *Flavisphingopyxis*, yellow *Sphingopyxis*).

Cells are Gram-stain-negative, rod-shaped, non-spore-forming and aerobic. Non-motile. Positive for catalase. Positive for oxidase. Contain carotenoid pigments but not bacteriochlorophyll *a*. Do not require NaCl for growth. The predominant ubiquinone is Q-10. The major fatty acids (>10%) are summed feature 8 (C_18:1_*ω*7*c* and/or C_18:1_*ω*6*c*), summed feature 3 (C_16:1_*ω*7*c* and/or C_16:1_*ω*6*c*) and C_14:0_ 2-OH. The major polar lipids are diphosphatidylglycerol, phosphatidylethanolamine, phosphatidylglycerol, phosphatidylcholine and sphingoglycolipid. The genus represents a distinct branch in the family *Sphingopyxidaceae* of the order *Sphingomonadales* based on the core-genomic phylogeny. The DNA G+C content is 64–65% (by genome). The type species is *Flavisphingopyxis soli*.

## Description of *Flavisphingopyxis soli* comb. nov

*Flavisphingopyxis soli* (so’li. L. gen. n. *soli*, of soil, the source of the type strain).

Basonym: *Sphingorhabdus soli* Liu *et al*. 2020

The description is the same as for *Sphingorhabdus soli* [121]. The type strain, D-2Q-5-6^T^ (= KCTC 52311^T^ = MCCC 1A06070^T^), was isolated from a soil sample collected from the Arctic region. The DNA G+C content of the type strain is 64.5% (by genome).

## Description of *Glacieibacterium algoris* comb. nov

*Glacieibacterium algoris* (al’go.ris. L. gen. n. *algoris*, from coldness).

Basonym: *Chioneia algoris* Tahon *et al*. 2022

The description is the same as for *Chioneia algoris* [16]. The type strain, R-39594^T^ (= CECT 30377^T^ = LMG 31950^T^), was isolated from Naka Tempyo in the Syowa Oasis, East Antarctica. The DNA G+C content of the type strain is 68.2% (by genome).

## Description of *Glacieibacterium arshaanense* comb. nov

*Glacieibacterium arshaanense* (ar.shaan.en’se. N.L. neut. adj. *arshaanense*, pertaining to Arshaan Mountain, a mountain in the north of China, where the type strain was isolated).

Basonym: *Polymorphobacter arshaanensis* Phurbu *et al*. 2020

The description is the same as for *Polymorphobacter arshaanensis* [122]. The type strain, DJ1R-1^T^ (= CGMCC 1.13788^T^ = KCTC 72014^T^), was isolated from water of Dujuan Lake Da Hinggan Ling Mountain, in the Nei Monggol Autonomous Region, China. The DNA G+C content of the type strain is 65.0% (by genome).

## Description of *Glacieibacterium brumae* comb. nov

*Glacieibacterium brumae* (bru’mae. L. gen. n. *brumae*, from the winter, winter cold).

Basonym: *Chioneia brumae* Tahon *et al*. 2022

The description is the same as for *Chioneia brumae* [16]. The type strain, R-39161^T^ (= CECT 30378^T^ = LMG 31951^T^), was isolated from Naka Tempyo in the Syowa Oasis, East Antarctica. The DNA G+C content of the type strain is 68.9% (by genome).

## Description of *Glacieibacterium frigoris* nom. nov

*Glacieibacterium frigoris* (fri’go.ris. L. gen. n. *frigoris*, of the cold).

Basonym: *Chioneia frigida* Tahon *et al*. 2022

The description is the same as for *Chioneia frigida* [16]. The type strain, R-67880^T^ (= CECT 30379^T^ = LMG 31952^T^), was isolated from Utsteinen ridge, in the neighbourhood of the Belgian Princess Elisabeth Station, East Antarctica. The DNA G+C content of the type strain is 67.4% (by genome).

## Description of *Glacieibacterium hiemis* comb. nov

*Glacieibacterium hiemis* (hi’e.mis. L. gen. n. *hiemis*, of winter).

Basonym: *Chioneia hiemis* Tahon *et al*. 2022

The description is the same as for *Chioneia hiemis* [16]. The type strain, R-36677^T^ (= CECT 30380^T^ = LMG 31953^T^), was isolated from Utsteinen, in the proximity of the Princess Elisabeth Station, East Antarctica. The DNA G+C content of the type strain is 67.8% (by genome).

## Description of *Glacieibacterium megasporae* comb. nov

*Glacieibacterium megasporae* (me.ga.spo’rae. N.L. gen. n. *megasporae*, of a lichen specimen of the genus *Megaspora*, the source of the type strain).

Basonym: *Polymorphobacter megasporae* Noh *et al*. 2022

The description is the same as for *Polymorphobacter megasporae* [123]. The type strain, PAMC 29362^T^ (= JCM 34545^T^ = KCTC 82578^T^), was isolated from a lichen *Megaspora verrucosa* collected from King George Island, Antarctica. The DNA G+C content of the type strain is 65.5% (by genome). The GenBank accession numbers for the 16S rRNA gene and genome sequences of the type strain are MW507628 and GCA_018982885.2, respectively.

## Description of *Humisphingomonas* gen. nov

*Humisphingomonas* (Hu.mi.sphin.go.mo’nas. L. fem. n. *humus*, soil; N.L. fem. n. *Sphingomonas*, a genus name; N.L. fem. n. *Humisphingomonas*, *Sphingomonas* isolated from soil).

Cells are Gram-stain-negative, rod-shaped, non-spore-forming and aerobic. Motile. Negative for catalase. Negative for oxidase. Contain carotenoid pigments but not bacteriochlorophyll *a*. Do not require NaCl for growth. The predominant ubiquinone is Q-10. The major fatty acids (>10%) are summed feature 8 (C_18:1_*ω*7*c* and/or C_18:1_*ω*6*c*), summed feature 3 (C_16:1_*ω*7*c* and/or C_16:1_*ω*6*c*) and C_14:0_ 2-OH. The major polar lipids are diphosphatidylglycerol, phosphatidylethanolamine, phosphatidylglycerol, phosphatidylcholine and sphingoglycolipid. The genus represents a distinct branch in the family *Sphingomonadaceae* of the order *Sphingomonadales* based on the core-genomic phylogeny. The DNA G+C content is 67–68% (by genome). The type species is *Humisphingomonas gilva*.

## Description of *Humisphingomonas gilva* comb. nov

*Humisphingomonas gilva* (gil’va. L. fem. adj. *gilva*, yellowish, referring to the colour of the type strain)

Basonym: *Sphingomonas gilva* Zhu *et al*. 2019

The description is the same as for *Sphingomonas gilva* [124]. The type strain, ZDH117^T^ (= CCTCC AB 2018262^T^ = KCTC 62894^T^), was isolated from mountain soil sampled at Shaoguan, Guangdong Province, China. The DNA G+C content of the type strain is 67.6% (by genome). The GenBank accession numbers for the 16S rRNA gene and genome sequences of the type strain are MH892571 and GCA_003515075.1, respectively.

## Description of *Neorhizorhabdus* gen. nov.

*Neorhizorhabdus* (Ne.o.rhi.zo.rhab’dus. Gr. masc. adj. *neos*, new; N.L. fem. n. *Rhizorhabdus*, a genus name; N.L. fem. n. *Neorhizorhabdus*, new *Rhizorhabdus*)

Cells are Gram-stain-negative, rod-shaped, non-spore-forming and aerobic. Several members are motile. Positive or negative for catalase. Positive or negative for oxidase. Contain carotenoid pigments but not bacteriochlorophyll *a*. Do not require NaCl for growth. The predominant ubiquinone is Q-10. The major fatty acids (>10%) are summed feature 8 (C_18:1_*ω*7*c* and/or C_18:1_*ω*6*c*) and C_16:0_. The major polar lipids are sphingoglycolipid, phosphatidylcholine, phosphatidylglycerol and phosphatidylethanolamine. The genus represents a distinct branch in the family *Rhizorhabdaceae* of the order *Sphingomonadales* based on the core-genomic phylogeny. The DNA G+C content is 64–68% (by genome). The type species is *Neorhizorhabdus vulcanisoli*.

## Description of *Neorhizorhabdus crusticola* comb. nov

*Neorhizorhabdus crusticola* (crus.ti’co.la. L. fem. n. *crusta*, hard shell, crust; L. masc./fem. n. suff. -*cola*, a dweller, inhabitant; from L. masc./fem. n. *incola*, dweller; N.L. fem. n. *crusticola*, a dweller of crust).

Basonym: *Sphingomonas crusticola* Zhang *et al*. 2017

The description is the same as for *Sphingomonas crusticola* [125]. The type strain, MIMD3^T^ (= KCTC 42801^T^ = MCCC 1K01310^T^), was isolated from biological soil crusts collected in Liangcheng, north-western China. The DNA G+C content of the type strain is 64.7% (by genome). The GenBank accession numbers for the 16S rRNA gene and genome sequences of the type strain are KT346426 and GCA_003391115.1, respectively.

## Description of *Neorhizorhabdus naphthae* comb. nov

*Neorhizorhabdus naphthae* (naph’thae. Gr. fem. n. *naphtha*, oil; L. gen. n. *naphthae*, of oil)

Basonym: *Sphingomonas naphthae* Chaudhary and Kim 2016

The description is the same as for *Sphingomonas naphthae* [126]. The type strain, DKC-5-1^T^ (= JCM 31294^T^ = KACC 18716^T^=KEMB 9005-380^T^), was isolated from oil-contaminated soil in Gunsan, North Jeolla Province, South Korea. The DNA G+C content of the type strain is 67.3% (by genome). The GenBank accession numbers for the 16S rRNA gene and genome sequences of the type strain are KU312690 and GCA_028607085.1, respectively.

## Description of *Neorhizorhabdus oleivorans* comb. nov

*Neorhizorhabdus oleivorans* (o.le.i.vo’rans. L. neut. n. *oleum*, oil; L. inf. v. *vorare*, to devour; N.L. part. adj. *oleivorans*, oil-devouring, capable of utilizing oil (hydrocarbons))

Basonym: *Sphingomonas oleivorans* Chen *et al*. 2018

The description is the same as for *Sphingomonas oleivorans* [127]. The type strain, FW-11^T^ (= HAMBI 3659^T^ = LMG 29274^T^), was isolated from the oil-contaminated soil of Panjin in Liaoning, China. The DNA G+C content of the type strain is 65.4% (by genome). The GenBank accession numbers for the 16S rRNA gene and genome sequences of the type strain are KT855088 and GCA_003050615.1, respectively.

## Description of *Neorhizorhabdus vulcanisoli* comb. nov

*Neorhizorhabdus vulcanisoli* (vul.ca.ni.so’li. N.L. masc. n. *vulcanus*, volcano; L. neut. n. *solum*, soil; N.L. gen. n. *vulcanisoli*, of volcanic soil).

Basonym: *Sphingomonas vulcanisoli* Lee *et al*. 2015

The description is the same as for *Sphingomonas vulcanisoli* [128]. The type strain, SN6-13^T^ (= CECT 8804^T^ = KCTC 42454^T^), was isolated from soil of lava forest, Gotjawal, Jeju, Republic of Korea. The DNA G+C content of the type strain is 64.6% (by genome). The GenBank accession numbers for the 16S rRNA gene and genome sequences of the type strain are KP859572 and GCA_011761305.1, respectively.

## Description of *Neosandaracinobacteroides* gen. nov

*Neosandaracinobacteroides* (Ne.o.san.da.ra.ci.no.bac.te.ro’i.des. Gr. masc. adj. *neos*, new; N.L. neut. n. *Sandaracinobacteroides*, a genus name; N.L. neut. n. *Neosandaracinobacteroides*, new *Sandaracinobacteroides*)

Cells are Gram-stain-negative, rod-shaped, non-spore-forming and aerobic. Motile. Positive for catalase. Negative for oxidase. Contain carotenoid pigments but not bacteriochlorophyll *a*. Do not require NaCl for growth. The predominant ubiquinone is Q-10. The major fatty acids (>10%) are C_18:1_* ω*7*c*, C_14:0_ 2-OH, summed feature 3 (C_16:1_* ω*6*c* and/or C_16:1_* ω*7*c*) and C_16:0_. The major polar lipids are sphingoglycolipid, phosphatidylglycerol and phosphatidylethanolamine. The genus represents a distinct branch in the family *Sphingosinicellaceae* of the order *Sphingomonadales* based on the core-genomic phylogeny. The DNA G+C content is 67–68% (by genome). The type species is *Neosandaracinobacteroides saxicola*.

## Description of *Neosandaracinobacteroides saxicola* comb. nov

*Neosandaracinobacteroides saxicola* (sa.xi’co.la. L. neut. n. *saxum*, rock; L. masc./fem. n. suff. -*cola*, inhabitant; N.L. n. *saxicola*, rock dweller).

Basonym: *Sandaracinobacteroides saxicola* Tang *et al*. 2023

The description is the same as for *Sandaracinobacteroides saxicola* [11]. The type strain, M6^T^ (= CGMCC 1.19164^T^ = NBRC 115420^T^), was isolated from a fully weathered granitic soil sample on a rocky mountain in Changsha, Hunan province, China. The DNA G+C content of the type strain is 67.6% (by genome). The GenBank accession numbers for the 16S rRNA gene and genome sequences of the type strain are ON876070 and GCA_014117445.1, respectively.

## Description of *Novistakelama* gen. nov

*Novistakelama* (No.vi.sta.ke.la’ma. L. masc. adj. *novus*, new; N.L. fem. n. *Stakelama*, a genus name; N.L. fem. n. *Novistakelama*, new *Stakelama*).

Cells are Gram-stain-negative, rod-shaped, non-spore-forming and aerobic or microaerobic. Non-motile. Positive for catalase. Positive or negative for oxidase. Contain carotenoid pigments but not bacteriochlorophyll *a*. Do not require NaCl for growth. The predominant ubiquinone is Q-10. The major fatty acids (>10%) are C_18:1_* ω*7*c*. The major polar lipids are sphingoglycolipid, phosphatidylglycerol and phosphatidylethanolamine. The genus represents a distinct branch in the family *Sphingomonadaceae* of the order *Sphingomonadales* based on the core-genomic phylogeny. The DNA G+C content is 66–69% (by genome). The type species is *Novistakelama panni*.

## Description of *Novistakelama desiccabilis* comb. nov

*Novistakelama desiccabilis* (de.sic.ca’bi.lis. L. inf. v. *desiccare*, to dry up; L. suff. -*abilis*, adjectival suffix expressing passive qualities; N.L. fem. adj. *desiccabilis*, able to be dried, desiccable).

Basonym: *Sphingomonas desiccabilis* Reddy and Garcia-Pichel 2007

The description is the same as for *Sphingomonas desiccabilis* [129]. The type strain, CP1D^T^ (= ATCC BAA-1041^T^ = CIP 110412^T^ = DSM 16792^T^), was isolated from a biological soil crust from the Colorado Plateau, USA. The DNA G+C content of the type strain is 68.2% (by genome). The GenBank accession numbers for the 16S rRNA gene and genome sequences of the type strain are AJ871435 and GCA_004135605.1, respectively.

## Description of *Novistakelama hankookensis* comb. nov

*Novistakelama hankookensis* (han.kook.en’sis. N.L. fem. adj. *hankookensis*, of Hankook, the Korean name for South Korea from where the type strain was isolated).

Basonym: *Sphingomonas hankookensis* Yoon *et al*. 2009

The description is the same as for *Sphingomonas hankookensis* [130]. The type strain, ODN7^T^ (= CCUG 57509^T^ = DSM 23329^T^=KCTC 22579^T^), was isolated from a wastewater treatment plant in Taejon, Korea. The DNA G+C content of the type strain is 66.8% (by genome). The GenBank accession numbers for the 16S rRNA gene and genome sequences of the type strain are FJ194436 and GCA_022664465.1, respectively.

## Description of *Novistakelama panni* comb. nov

*Novistakelama panni* (pan’ni. L. gen. n. *panni*, of/from a piece of cloth, or by extension a wipe, referring to the fact that the type strain was isolated from a wipe).

Basonym: *Sphingomonas panni* Busse *et al*. 2005

The description is the same as for *Sphingomonas panni* [48]. The type strain, C52^T^ (= CIP 109155^T^ = DSM 15761^T^=LMG 21979^T^), was isolated from a wipe in the Medical Clinic for Small Animals, University of Veterinary Medicine, Vienna, Austria. The DNA G+C content of the type strain is 66.4% (by genome). The GenBank accession numbers for the 16S rRNA gene and genome sequences of the type strain are AJ575818 and GCA_037154175.1, respectively.

## Description of *Paranovosphingobium* gen. nov

*Paranovosphingobium* (Pa.ra.no.vo.sphin.go’bi.um. Gr. prep. *para*, next to; N.L. neut. n. *Novosphingobium*, a genus name; N.L. neut. n. *Paranovosphingobium*, a genus next to *Novosphingobium*).

Cells are Gram-stain-negative, rod-shaped, non-spore-forming and aerobic. Non-motile. Positive for catalase. Positive or negative for oxidase. Contain carotenoid pigments but not bacteriochlorophyll *a*. Do not require NaCl for growth. The predominant ubiquinone is Q-10. The major fatty acids (>10%) are summed feature 8 (C_18:1_*ω*7*c* and/or C_18:1_*ω*6*c*). The major polar lipids are phosphatidylethanolamine, phosphatidylglycerol, phosphatidylcholine, diphosphatidylglycerol and sphingoglycolipid. The genus represents a distinct branch in the family *Erythrobacteraceae* of the order *Sphingomonadales* based on the core-genomic phylogeny. The DNA G+C content is 64–66% (by genome). The type species is *Paranovosphingobium kunmingense*.

## Description of *Paranovosphingobium aquiterrae* comb. nov

*Paranovosphingobium aquiterrae* (a.qui.ter’rae. L. fem. n. *aqua*, water; L. fem. n. *terra*, earth or ground; N.L. gen. n. *aquiterrae*, from/of groundwater).

Basonym: *Novosphingobium aquiterrae* Lee *et al*. 2014

The description is the same as for *Novosphingobium aquiterrae* [131]. The type strain, E-II-3^T^ (= KACC 17599^T^ = NBRC 109812^T^ = NCAIM B.02537^T^), was isolated from ground water at Daejeon in Korea. The DNA G+C content of the type strain is 64.7% (by genome). The GenBank accession number for the 16S rRNA gene sequences of the type strain is FJ772064. The type strain genome accession number deposited into the Global Catalogue of Type Strain database is GCM10007080.

## Description of *Paranovosphingobium arvoryzae* comb. nov

*Paranovosphingobium arvoryzae* (arv.o.ry’zae. L. neut. n. *arvum*, an arable field, cultivated land; L. fem. n. *oryza*, rice; N.L. gen. n. *arvoryzae*, of a rice paddy field).

Basonym: *Novosphingobium arvoryzae* Sheu *et al*. 2018

The description is the same as for *Novosphingobium arvoryzae* [132]. The type strain, Jyi-02^T^ (= BCRC 80537^T^ = KCTC 32422^T^), was isolated from the water of a flooded rice field in the vicinity of Chiayi city, Taiwan, China. The DNA G+C content of the type strain is 65.4% (by genome). The GenBank accession numbers for the 16S rRNA gene and genome sequences of the type strain are HF548596 and GCA_014652615.1, respectively.

## Description of *Paranovosphingobium kunmingense* comb. nov

*Paranovosphingobium kunmingense* (kun.ming.en’se. N.L. neut. adj. *kunmingense*, pertaining to Kunming, a region in Yunnan, China, where the type strain was isolated)

Basonym: *Novosphingobium kunmingense* Xie *et al*. 2014

The description is the same as for *Novosphingobium kunmingense* [133]. The type strain, 18-11HK^T^ (= CGMCC 1.12274^T^ = DSM 25975^T^), was isolated from phosphate rock powder samples collected at a phosphate-mining field in Kunming, Yunnan province, China. The DNA G+C content of the type strain is 65.7% (by genome). The GenBank accession numbers for the 16S rRNA gene and genome sequences of the type strain are JQ246446 and GCA_002813245.1, respectively.

## Description of *Paraquisediminimonas* gen. nov

*Paraquisediminimonas* (Par.a.qui.se.di.mi.ni.mo’nas. Gr. prep. *para*, next to; N.L. fem. n. *Aquisediminimonas*, a genus name; N.L. fem. n. *Paraquisediminimonas*, a genus next to *Aquisediminimonas*).

Cells are Gram-stain-negative, rod-shaped, non-spore-forming and aerobic. Non-motile. Positive for catalase. Positive for oxidase. Contain carotenoid pigments but not bacteriochlorophyll *a*. Do not require NaCl for growth. The predominant ubiquinone is Q-10. The major fatty acids (>10%) are summed feature 8 (C_18:1_*ω*7*c* and/or C_18:1_*ω*6*c*) and summed feature 3 (C_16:  1_*ω*7*c* and/or C_16:1_*ω*6*c*). The major polar lipids are phosphatidylethanolamine, phosphatidylglycerol, phosphatidylcholine, diphosphatidylglycerol and sphingoglycolipid. The genus represents a distinct branch in the family *Aquisediminimonadaceae* of the order *Sphingomonadales* based on the core-genomic phylogeny. The DNA G+C content is 58–59% (by genome). The type species is *Paraquisediminimonas paeninsulae*.

## Description of *Paraquisediminimonas paeninsulae* comb. nov

*Paraquisediminimonas paeninsulae* (paen.in’su.lae. L. gen. n. *paeninsulae*, of a peninsula).

Basonym: *Sphingomonas paeninsulae* Geng *et al*. 2019

The description is the same as for *Sphingomonas paeninsulae* [134]. The type strain, YZ-8^T^ (= CCTCC AB 2017137^T^ = DSM 108678^T^=LMG 31027^T^), was isolated from soil sampled at Fildes Peninsula, Antarctica. The DNA G+C content of the type strain is 58.9% (by genome).

## Description of *Pararhizorhabdus* gen. nov

*Pararhizorhabdus* (Pa.ra.rhi.zo.rhab’dus. Gr. prep. *para*, next to; N.L. fem. n. *Rhizorhabdus*, a genus name; N.L. fem. n. *Pararhizorhabdus*, a genus next to *Rhizorhabdus*)

Cells are Gram-stain-negative, rod-shaped, non-spore-forming and aerobic. Several members are motile. Positive or negative for catalase. Positive or negative for oxidase. Contain carotenoid pigments but not bacteriochlorophyll *a*. Do not require NaCl for growth. The predominant ubiquinone is Q-10. The major fatty acids (>10%) are summed feature 8 (C_18:1_*ω*7*c* and/or C_18:1_*ω*6*c*). The major polar lipids are phosphatidylethanolamine, phosphatidylglycerol and sphingoglycolipid. The genus represents a distinct branch in the family *Rhizorhabdaceae* of the order *Sphingomonadales* based on the core-genomic phylogeny. The DNA G+C content is 66–69% (by genome). The type species is *Pararhizorhabdus arantia*.

## Description of *Pararhizorhabdus arantia* comb. nov

*Pararhizorhabdus arantia* (a.ran’ti.a. N.L. fem. adj. *arantia*, orange-coloured)

Basonym: *Sphingomonas arantia* Jia *et al*. 2016

The description is the same as for *Sphingomonas arantia* [135]. The type strain, 6P^T^ (= CGMCC 1.12702^T^ = JCM 19855^T^), was isolated from soil in the permafrost region of Hoh Xil basin in China. The DNA G+C content of the type strain is 67.6% (by genome). The type strain genome accession number deposited into the Global Catalogue of Type Strain database is GCM10020237.

## Description of *Pararhizorhabdus jatrophae* comb. Nov

*Pararhizorhabdus jatrophae* (ja.tro’phae. N.L. gen. n. *jatrophae*, of *Jatropha*, isolated from leaf tissue of *Jatropha curcas* L.)

Basonym: *Sphingomonas jatrophae* Madhaiyan *et al*. 2017

The description is the same as for *Sphingomonas jatrophae* [36,136]. The type strain, S5-249^T^ (= DSM 27345^T^ = KACC 17593^T^), was isolated as an endophyte from surface-sterilized stem tissues of *Jatropha* collected from the Agrotechnology Experimental Station, Singapore. The DNA G+C content of the type strain is 68.5% (by genome). The GenBank accession numbers for the 16S rRNA gene and genome sequences of the type strain are JQ660172 and GCA_900113315.1, respectively.

## Description of *Pararhizorhabdus montana* comb. nov

*Pararhizorhabdus montana* (mon.ta’na. L. fem. adj. *montana*, belonging to a mountain).

Basonym: Sphingomonas montana Manandhar et al. 2018

The description is the same as for *Sphingomonas montana* [137]. The type strain, W16RD^T^ (= CGMCC 1.15646^T^ = DSM 103337^T^), was isolated from soil from Tangulla Mountain, China. The DNA G+C content of the type strain is 67.0% (by genome).

## Description of *Pararhizorhabdus prati* comb. nov

*Pararhizorhabdus prati* (pra’ti. L. gen. n. *prati*, of a meadow).

Basonym: *Sphingomonas prati* Manandhar *et al*. 2016

The description is the same as for *Sphingomonas prati* [138]. The type strain, W18RD^T^ (= CGMCC 1.15645^T^ = DSM 103336^T^), was isolated from alpine meadow soil of Tanggula mountain, China. The DNA G+C content of the type strain is 66.9% (by genome). The GenBank accession numbers for the 16S rRNA gene and genome sequences of the type strain are KU535675 and GCA_009755805.1, respectively.

## Description of *Parasphingobium* gen. nov

*Parasphingobium* (Pa.ra.sphin.go’bi.um. Gr. prep. *para*, next to; N.L. neut. n. *Sphingobium*, a genus name; N.L. neut. n. *Parasphingobium*, a genus next to *Sphingobium*).

Cells are Gram-stain-negative, rod-shaped, non-spore-forming and aerobic. Several members are motile. Positive for catalase. Positive for oxidase. Contain carotenoid pigments but not bacteriochlorophyll *a*. Do not require NaCl for growth. The predominant ubiquinone is Q-10. The major fatty acids (>10%) are summed feature 8 (C_18:1_*ω*7*c* and/or C_18:1_*ω*6*c*) and summed feature 3 (C_16:1_*ω*7*c* and/or C_16:1_*ω*6*c*). The major polar lipids are sphingoglycolipid, phosphatidylglycerol, phosphatidylethanolamine and diphosphatidylglycerol. The genus represents a distinct branch in the family *Sphingobiaceae* of the order *Sphingomonadales* based on the core-genomic phylogeny. The DNA G+C content is 64–66% (by genome). The type species is *Parasphingobium sufflavum*.

## Description of *Parasphingobium lignivorans* comb. nov

*Parasphingobium lignivorans* (lig.ni.vo’rans. L. neut. n. *lignum*, wood; L. pres. part. *vorans*, eating; N.L. part. adj. *lignivorans*, wood eating).

Basonym: *Sphingobium lignivorans* Allemann *et al*. 2023

The description is the same as for *Sphingobium lignivorans* [7]. The type strain, B1D3A^T^ (= ATCC TSD-279^T^ = DSM 111877^T^), was isolated from Hiwassee River sediment near Calhoun, Tennessee, USA. The DNA G+C content of the type strain is 65.6% (by genome). The GenBank accession numbers for the 16S rRNA gene and genome sequences of the type strain are ON306316 and GCA_014203955.1, respectively.

## Description of *parasphingobium sufflavum* comb. nov

*Parasphingobium sufflavum* (suf.fla’vum. N.L. neut. adj. *sufflavum*, light yellow, referring to the production of a yellow pigment).

Basonym: *Sphingobium sufflavum* Sheu *et al*. 2013

The description is the same as for *Sphingobium sufflavum* [139]. The type strain, HL-25^T^ (= BCRC 80413^T^ = DSM 29352^T^ = KCTC 23953^T^), was isolated from Heaven Lake, a freshwater lake located in Southern Cross-Island Highway, Taiwan, China. The DNA G+C content of the type strain is 64.9% (by genome). The GenBank accession numbers for the 16S rRNA gene and genome sequences of the type strain are JQ060960 and GCA_021403115.1, respectively.

## Description of *Parasphingobium xanthum* comb. nov

*Parasphingobium xanthum* (xan’thum. Gr. masc. adj. *xanthos*, yellow; N.L. neut. adj. *xanthum*, yellow).

Basonym: *Sphingobium xanthum* Francis *et al*. 2014

The description is the same as for *Sphingobium xanthum* [40]. The type strain, NL9^T^ (= ATCC 51296^T^ = DSM 100901^T^ = LMG 12560^T^), was isolated from soil using lettuce seedlings as bait in Maasland, The Netherlands. The DNA G+C content of the type strain is 64.6% (by genome). The GenBank accession numbers for the 16S rRNA gene and genome sequences of the type strain are KF437579 and GCA_019737615.1, respectively.

## Description of *Parasphingomonas* gen. nov

*Parasphingomonas* (Pa.ra.sphin.go.mo’nas. Gr. prep. *para*, next to; N.L. fem. n. *Sphingomonas*, a genus name; N.L. fem. n. *Parasphingomonas*, a genus next to *Sphingomonas*).

Cells are Gram-stain-negative, rod-shaped, non-spore-forming and aerobic. Several members are motile. Positive for catalase. Positive or negative for oxidase. Contain carotenoid pigments but not bacteriochlorophyll *a*. Do not require NaCl for growth. The predominant ubiquinone is Q-10. The major fatty acids (>10%) are summed feature 8 (C_18:1_*ω*7*c* and/or C_18:1_*ω*6*c*) and C_16:0_. The major polar lipids are sphingoglycolipid, phosphatidylglycerol, phosphatidylethanolamine and diphosphatidylglycerol. The genus represents a distinct branch in the family *Sphingomonadaceae* of the order *Sphingomonadales* based on the core-genomic phylogeny. The DNA G+C content is 64–69% (by genome). The type species is *Parasphingomonas echinoides*.

## Description of *Parasphingomonas aliaeris* comb. nov

*Parasphingomonas aliaeris* (a.li.a’e.ris. L. masc. pron. *alius*, different; Gr. masc. n. *aêr*, air, atmosphere; N.L. gen. n. *aliaeris*, of a different atmosphere).

Basonym: *Sphingomonas aliaeris* Heidler von Heilborn et al. 2021

The description is the same as for *Sphingomonas aliaeris* [140]. The type strain, DH-S5^T^ (= DSM 110829^T^ = LMG 31606^T^), was isolated from refrigerated pork steak packed under modified atmosphere, containing 20% CO_2_. The DNA G+C content of the type strain is 64.4% (by genome). The GenBank accession numbers for the 16S rRNA gene and genome sequences of the type strain are MT821129 and GCA_016743815.1, respectively.

## Description of *Parasphingomonas alpina* comb. nov

*Parasphingomonas alpina* (al.pi’na. L. fem. adj. *alpina*, of or pertaining to the Alps, Alpine, referring to the isolation of this strain from an alpine environment)

Basonym: *Sphingomonas alpina* Margesin *et al*. 2012

The description is the same as for *Sphingomonas alpina* [141]. The type strain, S8-3^T^ (= DSM 22537^T^ = LMG 26055^T^), was isolated from alpine soil in the Hohe Tauern, Austria. The DNA G+C content of the type strain is 64.0% (by genome). The GenBank accession numbers for the 16S rRNA gene and genome sequences of the type strain are MT821524 and GCA_014490665.1, respectively.

## Description of *Parasphingomonas aquatica* comb. nov

*Parasphingomonas aquatica* (a.qua’ti.ca. L. fem. adj. *aquatica*, living in water, referring to the isolation of the type strain from water).

Basonym: *Sphingomonas aquatica* Choi *et al*. 2017

The description is the same as for *Sphingomonas aquatica* [45]. The type strain, W1-2-1^T^ (= KACC 18309^T^ = LMG 28596^T^), was isolated from tap water in South Korea. The DNA G+C content of the type strain is 67.1% (by genome). The GenBank accession numbers for the 16S rRNA gene and genome sequences of the type strain are KT309085 and GCA_037173185.1, respectively.

## Description of *Parasphingomonas aracearum* comb. nov

*Parasphingomonas aracearum* (a.ra.ce.a’rum. N.L. gen. pl. n. *aracearum*, of representatives of plants belonging to the *Araceae* family).

Basonym: *Sphingomonas aracearum* Wang *et al*. 2019

The description is the same as for *Sphingomonas aracearum* [142]. The type strain, WZY27^T^ (= CCTCC AB 2018056^T^ = KCTC 62523^T^), was isolated from rhizospheric soil of *Araceae* plants in Jiugong Mountain, Hubei Province, China. The DNA G+C content of the type strain is 68.3% (by genome).

## Description of *Parasphingomonas echinoides* comb. nov

*Parasphingomonas echinoides* (Gr. masc. n. *echînos*, hedgehog, sea-urchin; L. adj. suff. -*oides*, resembling, similar; from Gr. neut. adj. suff. -*eides*, resembling, similar; from Gr. neut. n. *eîdos*, that which is seen, form, shape, figure; N.L. fem. adj. *echinoides*, prickly, like a hedgehog or a sea-urchin, intended to mean spiny shaped)

Basonym: *Pseudomonas echinoides* Heumann 1962 (Approved Lists 1980)

Homotypic synonym: *Sphingomonas echinoides* (Heumann 1962) Denner *et al*. 1999

The description is the same as for *Sphingomonas echinoides* [36,143, 144]. The type strain, DSM 1805^T^ (= ATCC 14820^T^ = CCUG 2870^T^ = CIP 103301^T^ = ICPB 2835^T^ = LMG 7462^T^ = NBRC 15742^T^ = NRRL B-3126^T^), was isolated as a laboratory contaminant on a nutrient agar plate. The DNA G+C content of the type strain is 64.7% (by genome). The GenBank accession numbers for the 16S rRNA gene and genome sequences of the type strain are AB021370 and GCA_000241465.1, respectively.

## Description of *Parasphingomonas glacialis* comb. nov

*Parasphingomonas glacialis* (gla.ci.a’lis. L. fem. adj. *glacialis*, icy, frozen, full of ice, referring to the frozen, icy environment from which the type strain was isolated)

Basonym: *Sphingomonas glacialis* Zhang *et al*. 2011

The description is the same as for *Sphingomonas glacialis* [145]. The type strain, C16y^T^ (= CGMCC 1.8957^T^ = CIP 110131^T^ = DSM 22294^T^), was isolated from a glacier cryoconite collected from the Stubai Glacier, Austria. The DNA G+C content of the type strain is 65.7% (by genome). The GenBank accession number for the genome sequences of the type strain is GCA_014653575.1.

## Description of *Parasphingomonas hylomeconis* comb. nov

*Parasphingomonas hylomeconis* (hy.lo.me.co’nis. N.L. gen. n. *hylomeconis*, of the plant genus *Hylomecon*)

Basonym: *Sphingomonas hylomeconis* Akbar *et al*. 2015

The description is the same as for *Sphingomonas hylomeconis* [146]. The type strain, GZJT-2^T^ (= CCTCC AB 2013304^T^ = KCTC 42739^T^), was isolated from the surface-sterilized stem of *Hylomecon japonica* collected from Taibai Mountain in Shaanxi Province, north-west China. The DNA G+C content of the type strain is 65.7% (by genome). The GenBank accession numbers for the 16S rRNA gene and genome sequences of the type strain are KF551120 and GCA_025370105.1, respectively.

## Description of *Parasphingomonas panacis* comb. nov

*Parasphingomonas panacis* (pa’na.cis. L. gen. n. *panacis*, of *Panax ginseng*, the host plant from which the type strain of this species was isolated)

Basonym: *Sphingomonas panacis* Singh e*t al*. 2017

The description is the same as for *Sphingomonas panacis* [36,147]. The type strain, DCY99^T^ (= CCTCC AB 2015192^T^ = JCM 30806^T^ = KCTC 42347^T^), was isolated from the surface of rusty mountain ginseng at Hwacheon Mountain of Gangwon Province, Republic of Korea. The DNA G+C content of the type strain is 65.5% (by genome). The GenBank accession number for the genome sequences of the type strain is GCA_001717955.1.

## Description of *Parasphingomonas populi* comb. nov

*Parasphingomonas populi* (po’pu.li. L. gen. n. *populi*, of the poplar tree)

Basonym: *Sphingomonas populi* Li *et al*. 2020

The description is the same as for *Sphingomonas populi* [148]. The type strain, 3-7^T^ (= CFCC 11561^T^ = LMG 30138^T^), was isolated from bark tissue of *Populus × euramericana*. The DNA G+C content of the type strain is 65.1% (by genome).

## Description of *Parasphingomonas psychrolutea* comb. nov

*Parasphingomonas psychrolutea* (psy.chro.lu’te.a. Gr. masc. adj. *psychros*, cold; L. masc. adj. *luteus*, orange-yellow; N.L. fem. adj. *psychrolutea*, cold and orange-yellow)

Basonym: *Sphingomonas psychrolutea* Liu *et al*. 2015

The description is the same as for *Sphingomonas psychrolutea* [149]. The type strain, MDB1-A^T^ (= CGMCC 1.10106^T^ = NBRC 109639^T^), was isolated from the ice of Midui glacier, Tibet, China. The DNA G+C content of the type strain is 64.2% (by genome). The GenBank accession numbers for the 16S rRNA gene and genome sequences of the type strain are KR258737 and GCA_014636175.1, respectively.

## Description of *Parasphingomonas qilianensis* comb. nov

*Parasphingomonas qilianensis* (qi.lian.en’sis. N.L. fem. adj. *qilianensis*, referring to Qilian Mountains, where the type strain was isolated).

Basonym: *Sphingomonas qilianensis* Piao *et al*. 2016

The description is the same as for *Sphingomonas qilianensis* [150]. The type strain, X1^T^ (= CGMCC 1.15349^T^ = KCTC 42862^T^), was isolated from the ice of Midui glacier, Tibet, China. The DNA G+C content of the type strain is 64.7% (by genome). The GenBank accession numbers for the 16S rRNA gene and genome sequences of the type strain are KT000387 and GCA_039614825.1, respectively.

## Description of *Parastakelama* gen. nov

*Parastakelama* (Pa.ra.sta.ke.la’ma. Gr. prep. *para*, next to; N.L. fem. n. *Stakelama*, a genus name; N.L. fem. n. *Parastakelama*, a genus next to *Stakelama*)

Cells are Gram-stain-negative, rod-shaped, non-spore-forming and aerobic. Several members are motile. Positive for catalase. Positive or negative for oxidase. Contain carotenoid pigments but not bacteriochlorophyll *a*. Do not require NaCl for growth. The predominant ubiquinone is Q-10. The major fatty acids (>10%) are summed feature 8 (C_18:1_*ω*7*c* and/or C_18:1_*ω*6*c*). The major polar lipids are phosphatidylcholine, sphingoglycolipid, phosphatidylglycerol and phosphatidylethanolamine. The genus represents a distinct branch in the family *Sphingomonadaceae* of the order *Sphingomonadales* based on the core-genomic phylogeny. The DNA G+C content is 64–69% (by genome). The type species is *Parastakelama japonica*.

## Description of *Parastakelama aestuarii* comb. Nov

*Parastakelama aestuarii* (aes.tu.a’ri.i. L. gen. n. *aestuarii*, of a tidal flat).

Basonym: *Sphingomonas aestuarii* Roh *et al*. 2009

The description is the same as for *Sphingomonas aestuarii* [36,151]. The type strain, K4^T^ (= CIP 110056^T^ = DSM 19475^T^ = KCTC 22050^T^), was isolated from tidal flat sediment in Yeosu, Korea. The DNA G+C content of the type strain is 64.5% (by genome). The GenBank accession number for the 16S rRNA gene sequences of the type strain is EF660755. The type strain genome accession number deposited into the Global Catalogue of Type Strain database is GCM10014947.

## Description of *Parastakelama baiyangensis* comb. nov

*Parastakelama baiyangensis* (bai.yang.en’sis. N.L. fem. adj. *baiyangensis*, pertaining to Baiyang Lake, in the north China plain, from where the type strain was isolated).

Basonym: *Sphingomonas baiyangensis* Wei *et al*. 2022

The description is the same as for *Sphingomonas baiyangensis* [152]. The type strain, L-1–4 w-11^T^ (= CGMCC 1.13572^T^ = JCM 33962^T^), was isolated from water in Baiyang Lake, China. The DNA G+C content of the type strain is 67.8% (by genome).

## Description of *Parastakelama japonica* comb. nov

*Parastakelama japonica* (ja.po’ni.ca. N.L. fem. adj. *japonica*, Japanese, pertaining to the Sea of Japan, the place from which the type strain was isolated).

Basonym: *Sphingomonas japonica* Romanenko *et al*. 2009

The description is the same as for *Sphingomonas japonica* [25,153]. The type strain, KC7^T^ (= CIP 110395^T^ = DSM 22753^T^ = JCM 15438^T^ = KMM 3038^T^ = NRIC 0738^T^), was isolated from a specimen of the crab *Paralithodes camtschatica* collected from Peter the Great Bay, Sea of Japan, Russia. The DNA G+C content of the type strain is 66.3% (by genome). The GenBank accession numbers for the 16S rRNA gene and genome sequences of the type strain are AB428568 and GCA_006346325.1, respectively.

## Description of *Parastakelama spermidinifaciens* comb. nov

*Parastakelama spermidinifaciens* (sper.mi.di.ni.fa’ci.ens. N.L. neut. n. *spermidinum*, spermidine; L. pres. part. *faciens*, making; N.L. part. adj. *spermidinifaciens*, producing spermidine)

Basonym: *Sphingomonas spermidinifaciens* Feng *et al*. 2017

The description is the same as for *Sphingomonas spermidinifaciens* [16,25]. The type strain, 9 NM-10^T^ (= DSM 27571^T^ = GDMCC 1.657^T^), was isolated from an abandoned lead–zinc mine in Meizhou, Guangdong Province, China. The DNA G+C content of the type strain is 68.0% (by genome). The GenBank accession numbers for the 16S rRNA gene and genome sequences of the type strain are JQ608324 and GCA_002351485.1, respectively.

## Description of *Parastakelama yantingensis* comb. nov

*Parastakelama yantingensis* (yan.ting.en’sis. N.L. fem. adj. *yantingensis*, pertaining to Yanting County in China, where the type strain was isolated).

Basonym: *Sphingomonas yantingensis* Huang *et al*. 2014

The description is the same as for *Sphingomonas yantingensis* [25,154]. The type strain, 1007^T^ (= CCTCC AB 2013146^T^ = DSM 27244^T^ = JCM 19201^T^), was isolated from a specimen of the crab *Paralithodes camtschatica* collected from a purplish soil sample collected in Yanting (Sichuan, China). The DNA G+C content of the type strain is 68.2% (by genome). The GenBank accession numbers for the 16S rRNA gene and genome sequences of the type strain are JX566547 and GCA_014199325.1, respectively.

## Description of *Pseudoblastomonas* gen. nov

*Pseudoblastomonas* (Pseu.do.blas.to.mo’nas. Gr. masc. adj. *pseudês*, false; N.L. fem. n. *Blastomonas*, a genus name; N.L. fem. n. *Pseudoblastomonas*, the false *Blastomonas*).

Cells are Gram-stain-negative, rod- or ovoid-shaped, non-spore-forming and aerobic or facultatively anaerobic. Motile. Positive for catalase. Positive for oxidase. Contain carotenoid pigments. Several members contain bacteriochlorophyll *a*. Several members require NaCl for growth. The predominant ubiquinone is Q-10. The major fatty acids (>10%) are C_17:1_*ω*6*c*, summed features 3 (C_16:1_*ω*7*c* and/or iso-C_15:0_ 2-OH) and summed feature 8 (C_18:1_*ω*7*c* and/or C_18:1_*ω*6*c*). The major polar lipids are diphosphatidylglycerol, phosphatidylcholine, phosphatidylethanolamine, phosphatidylglycerol, phosphatidylinositol and sphingoglycolipid. The genus represents a distinct branch in the family *Erythrobacteraceae* of the order *Sphingomonadales* based on the core-genomic phylogeny. The DNA G+C content is 63–65% (by genome). The type species is *Pseudoblastomonas marina*.

## Description of *Pseudoblastomonas halimionae* comb. nov

*Pseudoblastomonas halimionae* (ha.li.mi.o’nae. N.L. gen. n. *halimionae*, of the marsh plant *Halimione portulacoides*)

Basonym: *Altererythrobacter halimionae* Fidalgo *et al*. 2017

Homotypic synonym: *Alteriqipengyuania halimionae* (Fidalgo *et al*. 2017) Xu *et al*. 2020

The description is the same as for *Alteriqipengyuania halimionae* [5,155]. The type strain, CPA5^T^ (= CECT 9130^T^ = LMG 29519^T^ = UCCCB 42^T^), was isolated from the surface-sterilized aboveground tissues of the halophyte *Halimione portulacoides*. The DNA G+C content of the type strain is 63.6% (by genome). The GenBank accession numbers for the 16S rRNA gene and genome sequences of the type strain are KY310593 and GCA_009827575.1, respectively.

## Description of *Pseudoblastomonas marina* comb. nov

*Pseudoblastomonas marina* (ma.ri’na. L. fem. adj. *marina*, of the sea, marine. Referring to the isolation source of this microorganism, seawater)

Basonym: *Blastomonas marina* Meng *et al*. 2017

The description is the same as for *Blastomonas marina* [156]. The type strain, SSR2A-4-2^T^ (= CGMCC 1.15297^T^ = DSM 103453^T^), was isolated from coastal water in the East China Sea near Taizhou in Zhejiang province of China. The DNA G+C content of the type strain is 64.9% (by genome). The GenBank accession numbers for the 16S rRNA gene and genome sequences of the type strain are KX250272 and GCA_014641655.1, respectively.

## Description of *Pseudonovosphingobium* gen. nov

*Pseudonovosphingobium* (Pseu.do.no.vo.sphin.go’bi.um. Gr. masc. adj. *pseudês*, false; N.L. neut. n. *Novosphingobium*, a genus name; N.L. fem. n. *Pseudonovosphingobium*, the false *Novosphingobium*).

Cells are Gram-stain-negative, rod-shaped, non-spore-forming and aerobic or facultatively anaerobic. Several members are motile. Positive or negative for catalase. Positive or negative for oxidase. Contain carotenoid pigments but not bacteriochlorophyll *a*. Several members require NaCl for growth. The predominant ubiquinone is Q-10. The major fatty acids (>10%) are summed feature 8 (C_18:1_*ω*7*c* and/or C_18:1_*ω*6*c*). The major polar lipids are diphosphatidylglycerol, phosphatidylcholine, phosphatidylethanolamine, phosphatidylglycerol and sphingoglycolipid. The genus represents a distinct branch in the family *Erythrobacteraceae* of the order *Sphingomonadales* based on the core-genomic phylogeny. The DNA G+C content is 62–69% (by genome). The type species is *Pseudonovosphingobium resinovorum*.

## Description of *Pseudonovosphingobium aquimarinum* comb. nov

*Pseudonovosphingobium aquimarinum* (a.qui.ma.ri’num. L. fem. n. *aqua*, water; L. masc. adj. *marinus*, marine, of the sea; N.L. neut. adj. *aquimarinum*, of seawater).

Basonym: *Novosphingobium aquimarinum* Le *et al*. 2020

The description is the same as for *Novosphingobium aquimarinum* [157]. The type strain, M24A2M^T^ (= JCM 33983^T^ = KCTC 72894^T^), was isolated from seawater of the South Sea, Republic of Korea. The DNA G+C content of the type strain is 67.6% (by genome).

## Description of *Pseudonovosphingobium aureum* comb. nov

*Pseudonovosphingobium aureum* (au’re.um. L. neut. adj. *aureum*, gold-coloured).

Basonym: *Novosphingobium aureum* Yoo *et al*. 2021

The description is the same as for *Novosphingobium aureum* [158]. The type strain, YJ- S2-02^T^ (= JCM 33996^T^ = KACC 21677^T^ = KCTC 72891^T^), was isolated from salt flat sediment collected in Yongyu-do, Republic of Korea. The DNA G+C content of the type strain is 65.5% (by genome). The GenBank accession numbers for the 16S rRNA gene and genome sequences of the type strain are MW307329 and GCA_015865035.1, respectively.

## Description of *Pseudonovosphingobium barchaimii* comb. nov

*Pseudonovosphingobium barchaimii* (bar.cha.im’i.i. N.L. gen. n. *barchaimii*, of Yechiel Bar-Chaim, who has served as a programme director for various Central European and North African countries for the American Jewish Joint Distribution Committee, a philanthropic and humanitarian aid organization)

Basonym: *Novosphingobium barchaimii* Niharika *et al*. 2013

The description is the same as for *Novosphingobium barchaimii* [36,159]. The type strain, LL02^T^ (= CCM 7980^T^ = DSM 25411^T^), was isolated from hexachlorocyclohexane-contaminated soil at Spolana Neratovice in the Czech Republic. The DNA G+C content of the type strain is 64.0% (by genome). The GenBank accession numbers for the 16S rRNA gene and genome sequences of the type strain are JN695619 and GCA_001046635.1, respectively.

## Description of *Pseudonovosphingobium chloroacetimidivorans* comb. nov

*Pseudonovosphingobium chloroacetimidivorans* (chlo.ro.a.ce.ti.mi.di.vo’rans. N.L. neut. n. *chloroacetimidum*, chloroacetimide herbicide; L. pres. part. *vorans*, devouring; N.L. part. adj. *chloroacetimidivorans*, chloroacetimide herbicide-devouring, degrading)

Basonym: *Novosphingobium chloroacetimidivorans* Chen *et al*. 2014

The description is the same as for *Novosphingobium chloroacetimidivorans* [21]. The type strain, BUT-14^T^ (= BCRC 80813^T^ = CCTCC AB 2013086^T^ = DSM 100345^T^ = JCM 19923^T^ = KACC 17147^T^), was isolated from activated sludge of chloroacetamide herbicides-manufacturing wastewater treatment facility from Kunshan city, Jiangsu province, China. The DNA G+C content of the type strain is 65.3% (by genome). The GenBank accession number for the 16S rRNA gene sequences of the type strain is KF676669. The type strain genome accession number deposited into the Global Catalogue of Type Strain database is GCM10030111.

## Description of *Pseudonovosphingobium clariflavum* comb. nov

*Pseudonovosphingobium clariflavum* (cla.ri.fla’vum. L. masc. adj. *clarus*, clear, bright, shining, brilliant; L. masc. adj. *flavus*, yellow; N.L. neut. adj. *clariflavum*, bright yellow, the colour of the colonies)

Basonym: *Novosphingobium clariflavum* Zhang *et al*. 2017

The description is the same as for *Novosphingobium clariflavum* [160]. The type strain, 164^T^ (= CICC 11035s^T^ = DSM 103351^T^), was isolated from a used sponge for equipment cleaning at a household product plant in China. The DNA G+C content of the type strain is 65.7% (by genome). The GenBank accession numbers for the 16S rRNA gene and genome sequences of the type strain are KU530129 and GCA_026420865.1, respectively.

## Description of *Pseudonovosphingobium colocasiae* comb. nov

*Pseudonovosphingobium colocasiae* (co.lo.ca’si.ae. L. fem. n. *Colocasia*, the name of a botanical genus (taro); L. gen. n. *colocasiae*, of taro, denoting the isolation of the type strain from taro)

Basonym: *Novosphingobium colocasiae* Chen *et al*. 2016

The description is the same as for *Novosphingobium colocasiae* [161]. The type strain, Teta-03^T^ (= KCTC 32255^T^ = LMG 27385^T^), was isolated from a taro field in Luye Township in the vicinity of Taitung city, eastern Taiwan, China. The DNA G+C content of the type strain is 65.2% (by genome). The GenBank accession numbers for the 16S rRNA gene and genome sequences of the type strain are HF548595 and GCA_014652555.1, respectively.

## Description of *Pseudonovosphingobium decolorationis* comb. nov

*Pseudonovosphingobium decolorationis* (de.co.lo.ra.ti.o’nis. L. gen. n. *decolorationis*, of discolouration).

Basonym: *Novosphingobium decolorationis* Chen *et al*. 2021

The description is the same as for *Novosphingobium decolorationis* [9]. The type strain, 502str22^T^ (= KCTC 82134^T^ = MCCC 1K04799^T^), was isolated from sediment sampled from the East Pacific Ocean. The DNA G+C content of the type strain is 65.5% (by genome). The GenBank accession numbers for the 16S rRNA gene and genome sequences of the type strain are MN940439 and GCA_018417475.1, respectively.

## Description of *Pseudonovosphingobium endophyticum* comb. nov

*Pseudonovosphingobium endophyticum* (en.do.phy’ti.cum. Gr. pref. *endo*-, within; Gr. neut. n. *phyton*, plant; L. neut. adj. suff. -*icum*, adjectival suffix used with the sense of belonging to; N.L. neut. adj. *endophyticum*, within plant, endophytic pertaining to the original isolation from plant tissues).

Basonym: *Novosphingobium endophyticum* Li *et al*. 2016

The description is the same as for *Novosphingobium endophyticum* [162]. The type strain, EGI 60015^T^ (= CGMCC 1.15095^T^ = DSM 29948^T^= JCM 30707^T^ = KCTC 42486^T^), was isolated from healthy roots of *Glycyrrhiza uralensis* F. collected from Yili County, Xinjiang Province, Northwest China. The DNA G+C content of the type strain is 64.2% (by genome). The GenBank accession number for the genome sequences of the type strain is GCA_014640675.1.

## Description of *Pseudonovosphingobium fluoreni* comb. nov

*Pseudonovosphingobium fluoreni* (flu.o.re’ni. N.L. neut. n. *fluorenum*, fluorene; N.L. gen. neut. n. *fluoreni*, of fluorene).

Basonym: *Novosphingobium fluoreni* Gao *et al*. 2015

The description is the same as for *Novosphingobium fluoreni* [163]. The type strain, HLJ-RS18^T^ (= ACCC 19180^T^ = DSM 27568^T^), was isolated from rice seeds collected from Heilongjiang Province, China. The DNA G+C content of the type strain is 63.4% (by genome). The GenBank accession numbers for the 16S rRNA gene and genome sequences of the type strain are KF460450 and GCA_014196615.1, respectively.

## Description of *Pseudonovosphingobium gossypii* comb. nov

*Pseudonovosphingobium gossypii* (gos.sy’pi.i. N.L. gen. n. *gossypii*, of *Gossypium hirsutum*).

Basonym: *Novosphingobium gossypii* Kämpfer *et al*. 2015

The description is the same as for *Novosphingobium gossypii* [164]. The type strain, JM-1396^T^ (= CCM 8569^T^ = CIP 110884^T^ = DSM 29615^T^ = LMG 28605^T^), was isolated from the healthy internal stem tissue of post-harvest cotton (*Gossypium hirsutum*, cultivar ‘DES-119’) grown at the Plant Breeding Unit at the E. V. Smith Research Centre in Tallassee (Macon county), AL, USA. The DNA G+C content of the type strain is 63.4% (by genome). The GenBank accession number for the 16S rRNA gene sequences of the type strain is KP657488. The type strain genome accession number deposited into the Global Catalogue of Type Strain database is GCM10007347.

## Description of *Pseudonovosphingobium guangzhouense* comb. nov

*Pseudonovosphingobium guangzhouense* (guang.zhou.en’se. N.L. neut. adj. *guangzhouense*, pertaining to Guangzhou, the place of isolation of the type strain).

Basonym: *Novosphingobium guangzhouense* Sha *et al*. 2017

The description is the same as for *Novosphingobium guangzhouense* [36,165]. The type strain, SA925^T^ (= DSM 32207^T^ = GDMCC 1.1110^T^), was isolated from oil-polluted soil in a refinery located in Guangzhou, Guangdong Province, China. The DNA G+C content of the type strain is 62.7% (by genome). The GenBank accession numbers for the 16S rRNA gene and genome sequences of the type strain are KX215153 and GCA_002896965.1, respectively.

## Description of *Pseudonovosphingobium indicum* comb. nov

*Pseudonovosphingobium indicum* (in’di.cum. L. neut. adj. *indicum*, Indian, referring to the Indian Ocean, where the type strain was isolated).

Basonym: *Novosphingobium indicum* Yuan *et al*. 2009

The description is the same as for *Novosphingobium indicum* [166]. The type strain, H25^T^ (= CGMCC 1.6784^T^ = DSM 23608^T^ = LMG 24713^T^ = MCCC 1A01080^T^), was isolated from deep-sea water of the Indian Ocean. The DNA G+C content of the type strain is 63.1% (by genome). The GenBank accession numbers for the 16S rRNA gene and genome sequences of the type strain are EF549586 and GCA_014645195.1, respectively.

## Description of *Pseudonovosphingobium lindaniclasticum* comb. nov

*Pseudonovosphingobium lindaniclasticum* (lin.da.ni.clas’ti.cum. N.L. neut. n. *lindanum*, lindane; N.L. masc. adj. *clasticus*, breaking; from Gr. masc. adj. *klastos*, broken, in pieces; N.L. neut. adj. *lindaniclasticum*, lindane-breaking).

Basonym: *Novosphingobium lindaniclasticum* Saxena *et al*. 2013

The description is the same as for *Novosphingobium lindaniclasticum* [36,167]. The type strain, LE124^T^ (= CCM 7976^T^ = DSM 25409^T^), was isolated from an hexachlorocyclohexane-contaminated dumpsite in Lucknow, India. The DNA G+C content of the type strain is 64.6% (by genome). The GenBank accession numbers for the 16S rRNA gene and genome sequences of the type strain are JN687581 and GCA_000445125.1, respectively.

## Description of *Pseudonovosphingobium malaysiense* comb. nov

*Pseudonovosphingobium malaysiense* (mal.ay.si.en’se. N.L. neut. adj. *malaysiense*, belonging or pertaining to Malaysia, the source of the soil from which the type strain was isolated).

Basonym: *Novosphingobium malaysiense* Lee *et al*. 2014

The description is the same as for *Novosphingobium malaysiense* [168]. The type strain, MUSC 273^T^ (= DSM 27798^T^ = MCCC 1A00645^T^ = NBRC 109947^T^), was isolated from mangrove soil collected from the state of Pahang in peninsular Malaysia. The DNA G+C content of the type strain is 63.4% (by genome). The GenBank accession numbers for the 16S rRNA gene and genome sequences of the type strain are KC907395 and GCA_000802225.1, respectively.

## Description of *Pseudonovosphingobium marinum* comb. nov

*Pseudonovosphingobium marinum* (ma.ri’num. L. neut. adj. *marinum*, of the sea, marine).

Basonym: *Novosphingobium marinum* Huo *et al*. 2015

The description is the same as for *Novosphingobium marinum* [169]. The type strain, LA53^T^ (= CGMCC 1.12918^T^ = DSM 29043^T^ = JCM 30307^T^), was isolated from a seawater sample of the Eastern Pacific Ocean. The DNA G+C content of the type strain is 64.2% (by genome). The GenBank accession numbers for the 16S rRNA gene and genome sequences of the type strain are KJ708552 and GCA_014640055.1, respectively.

## Description of *Pseudonovosphingobium mathurense* comb. nov

*Pseudonovosphingobium mathurense* (ma.thu.ren’se. N.L. neut. adj. *mathurense*, pertaining to Mathura, the place of isolation of the type strain).

Basonym: *Novosphingobium mathurense* Gupta *et al*. 2009

The description is the same as for *Novosphingobium mathurense* [36,170]. The type strain, SM117^T^ (= CCM 7473^T^ = MTCC 9020^T^), was isolated from oil-contaminated soil. The DNA G+C content of the type strain is 63.3% (by genome). The GenBank accession numbers for the 16S rRNA gene and genome sequences of the type strain are EF424403 and GCA_900168325.1, respectively.

## Description of *Pseudonovosphingobium naphthalenivorans* comb. nov

*Pseudonovosphingobium naphthalenivorans* (naph.tha.le.ni.vo’rans. N.L. neut. n. *naphthalenum*, naphthalene; L. pres. part. *vorans*, devouring; N.L. part. adj. *naphthalenivorans*, naphthalene-devouring).

Basonym: *Novosphingobium naphthalenivorans* Suzuki and Hiraishi 2008

The description is the same as for *Novosphingobium naphthalenivorans* [171]. The type strain, TUT562^T^ (= CCUG 56309^T^ = DSM 18518^T^ = JCM 13951^T^ = NBRC 102051^T^), was isolated from polychlorinated-dioxin-contaminated soil. The DNA G+C content of the type strain is 63.8% (by genome). The GenBank accession numbers for the 16S rRNA gene and genome sequences of the type strain are AB177883 and GCA_001590985.1, respectively.

## Description of *Pseudonovosphingobium panipatense* comb. nov

*Pseudonovosphingobium panipatense* (pa.ni.pa.ten’se. N.L. neut. adj. *panipatense*, pertaining to Panipat, the place of isolation of the type strain).

Basonym: *Novosphingobium panipatense* Gupta *et al*. 2009

The description is the same as for *Novosphingobium panipatense* [170]. The type strain, SM16^T^ (= CCM 7472^T^ = MTCC 9019^T^), was isolated from oil-contaminated soil. The DNA G+C content of the type strain is 64.2% (by genome). The GenBank accession number for the 16S rRNA gene sequences of the type strain is EF424402. The type strain genome accession number deposited into the Global Catalogue of Type Strain database is GCM10011473.

## Description of *Pseudonovosphingobium pentaromativorans* comb. nov

*Pseudonovosphingobium pentaromativorans* (pent.a.ro.ma.ti.vo’rans. Gr. num. *penta*, five; L. gen. n. *aromatis*, of spice; L. pres. part. *vorans*, devouring; N.L. part. adj. *pentaromativorans*, devouring/degrading aromatic compounds with five rings).

Basonym: *Novosphingobium pentaromativorans* Sohn *et al*. 2004

The description is the same as for *Novosphingobium pentaromativorans* [36,172]. The type strain, US6-1^T^ (= CCUG 56449^T^ = CIP 108548^T^ = DSM 17173^T^ = JCM 12182^T^ = KCTC 10454^T^), was isolated from estuarine sediment at Ulsan Bay, Republic of Korea. The DNA G+C content of the type strain is 63.0% (by genome). The GenBank accession numbers for the 16S rRNA gene and genome sequences of the type strain are AF502400 and GCA_000767465.1, respectively.

## Description of *Pseudonovosphingobium resinovorum* comb. nov

*Pseudonovosphingobium resinovorum* (re.si.no.vo’rum. L. fem. n. *resina*, resin or gum of trees; L. inf. v. *vorare*, to devour; N.L. neut. adj. *resinovorum*, resin-devouring).

Basonym: *Flavobacterium resinovorum* Delaporte and Daste 1956 (Approved Lists 1980)

Homotypic synonym: *Novosphingobium resinovorum* (Delaporte and Daste 1956) Lim *et al*. 2007

The description is the same as for *Novosphingobium resinovorum* [173,174]. The type strain, DSM 7478^T^ (= ATCC 33545^T^ = CIP 109724^T ^= LMG 8367^T^ = VKM B-1172^T^). The DNA G+C content of the type strain is 65.3% (by genome). The GenBank accession numbers for the 16S rRNA gene and genome sequences of the type strain are EF029110 and GCA_027922145.1, respectively.

## Description of *Pseudonovosphingobium silvae* comb. nov

*Pseudonovosphingobium silvae* (sil’vae. L. gen. n. *silvae*, of a forest).

Basonym: *Novosphingobium silvae* Feng *et al*. 2020

The description is the same as for *Novosphingobium silvae* [175]. The type strain, FGD1^T^ (= GDMCC 1.1761^T^ = KACC 21283^T^), was isolated from subtropical forest soil of the Nanling National Forest Park located in Guangdong Province, China. The DNA G+C content of the type strain is 65.1% (by genome).

## Description of *Pseudonovosphingobium soli* comb. nov

*Pseudonovosphingobium soli* (so’li. L. gen. n. *soli*, of soil, the source of the type strain).

Basonym: *Novosphingobium soli* Kämpfer *et al*. 2011

The description is the same as for *Novosphingobium soli* [176]. The type strain, CC-TPE-1^T^ (= CCM 7706^T^ = CCUG 58493^T^ = CIP 110292^T^ = DSM 22821^T^), was isolated from oil-contaminated soil near an oil refinery located in Kaohsiung County, Taiwan, China. The DNA G+C content of the type strain is 68.5% (by genome). The GenBank accession number for the 16S rRNA gene sequences of the type strain is FJ425737. The type strain genome accession number deposited into the Global Catalogue of Type Strain database is GCM10020017.

## Description of *Pseudopontixanthobacter muriae* comb. nov

*Pseudopontixanthobacter muriae* (mu’ri.ae. L. gen. n. *muriae*, of brine, substance from which the strain was isolated).

Basonym: *Altererythrobacter muriae* Azpiazu-Muniozguren *et al*. 2019

Homotypic synonym: *Alteripontixanthobacter muriae* (Azpiazu-Muniozguren *et al*. 2019) Kim *et al*. 2021

The description is the same as for *Alteripontixanthobacter muriae* [117,177]. The type strain, SALINAS58^T^ (= CECT 30029^T^ = LMG 31726^T^), was isolated from the hypersaline water of a natural spring in the saltern of the Añana Salt Valley, Spain. The DNA G+C content of the type strain is 61.4% (by genome). The GenBank accession numbers for the 16S rRNA gene and genome sequences of the type strain are MN918268 and GCA_013336795.1, respectively.

## Description of *Pseudosphingomonas* gen. nov

*Pseudosphingomonas* (Pseu.do.sphin.go.mo’nas. Gr. masc./fem. adj. *pseudês*, false; N.L. fem. n. *Sphingomonas*, a genus name; N.L. fem. n. *Pseudosphingomonas*, the false *Sphingomonas*).

Cells are Gram-stain-negative, rod- or ovoid-shaped, non-spore-forming and aerobic. Several members are motile. Positive or negative for catalase. Positive or negative for oxidase. Colonies are white or pink-, red-, orange- and yellow-pigmented. Several members contain bacteriochlorophyll *a*. Do not require NaCl for growth. The predominant ubiquinone is Q-10. The major fatty acids (>10%) are summed feature 8 (C_18:1_*ω*7*c* and/or C_18:1_*ω*6*c*). The major polar lipids are diphosphatidylglycerol and sphingoglycolipid. The genus represents a distinct branch in the family *Sphingomicrobiaceae* of the order *Sphingomonadales* based on the core-genomic phylogeny. The DNA G+C content is 62–69% (by genome). The type species is *Pseudosphingomonas jaspsi*.

## Description of *Pseudosphingomonas agri* comb. nov

*Pseudosphingomonas agri* (a’gri. L. gen. n. *agri*, of a field).

Basonym: *Sphingomonas agri* Siddiqi *et al*. 2017

The description is the same as for *Sphingomonas agri* [44]. The type strain, HKS-06^T^ (= KACC 18880^T^ = LMG 29563^T^), was isolated from field soil of Hankyong National University, South Korea. The DNA G+C content of the type strain is 62.7% (by genome). The GenBank accession numbers for the 16S rRNA gene and genome sequences of the type strain are KT950747 and GCA_038148795.1, respectively.

## Description of *Pseudosphingomonas arenae* comb. nov

*Pseudosphingomonas arenae* (a.re’nae. L. gen. n. *arenae*, of sand, referring to the isolation source of the type strain).

Basonym: *Sphingomonas arenae* Dong *et al*. 2022

The description is the same as for *Sphingomonas arenae* [12]. The type strain, SYSU D00720^T^ (= MCCC 1K05154^T^ = NBRC 115061^T^), was isolated from a sandy soil sample collected from the Gurbantunggut Desert in Xinjiang, north-west China. The DNA G+C content of the type strain is 66.0% (by genome). The GenBank accession numbers for the 16S rRNA gene and genome sequences of the type strain are MT527641 and GCA_016924655.1, respectively.

## Description of *Pseudosphingomonas astaxanthinifaciens* comb. nov

*Pseudosphingomonas astaxanthinifaciens* (as.ta.xan.thi.ni.fa’ci.ens. N.L. neut. n. *astaxanthinum*, astaxanthin; L. pres. part. *faciens*, making/producing; N.L. part. adj. *astaxanthinifaciens*, astaxanthin-producing).

Basonym: *Sphingomonas astaxanthinifaciens* Asker *et al*. 2008

The description is the same as for *Sphingomonas astaxanthinifaciens* [178]. The type strain, TDMA-17^T^ (= CCUG 53608^T^ = DSM 22298^T^ = NBRC 102146^T^), was isolated from a freshwater sample collected at Misasa (Tottori, Japan). The DNA G+C content of the type strain is 68.4% (by genome). The GenBank accession numbers for the 16S rRNA gene and genome sequences of the type strain are AB681719 and GCA_000711715.1, respectively.

## Description of *Pseudosphingomonas daechungensis* comb. nov

*Pseudosphingomonas daechungensis* (dae.chung.en’sis. N.L. fem. adj. *daechungensis*, pertaining to Daechung Reservoir where the type strain was isolated).

Basonym: *Sphingomonas daechungensis* Huy *et al*. 2014

The description is the same as for *Sphingomonas daechungensis* [179]. The type strain, CH15-11^T^ (= DSM 29999^T^ = JCM 17887^T^ = KCTC 23718^T^), was isolated from sediment from Daechung Reservoir, South Korea. The DNA G+C content of the type strain is 63.0% (by genome). The GenBank accession numbers for the 16S rRNA gene and genome sequences of the type strain are JQ772481 and GCA_039543105.1, respectively.

## Description of *Pseudosphingomonas edaphi* comb. nov

*Pseudosphingomonas edaphi* (e’da.phi. Gr. neut. n. *edaphos*, soil; N.L. gen. n. *edaphi*, from soil).

Basonym: *Sphingomonas edaphi* Kim *et al*. 2020

The description is the same as for *Sphingomonas edaphi* [180]. The type strain, DAC4^T^ (= JCM 32377^T^ = KCTC 62107^T^), was isolated from a soil sample collected at Ahnmok Beach, Busan, Republic of Korea. The DNA G+C content of the type strain is 62.2% (by genome).

## Description of *Pseudosphingomonas ginkgonis* comb. nov

*Pseudosphingomonas ginkgonis* (gink.go’nis. N.L. gen. n. *ginkgonis*, of *Ginkgo biloba*)

Basonym: *Sphingomonas ginkgonis* Cha *et al*. 2019

The description is the same as for *Sphingomonas ginkgonis* [181]. The type strain, HMF7854^T^ (= KCTC 62461^T^ = NBRC 113337^T^), was isolated from a ginkgo tree in Yongin, Republic of Korea. The DNA G+C content of the type strain is 68.4% (by genome).

## Description of *Pseudosphingomonas ginsengisoli* comb. nov

*Pseudosphingomonas ginsengisoli* (gin.sen.gi.so’li. N.L. neut. n. *ginsengum*, ginseng; L. neut. n. *solum*, soil; N.L. gen. n. *ginsengisoli*, of soil of a ginseng field)

Basonym: *Sphingomonas ginsengisoli* An *et al*. 2013

The description is the same as for *Sphingomonas ginsengisoli* [182]. The type strain, Gsoil 634^T^ (= DSM 18094^T^ = KCTC 12630^T^ = LMG 23739^T^), was isolated from soil collected in a ginseng field in Pocheon province, South Korea. The DNA G+C content of the type strain is 67.2% (by genome). The GenBank accession numbers for the 16S rRNA gene and genome sequences of the type strain are AB245347 and GCA_003332855.1, respectively.

## Description of *Pseudosphingomonas jaspsi* comb. nov

*Pseudosphingomonas jaspsi* (ja.sp’si. N.L. neut. n. *jaspsum*, arbitrary name derived from the acronym JSPS (Japan Society for the Promotion of Science); N.L. gen. n. *jaspsi*, of JSPS, the organization that supported this study).

Basonym: *Sphingomonas jaspsi* Asker *et al*. 2007

The description is the same as for *Sphingomonas jaspsi* [36,183]. The type strain, TDMA-16^T^ (= CCUG 53607^T^ = CIP 109619^T^ = DSM 18422^T^ = NBRC 102120^T^), was isolated from a freshwater sample collected at Misasa (Tottori, Japan). The DNA G+C content of the type strain is 64.7% (by genome). The GenBank accession numbers for the 16S rRNA gene and genome sequences of the type strain are AB681706 and GCA_000585415.1, respectively.

## Description of *Pseudosphingomonas kaistensis* comb. nov

*Pseudosphingomonas kaistensis* (ka.is.ten’sis. N.L. fem. adj. *kaistensis*, of or pertaining to the Korea Advanced Institute of Science and Technology (KAIST)).

Basonym: *Sphingomonas kaistensis* Kim *et al*. 2007

The description is the same as for *Sphingomonas kaistensis* [184]. The type strain, PB56^T^ (= CIP 110410^T^ = DSM 16846^T^ = KCTC 12334^T^), was isolated from soil on the campus of KAIST in Daejeon city in South Korea. The DNA G+C content of the type strain is 67.0% (by genome). The GenBank accession numbers for the 16S rRNA gene and genome sequences of the type strain are AY769083 and GCA_011927725.1, respectively.

## Description of *Pseudosphingomonas lutea* comb. nov

*Pseudosphingomonas lutea* (lu’te.a. L. fem. adj. *lutea*, yellow-coloured).

Basonym: *Sphingomonas lutea* Lee *et al*. 2016

The description is the same as for *Sphingomonas lutea* [185]. The type strain, JS5^T^ (= CCTCC AB 2017135^T^ = JCM 18309^T^ = KCTC 23642^T^), was isolated from freshwater of Juam reservoir, Republic of Korea. The DNA G+C content of the type strain is 65.3% (by genome). The GenBank accession numbers for the 16S rRNA gene and genome sequences of the type strain are JF922305 and GCA_014396785.1, respectively.

## Description of *Pseudosphingomonas mesophila* comb. nov

*Pseudosphingomonas mesophila* (me.so’phi.la. Gr. masc. adj. *mesos*, medium; Gr. masc. adj. *philos*, loving; N.L. fem. adj. *mesophila*, medium-temperature-loving, mesophilic).

Basonym: *Sphingomonas mesophila* Li *et al*. 2019

The description is the same as for *Sphingomonas mesophila* [186]. The type strain, SYSUP0001^T^ (= CGMCC 1.16462^T^ = KCTC 62179^T^), was isolated from the tubers of *Gastrodia elata* Blume collected from Yunnan province, China. The DNA G+C content of the type strain is 67.5% (by genome).

## Description of *Pseudosphingomonas piscis* comb. nov

*Pseudosphingomonas piscis* (pis’cis. L. gen. n. *piscis*, of a fish, from which the strain was first isolated).

Basonym: *Sphingomonas piscis* Hyun *et al*. 2022

The description is the same as for *Sphingomonas piscis* [20]. The type strain, HDW15B^T^ (= JCM 33738^T^ = KACC 21341^T^ = KCTC 72588^T^), was isolated from the intestine of the leopard mandarin fish *Siniperca scherzeri*. The DNA G+C content of the type strain is 63.3% (by genome).

## Description of *Pseudosphingomonas rhizophila* comb. nov

*Pseudosphingomonas rhizophila* (rhi.zo’phi.la. Gr. fem. n. *rhiza*, root; N.L. masc. adj. suff. -*philus*, loving; from Gr. masc. adj. *philos*, loving; N.L. fem. adj. *rhizophila*, root-loving).

Basonym: *Sphingomonas rhizophila* Yan *et al*. 2018

The description is the same as for *Sphingomonas rhizophila* [187]. The type strain, THG-T61^T^ (= CCTCC AB 2016245^T^ = KACC 19189^T^), was isolated from the rhizosphere of *Hibiscus syriacus* collected from Kyung Hee University, Yongin, Republic of Korea. The DNA G+C content of the type strain is 65.2% (by genome). The GenBank accession numbers for the 16S rRNA gene and genome sequences of the type strain are KY287249 and GCA_014396585.1, respectively.

## Description of *Pseudosphingomonas sabuli* comb. nov

*Pseudosphingomonas sabuli* (sa’bu.li. L. gen. n. *sabuli*, of sand, referring to its origin from beach sand).

Basonym: *Sphingomonas sabuli* Kang *et al*. 2021

The description is the same as for *Sphingomonas sabuli* [188]. The type strain, sand 1–3^T^ (= KCTC 82358^T^ = NBRC 114538^T^), was isolated from beach sand sampled at Haeundae Beach, Busan, Republic of Korea. The DNA G+C content of the type strain is 65.9% (by genome). The GenBank accession numbers for the 16S rRNA gene and genome sequences of the type strain are MN577362 and GCA_014352855.1, respectively.

## Description of *Pseudosphingomonas segetis* comb. nov

*Pseudosphingomonas segetis* (se.ge’tis. L. gen. n. *segetis*, of the soil, referring to the isolation site).

Basonym: *Sphingomonas segetis* Lee and Whang 2020

The description is the same as for *Sphingomonas segetis* [189]. The type strain, YJ09^T^ (= KACC 19551^T^ = NBRC 113247^T^), was isolated from a spinach-farming field located on Bigeum Island in Shinan, south-west Republic of Korea. The DNA G+C content of the type strain is 65.9% (by genome).

## Description of *Pseudosphingomonas sinipercae* comb. nov

*Pseudosphingomonas sinipercae* (si.ni.per’cae. N.L. gen. n. *sinipercae*, of *Siniperca*, the name of the genus of fish, from which the strain was first isolated).

Basonym: *Sphingomonas sinipercae* Hyun *et al*. 2022

The description is the same as for *Sphingomonas sinipercae* [20]. The type strain, HDW15C^T^ (= JCM 33739^T^ = KACC 21342^T^ = KCTC 72589^T^), was isolated from the intestine of the leopard mandarin fish *Siniperca scherzeri*. The DNA G+C content of the type stran is 65.5% (by genome).

## Description of *Pseudostakelama* gen. nov

*Pseudostakelama* (Pseu.do.sta.ke.la’ma. Gr. masc. adj. *pseudês*, false; N.L. fem. n. *Stakelama*, a genus name; N.L. fem. n. *Pseudostakelama*, the false *Stakelama*).

Cells are Gram-stain-negative, rod-shaped, non-spore-forming and aerobic. Several members are motile. Positive for catalase. Positive for oxidase. Colonies are white or orange-pigmented. Do not require NaCl for growth. The predominant ubiquinone is Q-10. The major fatty acids (>10%) are summed feature 8 (C_18:1_*ω*7*c* and/or C_18:1_*ω*6*c*). The major polar lipids are diphosphatidylglycerol, phosphatidylethanolamine, phosphatidylglycerol, sphingoglycolipid and phosphatidylcholine. The genus represents a distinct branch in the family *Sphingomonadaceae* of the order *Sphingomonadales* based on the core-genomic phylogeny. The DNA G+C content is 66–69% (by genome). The type species is *Pseudostakelama guangdongensis*.

## Description of *Pseudostakelama cannabina* comb. nov

*Pseudostakelama cannabina* (can.na’bi.na. L. fem. adj. *cannabina*, of hemp, of *Cannabis*, the generic name of the host plant, *Cannabis sativa* L. ‘Cheungsam’)

Basonym: *Sphingomonas cannabina* Jeon *et al*. 2022

The description is the same as for *Sphingomonas cannabina* [19]. The type strain, DM2-R-LB4^T^ (= GDMCC 1.3018^T^ = KCTC 92075^T^), was isolated from *Cannabis sativa* L. from Andong, Republic of Korea. The DNA G+C content of the type strain is 67.9% (by genome).

## Description of *Pseudostakelama floccifaciens* comb. nov

*Pseudostakelama floccifaciens* (floc.ci.fa’ci.ens. L. masc. n. *floccus*, floc; L. pres. part. *faciens*, making, forming; N.L. part. adj. *floccifaciens*, forming flocs).

Basonym: *Sphingomonas floccifaciens* Fan *et al*. 2019

The description is the same as for *Sphingomonas floccifaciens* [190]. The type strain, FQM01^T^ (= CGMCC 1.15797^T^ = KCTC 52630^T^), was isolated from a subterranean sediment sample in the Mohe permafrost area, China. The DNA G+C content of the type strain is 66.3% (by genome). The GenBank accession number for the 16S rRNA gene sequences of the type strain is KX022124. The type strain genome accession number deposited into the Global Catalogue of Type Strain database is GCM10020312.

## Description of *Pseudostakelama guangdongensis* comb. nov

*Pseudostakelama guangdongensis* (guang.dong.en’sis. N.L. fem. adj. *guangdongensis*, pertaining to Guangdong, a province of south China).

Basonym: *Sphingomonas guangdongensis* Feng *et al*. 2014

The description is the same as for *Sphingomonas guangdongensis* [36,191]. The type strain, 9 NM-8^T^ (= CGMCC 1.12672^T^ = DSM 27570^T^ = GDMCC 1.653^T^), was isolated from an abandoned lead-zinc ore of a mining area in Mei county, Meizhou, Guangdong province, China. The DNA G+C content of the type strain is 68.6% (by genome). The GenBank accession numbers for the 16S rRNA gene and genome sequences of the type strain are JQ608326 and GCA_900199185.1, respectively.

## Description of *Qipengyuania atrilutea* comb. nov

*Qipengyuania atrilutea* (at.ri.lu’te.a. L. masc. adj. *ater*, dark; L. masc. adj. *luteus*, orange; N.L. fem. adj. *atrilutea*, dark orange).

Basonym: *Actirhodobacter atriluteus* Xue *et al*. 2021

The description is the same as for *Actirhodobacter atriluteus* [192]. The type strain, HHU K3-1^T^ (= CGMCC 1.17395^T^ = KCTC 72834^T^ = MCCC 1K04225^T^), was isolated from the surface water of Yellow Sea, China. The DNA G+C content of the type strain is 62.1% (by genome).

## Description of *Qipengyuania lutea* nom. nov

*Qipengyuania lutea* (lu’te.a. L. fem. adj. *lutea*, orange coloured).

Basonym: *Altererythrobacter aurantiacus* Zhang *et al*. 2016

Homotypic synonym: *Parapontixanthobacter aurantiacus* (Zhang *et al*. 2016) Xu *et al*. 2020

The description is the same as for *Parapontixanthobacter aurantiacus* [5,193]. The type strain, O30^T^ (= CGMCC 1.12762^T^ = JCM 19853^T^ = LMG 28110^T^ = MCCC 1A09962^T^), was isolated from a deep-sea sediment of the west Pacific Ocean. The DNA G+C content of the type strain is 61.2% (by genome). The GenBank accession numbers for the 16S rRNA gene and genome sequences of the type strain are KF924607 and GCA_009827635.1, respectively.

## Description of *Rhizorhabdus crocodyli* comb. nov

*Rhizorhabdus crocodyli* (cro.co.dy’li. N.L. gen. n. *crocodyli*, of the genus *Crocodylus*, of the crocodile).

Basonym: *Sphingomonas crocodyli* Sheu *et al*. 2019

The description is the same as for *Sphingomonas crocodyli* [194]. The type strain, CCP-7^T^ (= BCRC 81096^T^ = KCTC 62190^T^ = LMG 30311^T^), was isolated from a freshwater pond in the Chaozhou Township in Pingtung County, Taiwan. The DNA G+C content of the type strain is 64.5% (by genome).

## Description of *Rhizorhabdus montanisoli* comb. nov

*Rhizorhabdus montanisoli* (mon.ta.ni.so’li. L. masc. adj. *montanus*, of a mountain; L. neut. n. *solum*, soil; N.L. gen. n. *montanisoli*, of mountain soil).

Basonym: *Sphingomonas montanisoli* Xu *et al*. 2020

The description is the same as for *Sphingomonas montanisoli* [195]. The type strain, ZX9611^T^ (= CCTCC AB 2019350^T^ = KCTC 72622^T^), was isolated from mountain soil, located in Jiaozuo, Henan province, China. The DNA G+C content of the type strain is 64.8% (by genome).

## Description of *Sandarakinorhabdus fusca* comb. nov

*Sandarakinorhabdus fusca* (fus’ca. L. fem. adj. *fusca*, brown).

Basonym: *Polymorphobacter fuscus* Jia *et al*. 2015

The description is the same as for *Polymorphobacter fuscus* [196]. The type strain, D40P^T^ (= CGMCC 1.12714^T^ = DSM 105347^T^ = JCM 19740^T^), was isolated from a permafrost soil sample of Kunlun Mountain Gap (Qinghai-Tibet Plateau, China). The DNA G+C content of the type strain is 66.8% (by genome). The GenBank accession numbers for the 16S rRNA gene and genome sequences of the type strain are KF737330 and GCA_009607675.1, respectively.

## Description of *Sandarakinorhabdus glacialis* comb. nov

*Sandarakinorhabdus glacialis* (gla.ci.a’lis. L. fem. adj. *glacialis*, icy, full of ice, referring to the frozen, icy environment from which the type strain was isolated).

Basonym: *Polymorphobacter glacialis* Xing *et al*. 2017

The description is the same as for *Polymorphobacter glacialis* [197]. The type strain, B555-2^T^ (= CGMCC 1.15519^T^ = KCTC 52396^T^), was isolated from an ice core in Muztagh Glacier, Tibet, China. The DNA G+C content of the type strain is 65.5% (by genome). The GenBank accession numbers for the 16S rRNA gene and genome sequences of the type strain are KP013180 and GCA_014643455.1, respectively.

## Description of *Solisphingomonas* gen. nov

*Solisphingomonas* (So.li.sphin.go.mo’nas. L. neut. n. *solum*, soil; N.L. fem. n. *Sphingomonas*, a bacterial genus; N.L. fem. n. *Solisphingomonas*, a *Sphingomonas*-like organism isolated from soil).

Cells are Gram-stain-negative, rod-shaped, non-spore-forming and aerobic. Non-motile. Positive or negative for catalase. Positive or negative for oxidase. Contain carotenoids but not bacteriochlorophyll *a*. Do not require NaCl for growth. The predominant ubiquinone is Q-10. The major fatty acids (>10%) are summed feature 8 (C_18:1_*ω*7*c* and/or C_18:1_*ω*6*c*). The major polar lipids are diphosphatidylglycerol, phosphatidylglycerol, phosphatidylcholine, phosphatidylethanolamine and sphingoglycolipid. The genus represents a distinct branch in the family *Rhizorhabdaceae* of the order *Sphingomonadales* based on the core-genomic phylogeny. The DNA G+C content is 66–70% (by genome). The type species is *Solisphingomonas oligoaromativorans*.

## Description of *Solisphingomonas chungangi* comb. nov

*Solisphingomonas chungangi* (chung.ang’i. N.L. gen. n. *chungangi*, named after Chung-Ang University, where the taxonomic studies on this species were performed).

Basonym: *Sphingomonas chungangi* Akter and Huq 2020

The description is the same as for *Sphingomonas chungangi* [198]. The type strain, MAH-6^T^ (= CGMCC 1.13654^T^ = KACC 19292^T^), was isolated from soil sampled from a garden located in Anseong, Republic of Korea. The DNA G+C content of the type strain is 66.2% (by genome).

## Description of *Solisphingomonas morindae* comb. nov

*Solisphingomonas morindae* (mo.rin’dae. N.L. gen. n. *morindae*, pertaining to *Morinda citrifolia* (Noni), from which the type strain was isolated).

Basonym: *Sphingomonas morindae* Liu *et al*. 2015

The description is the same as for *Sphingomonas morindae* [199]. The type strain, NBD5^T^ (= CICC 10879^T^ = DSM 29151^T^ = KCTC 42183^T^), was isolated from Noni (*Morinda citrifolia* L.) branches collected from a Noni cultivation field in Sanya, Hainan Province, South China. The DNA G+C content of the type strain is 69.5% (by genome). The GenBank accession numbers for the 16S rRNA gene and genome sequences of the type strain are KJ934256 and GCA_023822065.1, respectively.

## Description of *Solisphingomonas oligoaromativorans* comb. nov

*Solisphingomonas oligoaromativorans* (o.li.go.a.ro.ma.ti.vo’rans. Gr. masc. adj. *oligos*, little, small; L. neut. n. *aroma*, spice, aroma; L. pres. part. *vorans*, devouring; N.L. part. adj. *oligoaromativorans*, degrading a few aromatic compounds).

Basonym: *Sphingomonas oligoaromativorans* Han *et al*. 2014

The description is the same as for *Sphingomonas oligoaromativorans* [200]. The type strain, SY-6^T^ (= DSM 102246^T^ = KACC 12948^T^ = NBRC 105508^T^), was isolated from soil sampled from a forest soil of Gyeryong Mountain National Park in Korea. The DNA G+C content of the type strain is 67.4% (by genome). The GenBank accession numbers for the 16S rRNA gene and genome sequences of the type strain are FJ434127 and GCA_011762195.1, respectively.

## Description of *Solisphingomonas quercus* comb. nov

*Solisphingomonas quercus* (quer’cus. L. gen. n. *quercus*, of an oak).

Basonym: *Sphingomonas quercus* Shen *et al*. 2022

The description is the same as for *Sphingomonas quercus* [201]. The type strain, XMGL2^T^ (= GDMCC 1.2153^T^ = JCM 34441^T^), was isolated from the rhizosphere of *Quercus mongolica*. The DNA G+C content of the type strain is 67.9% (by genome). The GenBank accession numbers for the 16S rRNA gene and genome sequences of the type strain are MZ343576 and GCA_018863195.1, respectively.

## Description of *Stakelama portus* comb. nov

*Stakelama portus* (por’tus. L. gen. n. *portus*, of a port)

Basonym: *Sphingosinithalassobacter portus* Hetharua *et al*. 2019

The description is the same as for *Sphingosinithalassobacter portus* [202]. The type strain, FM6^T^ (= JCM 32714^T^ = MCCC 1K03501^T^), was isolated from surface seawater from Xiamen Port, China. The DNA G+C content of the type strain is 63.7% (by genome). The GenBank accession numbers for the 16S rRNA gene and genome sequences of the type strain are MG798699 and GCA_003171655.1, respectively.

## Description of *Stakelama tenebrarum* comb. nov

*Stakelama tenebrarum* (te.ne.bra’rum. L. gen. pl. n. *tenebrarum*, of the darkness).

Basonym: *Sphingosinithalassobacter tenebrarum* Zheng and Sun 2020

The description is the same as for *Sphingosinithalassobacter tenebrarum* [203]. The type strain, zrk23^T^ (= KCTC 72896^T^ = MCCC 1K04416^T^), was isolated from deep-sea sediment of cold seep, China. The DNA G+C content of the type strain is 64.7% (by genome). The GenBank accession numbers for the 16S rRNA gene and genome sequences of the type strain are MT460676 and GCA_011057975.1, respectively.

## Description of *Yabuuchia* gen. nov

*Yabuuchia* (Ya.bu.u’chi.a. N.L. fem. n. *Yabuuchia*, named in honour of Professor Eiko Yabuuchi who established the order *Sphingomonadales*).

Cells are Gram-stain-negative, rod-shaped, non-spore-forming and aerobic. Several members are motile. Positive for catalase. Positive for oxidase. Contains carotenoids but not bacteriochlorophyll *a*. Do not require NaCl for growth. The predominant ubiquinone is Q-10. The major fatty acids (>10%) are summed feature 8 (C_18:1_*ω*7*c* and/or C_18:1_*ω*6*c*), summed feature 3 (C_16:1_*ω*7*c* and/or C_16:1_*ω*6*c*) and C_14:0_ 2-OH. The major polar lipids are diphosphatidylglycerol, phosphatidylcholine, phosphatidylethanolamine and sphingoglycolipid. The genus represents a distinct branch in the family *Sphingomonadaceae* of the order *Sphingomonadales* based on the core-genomic phylogeny. The DNA G+C content is 62–66% (by genome). The type species is *Yabuuchia boeckii*.

## Description of *Yabuuchia boeckii* comb. nov

*Yabuuchia boeckii* (boe’cki.i. N.L. gen. n. *boeckii*, of Böck, in honour of August Böck (born 1937), a renowned German microbiologist, for his contributions to general microbiology and microbial biochemistry).

Basonym: *Sphingobium boeckii* Chen *et al*. 2013

The description is the same as for *Sphingobium boeckii* [204]. The type strain, 469^T^ (= DSM 25079^T^ = LMG 26901^T^), was isolated from an oligotrophic alpine freshwater lake (Walchensee, Germany). The DNA G+C content of the type strain is 62.0% (by genome). The GenBank accession numbers for the 16S rRNA gene and genome sequences of the type strain are JN591315 and GCA_014199215.1, respectively.

## Description of *Yabuuchia cavernae* comb.nov

*Yabuuchia cavernae* (ca.ver’nae. L. gen. n. *cavernae*, of a cave).

Basonym: *Sphingomonas cavernae* Zhu *et al*. 2022

The description is the same as for *Sphingomonas cavernae* [205]. The type strain, K2R01-6^T^ (= CGMCC 1.13538^T^ = KCTC 82187^T^), was isolated from a rock sample collected from a karst cave in Guizhou province, southwest People’s Republic of China. The DNA G+C content of the type strain is 63.9% (by genome). The GenBank accession number for the genome sequences of the type strain is GCA_003590775.1.

## Description of *Yabuuchia colocasiae* comb. nov

*Yabuuchia colocasiae* (co.lo.ca’si.ae. L. fem. n. *Colocasia*, the name of a botanical genus (taro); L. gen. n. *colocasiae*, of taro, denoting the isolation of the type strain from taro tissue).

Basonym: *Sphingomonas colocasiae* Lin *et al*. 2018

The description is the same as for *Sphingomonas colocasia*e [206]. The type strain, CC-MHH0539^T^ (= BCRC 80933^T^ = JCM 31229^T^), was isolated from taro plant in Taiwan, China. The DNA G+C content of the type strain is 65.6% (by genome). The GenBank accession numbers for the 16S rRNA gene and genome sequences of the type strain are KU248160 and GCA_019880585.1, respectively.

## Description of *Alteriyabuuchia* gen. nov

*Alteriyabuuchia* (Al.te.ri.ya.bu.u’chi.a. L. masc. adj. *alter*, another, other, different; N.L. fem. n. *Yabuuchia*; N.L. fem. n. *Alteriyabuuchia*, another or different *Yabuuchia*)

Cells are Gram-stain-negative, rod- or ovoid-shaped, non-spore-forming and aerobic. Several members are motile. Positive for catalase. Negative for oxidase. Colonies are white or yellow-pigmented. Do not require NaCl for growth. The predominant ubiquinone is Q-10. The major fatty acids (>10%) are summed feature 8 (C_18:1_ *ω*7*c* and/or C_18:1_*ω*6*c*), C_16:0_ and C_14:0_ 2-OH. The major polar lipids are phosphatidylethanolamine, phosphatidylglycerol, diphosphatidylglycerol, phosphatidylcholine and sphingoglycolipid. The genus represents a distinct branch in the family *Sphingomonadaceae* of the order *Sphingomonadales* based on the core-genomic phylogeny. The DNA G+C content is 66–67% (by genome). The type species is *Alteriyabuuchia sanxanigenens*.

## Description of *Alteriyabuuchia sanxanigenens* comb. nov

*Alteriyabuuchia sanxanigenens* (san.xa.ni.ge’nens. N.L. neut. n. *sanxanum*, sanxan gum (an extracellular biopolymer); L. pres. part. *genens*, producing; from L. inf. v. *genere*, to produce; N.L. part. adj. *sanxanigenens*, sanxan gum-producing)

Basonym: *Sphingomonas sanxanigenens* Huang *et al*. 2009

The description is the same as for *Sphingomonas sanxanigenens* [207]. The type strain, NX02^T^ (= CGMCC 1.6417^T^ = CIP 110406^T^ = DSM 19645^T^), was isolated from soil. The DNA G+C content of the type strain is 66.7% (by genome). The GenBank accession numbers for the 16S rRNA gene and genome sequences of the type strain are DQ789172 and GCA_000512205.2, respectively.

## Description of *Alteriyabuuchia zeicaulis* comb. nov

*Alteriyabuuchia zeicaulis* (ze.i.cau’lis. L. fem. n. *zea*, a kind of grain; *Zea mays*; L. masc. n. *caulis*, stem; N.L. gen. n. *zeicaulis*, of the stem of *Zea mays*).

Basonym: *Sphingomonas zeicaulis* Gao *et al*. 2016

The description is the same as for *Sphingomonas zeicaulis* [208]. The type strain, 541^T^ (= CGMCC 1.15008^T^ = DSM 100587^T^), was isolated from surface-sterilized root tissue of maize planted in the Fangshan District of Beijing, People’s Republic of China. The DNA G+C content of the type strain is 66.0% (by genome). The GenBank accession number for the 16S rRNA gene sequences of the type strain is KP172592. The type strain genome accession number deposited into the Global Catalogue of Type Strain database is GCM10014836.

## Description of *Parayabuuchia* gen. nov

*Parayabuuchia* (Pa.ra.ya.bu.u’chi.a. Gr. prep. *para*, next to; N.L. fem. n. *Yabuuchia*, a bacterial genus; N.L. fem. n. *Parayabuuchia*, a genus next to *Yabuuchia*).

Cells are Gram-stain-negative, rod- or ovoid-shaped, non-spore-forming and aerobic. Several members are motile. Positive for catalase. Positive or negative for oxidase. Contain carotenoids but not bacteriochlorophyll *a*. Do not require NaCl for growth. The predominant ubiquinone is Q-10. The major fatty acids (>10%) are summed feature 8 (C_18:1_ *ω*7*c* and/or C_18:1_*ω*6*c*) and summed feature 3 (C_16:1_*ω*7*c* and/or iso-C_15:0_ 2-OH). The major polar lipids are phosphatidylethanolamine, phosphatidylglycerol and sphingoglycolipid. The genus represents a distinct branch in the family *Sphingomonadaceae* of the order *Sphingomonadales* based on the core-genomic phylogeny. The DNA G+C content is 66–69% (by genome). The type species is *Parayabuuchia changbaiensis*.

## Description of *Parayabuuchia changbaiensis* comb. nov

*Parayabuuchia changbaiensis* (chang.bai.en’sis. N.L. fem. adj. *changbaiensis*, pertaining to the Changbai mountains, in the north-east of China, from where the type strain was isolated).

Basonym: *Sphingomonas changbaiensis* Zhang *et al*. 2010

The description is the same as for *Sphingomonas changbaiensis* [209]. The type strain, V2M44^T^ (= CGMCC 1.7057^T^ = CIP 110386^T^ = DSM 25652^T^ = NBRC 104936^T^), was isolated from forest soil. The DNA G+C content of the type strain is 66.3% (by genome). The GenBank accession numbers for the 16S rRNA gene and genome sequences of the type strain are EU682685 and GCA_000974765.1, respectively.

## Description of *Parayabuuchia flavalba* comb. nov

*Parayabuuchia flavalba* (flav.al’ba. L. masc. adj. *flavus*, yellow; L. masc. adj. *albus*, white; N.L. fem. adj. *flavalba*, yellowish-white, referring to the colour of the colonies on LB agar).

Basonym: *Sphingomonas flavalba* Zhou *et al*. 2019

The description is the same as for *Sphingomonas flavalba* [210]. The type strain, ZLT-5^T^ (= CCTCC AB 2018188^T^ = KCTC 62840^T^), was isolated from procymidone-contaminated soil sampled in Nanjing, Jiangsu, China. The DNA G+C content of the type strain is 68.0% (by genome).

## Description of *Parayabuuchia jejuensis* comb. nov

*Parayabuuchia jejuensis* (je.ju.en’sis. N.L. fem. adj. *jejuensis*, of or belonging to Jeju Island in the Republic of Korea, from where the type strain was isolated).

Basonym: *Sphingomonas jejuensis* Park *et al*. 2012

The description is the same as for *Sphingomonas jejuensis* [211]. The type strain, MS-31^T^ (= DSM 27651^T^ = KCTC 23321^T^ = NBRC 107775^T^), was isolated from the marine sponge *Hymeniacidon flavia*. The DNA G+C content of the type strain is 68.6% (by genome). The GenBank accession numbers for the 16S rRNA gene and genome sequences of the type strain are HQ224549 and GCA_011927695.1, respectively.

## Supplementary material

10.1099/ijsem.0.006769 Supplementary Material 1.

10.1099/ijsem.0.006769 Supplementary Material 2.

## Abbreviations

AAI: amino acid identities
ANI: average nucleotide identity
COG: Clusters of Orthologous Groups of proteins
dDDH: digital DNA-DNA hybridization
DSM: Deutsche Sammlung von Mikroorganismen und Zellkulturen GmbH
JCM: Japan Collection of Microorganisms
KEGG: Kyoto Encyclopedia of Genes and Genomes
MCCC: Marine Culture Collection of China
NBRC: Biological Resource Center, National Institute of Technology and Evaluation
RAST: Rapid Annotation using Subsystem Technology
