# Supplementary material for: Genomic-based taxonomic classification of the order Sphingomonadales
Source: Int J Syst Evol Microbiol. 2025 May 14;75(5):006769. doi: 10.1099/ijsem.0.006769 (PMC12210278; doi:10.1099/ijsem.0.006769)

Supplementary Figures

Genomic-based taxonomic classification of the order *Sphingomonadales*

Yuan Wang[1,2,†], Hao You[1,2,†], Yan-Hui Kong[1,3], Cong Sun[4], Lin-Huan Wu[5], Song-Gun Kim[6], Jung-Sook Lee[6], Lin Xu[4,*], Xue-Wei Xu[7,*]

[1]Key Laboratory of Marine Ecosystem Dynamics, Ministry of Natural Resources & Second Institute of Oceanography, Ministry of Natural Resources, Hangzhou 310012, PR China

[2]School of Oceanography, Zhejiang University, Zhoushan 316021, PR China

[3]School of Oceanography, Shanghai Jiao Tong University, Shanghai 200030, PR China

[4]College of Life Sciences and Medicine, Zhejiang Sci-Tech University, Hangzhou 310018, PR China

[5]Institute of Microbiology Chinese Academy of Sciences, Beijing 100101, PR China

[6]Korea Research Institute of Bioscience and Biotechnology, Korean Collection for Type Cultures, Jeongeup 56212, Republic of Korea

[7]National Deep Sea Center, Ministry of Natural Resources, Qingdao 266237, PR China

[†]These authors contributed equally.

[*]Correspondence: Lin Xu (linxu@zstu.edu.cn); Xue-Wei Xu (xuxw@sio.org.cn)

Figure S1. The maximum-likelihood tree based on 16S rRNA gene sequences showing the phylogenetic relationship of Sphingomonadales type strains. Bootstrap values are based on 1,000 replicates; only bootstrap values 0.7 are shown. Bar, 0.01 substitutions per nucleotide position. Red, yellow, blue and purple represent the family *Erythrobacteraceae*, *Sphingomonadaceae*, *Sphingosinicellaceae* and *Zymomonadaceae*, respectively. *Rhodospirillum rubrum* ATCC 11170^{T} was used as an outgroup.

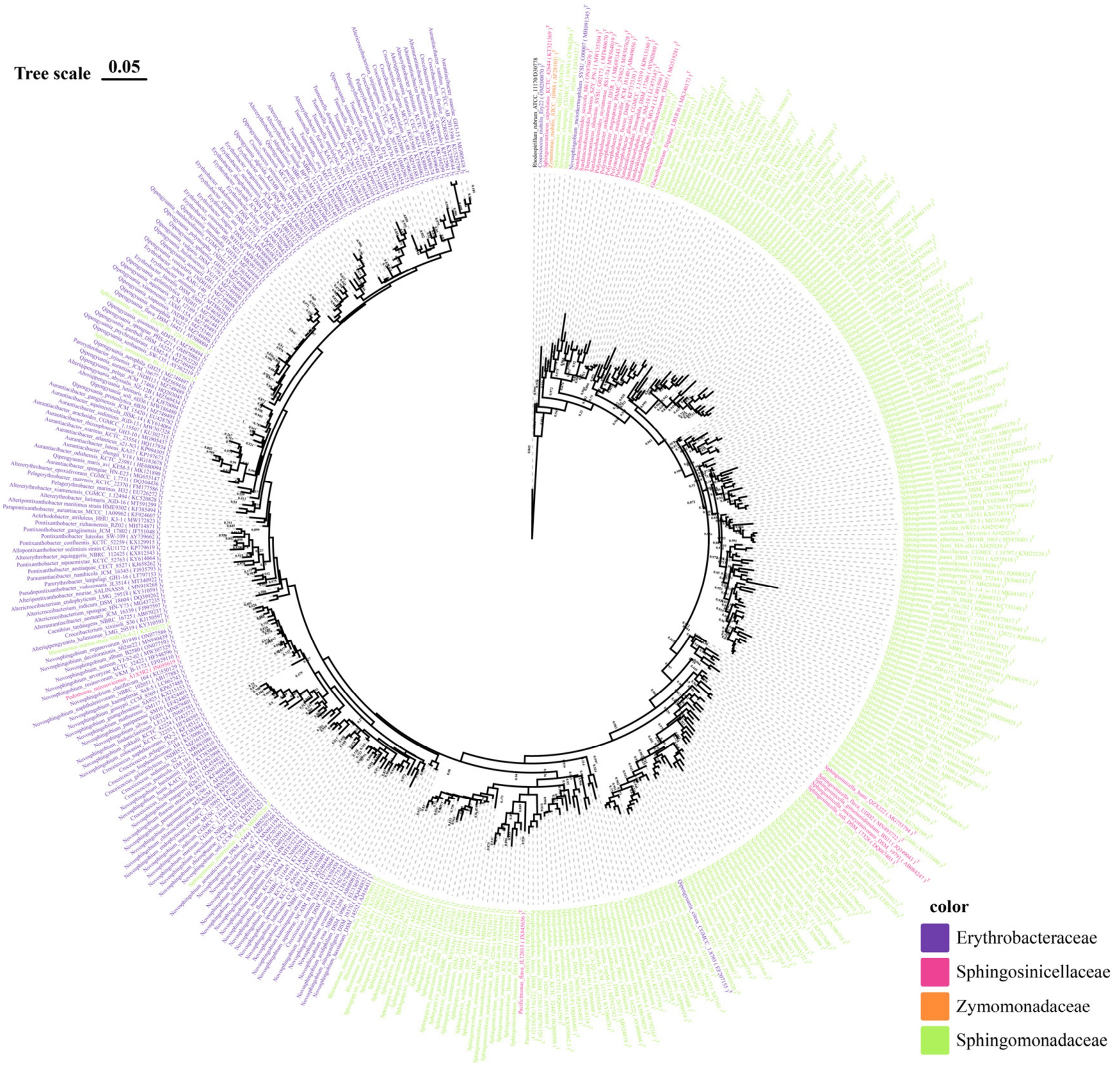

Figure S2. Maximum-likelihood tree based on 120 concatenated protein sequences showing the phylogenetic relationship of type strains belonging to the order *Sphingomonadales*. Bootstrap values are based on 1000 replicates. Bar. 0.1 substitutions per amino acid position. *Rhodospirillum rubrum* ATCC 11170^{T} was used as an outgroup.

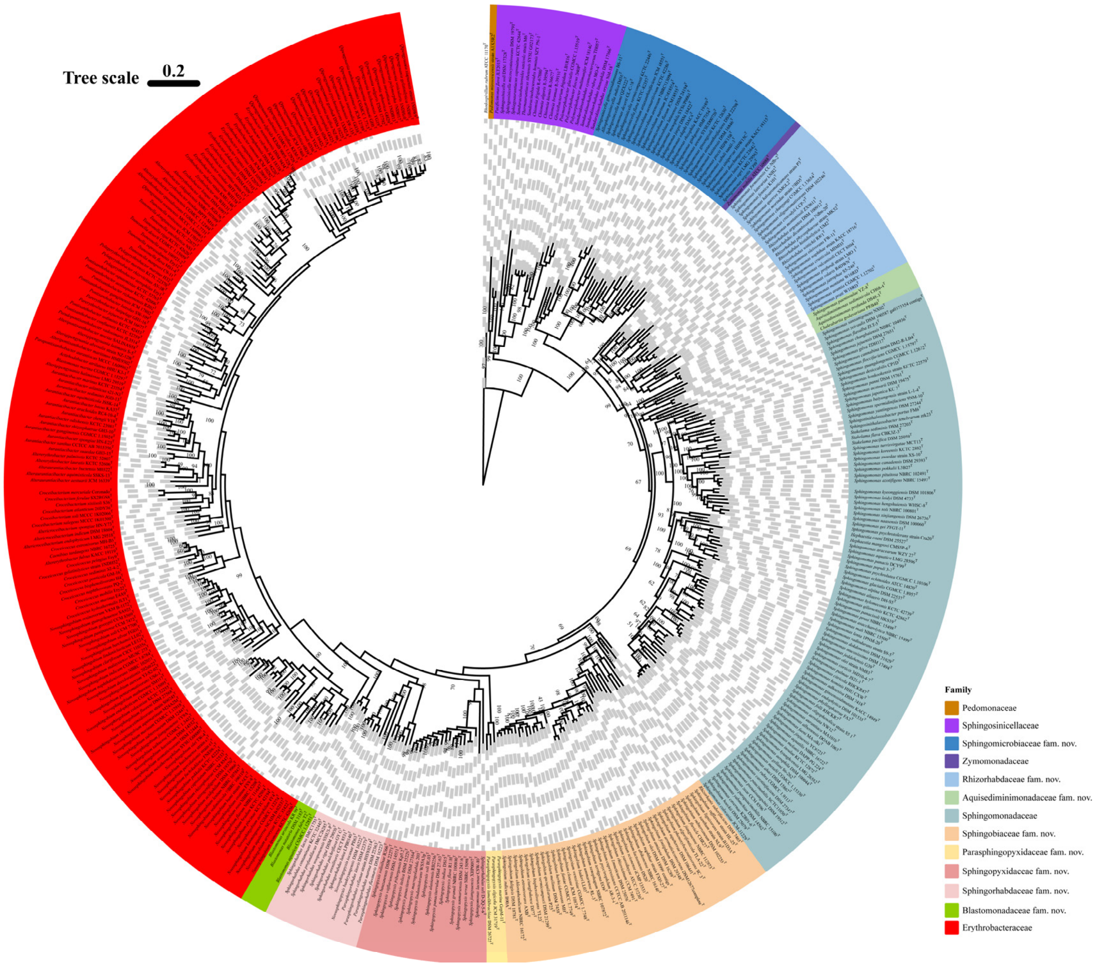

Figure S3. Maximum-likelihood tree based on 22 concatenated protein sequences showing the phylogenetic relationship of type strains belonging to the order *Sphingomonadales*. Bootstrap values are based on 1000 replicates. Bar. 0.1 substitutions per amino acid position. *Rhodospirillum rubrum* ATCC 11170^T was used as an outgroup.

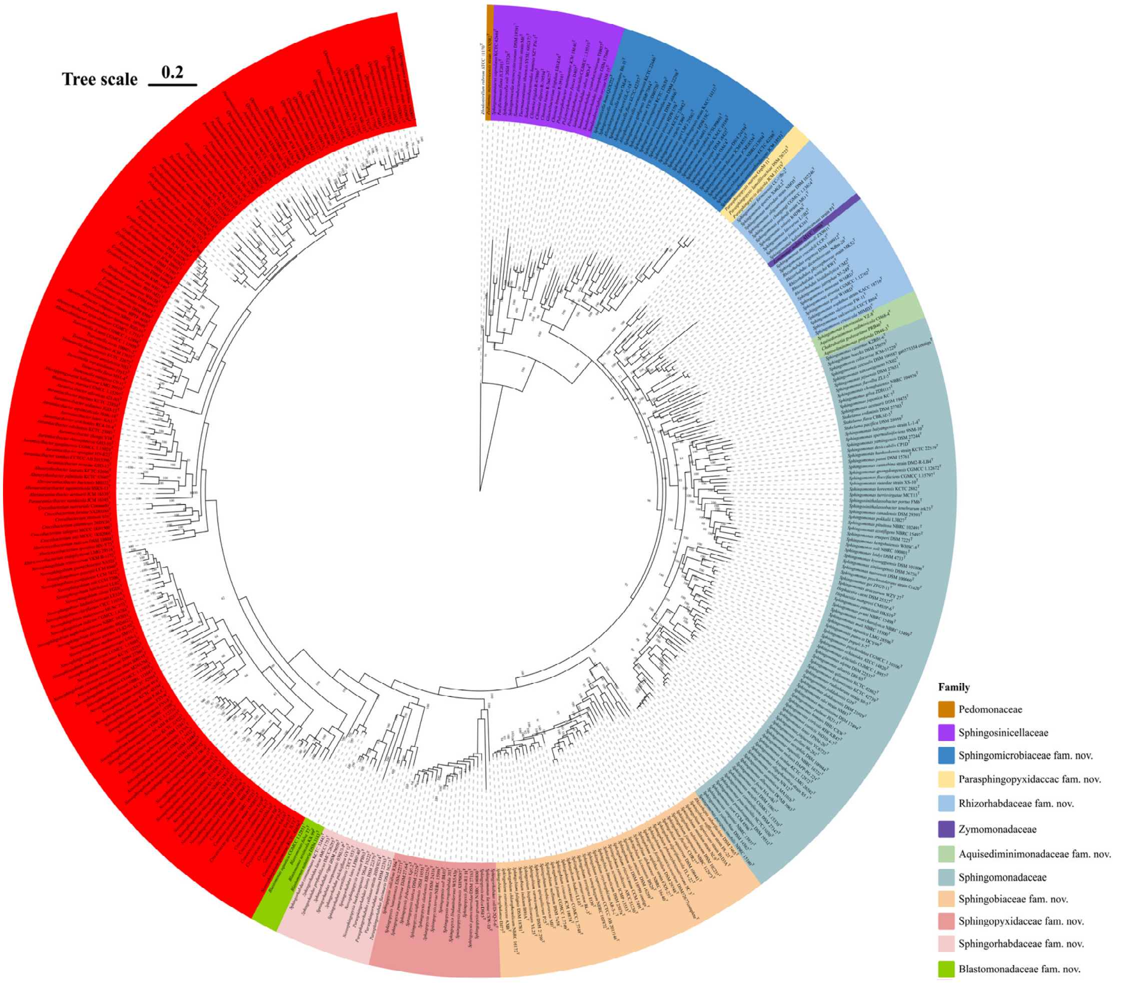

Figure S4. Bayesian tree based on 120 concatenated protein sequences showing the phylogenetic relationship of type strains belonging to the order *Sphingomonadales*. Bootstrap values are based on 1000 replicates. Bar. 0.1 substitutions per amino acid position. *Rhodospirillum rubrum* ATCC 11170^T was used as an outgroup.

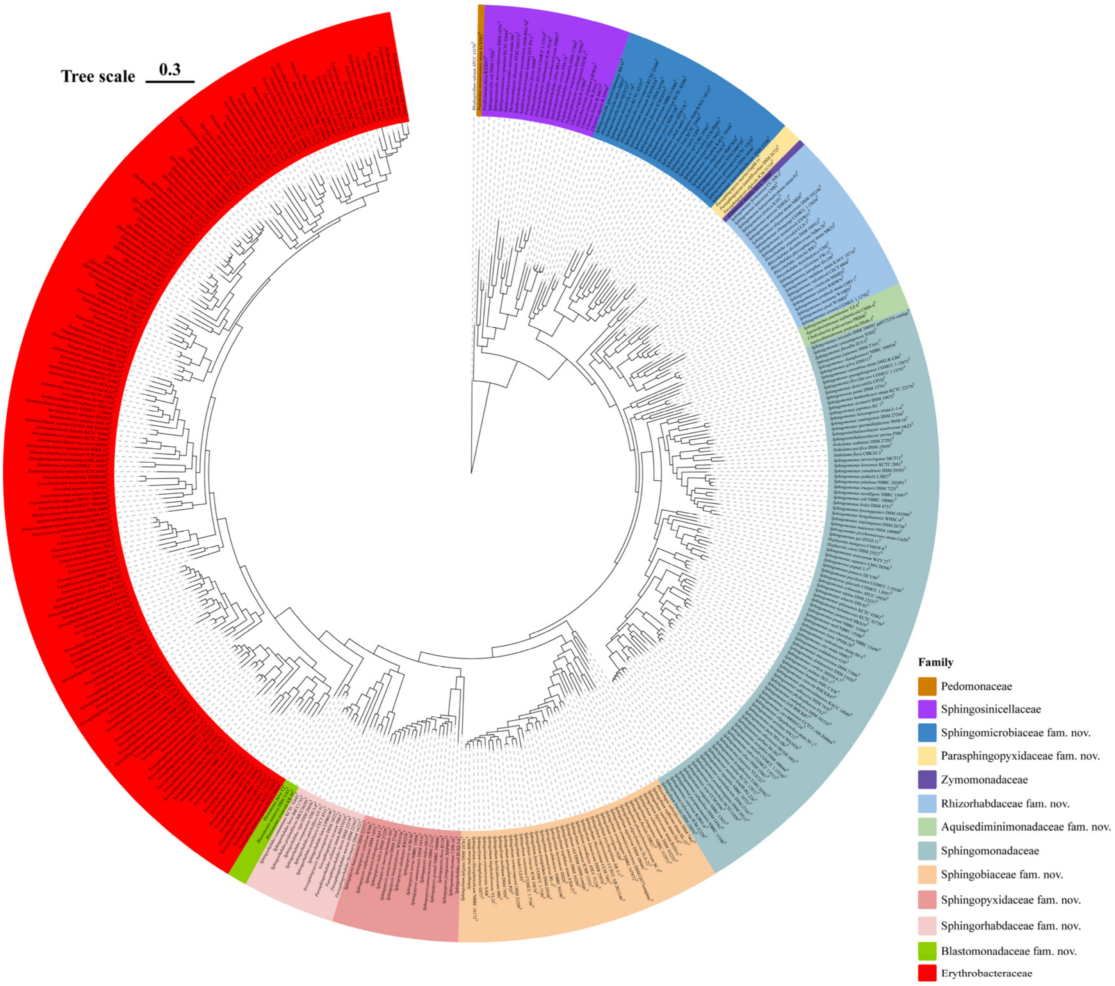

Figure S5. Bayesian tree based on 22 concatenated protein sequences showing the phylogenetic relationship of type strains belonging to the order *Sphingomonadales*. Bootstrap values are based on 1000 replicates. Bar. 0.1 substitutions per amino acid position. *Rhodospirillum rubrum* ATCC 11170^{T} was used as an outgroup.

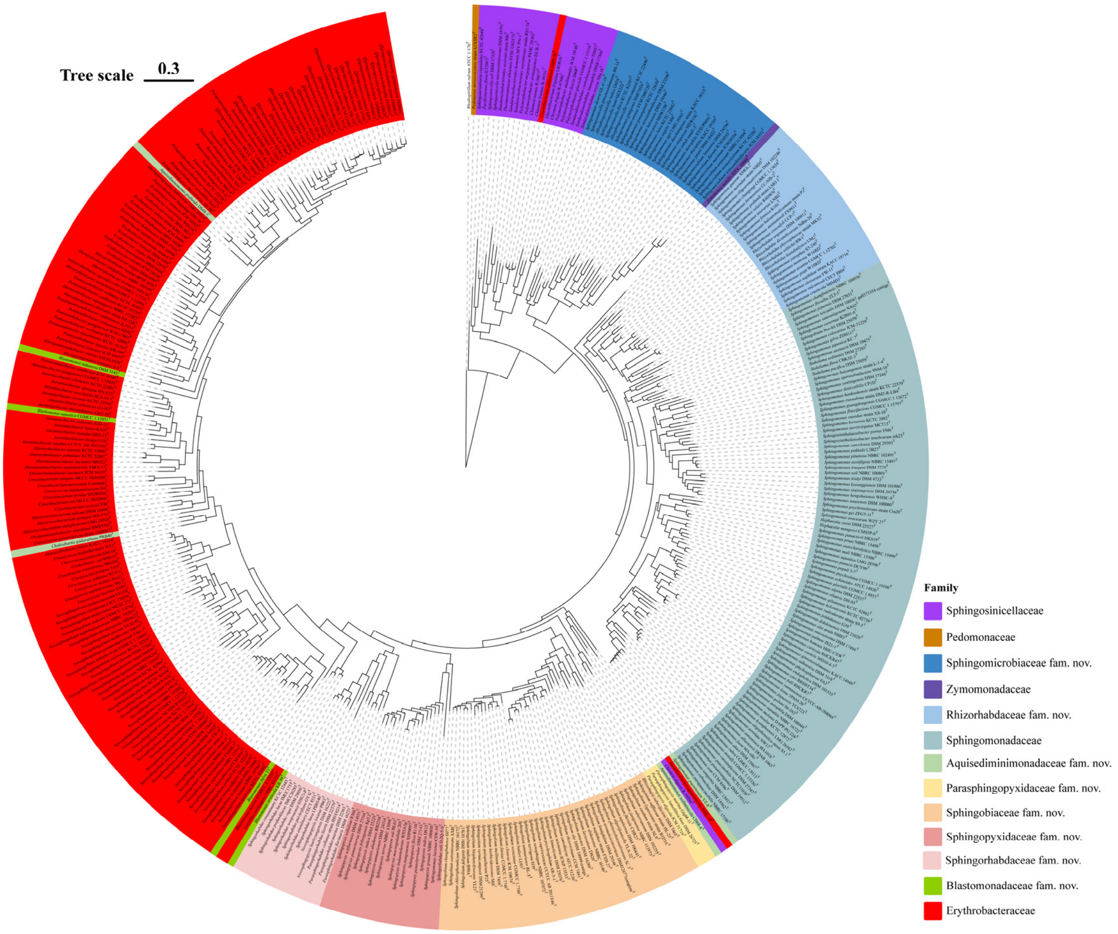

Supplement: Supplementary Material 1. [file ijsem-75-06769-s001.pdf]
